# A Monoclonal Antibody Toolkit for *C. elegans*

**DOI:** 10.1371/journal.pone.0010161

**Published:** 2010-04-13

**Authors:** Gayla Hadwiger, Scott Dour, Swathi Arur, Paul Fox, Michael L. Nonet

**Affiliations:** 1 Department of Anatomy and Neurobiology, Washington University School of Medicine, St. Louis, Missouri, United States of America; 2 Department of Genetics, Washington University School of Medicine, St. Louis, Missouri, United States of America; Harvard University, United States of America

## Abstract

**Background:**

Antibodies are critical tools in many avenues of biological research. Though antibodies can be produced in the research laboratory setting, most research labs working with vertebrates avail themselves of the wide array of commercially available reagents. By contrast, few such reagents are available for work with model organisms.

**Methodology/Principal Findings:**

We report the production of monoclonal antibodies directed against a wide range of proteins that label specific subcellular and cellular components, and macromolecular complexes. Antibodies were made to synaptobrevin (SNB-1), a component of synaptic vesicles; to Rim (UNC-10), a protein localized to synaptic active zones; to transforming acidic coiled-coil protein (TAC-1), a component of centrosomes; to CENP-C (HCP-4), which in worms labels the entire length of their holocentric chromosomes; to ORC2 (ORC-2), a subunit of the DNA origin replication complex; to the nucleolar phosphoprotein NOPP140 (DAO-5); to the nuclear envelope protein lamin (LMN-1); to EHD1 (RME-1) a marker for recycling endosomes; to caveolin (CAV-1), a marker for caveolae; to the cytochrome P450 (CYP-33E1), a resident of the endoplasmic reticulum; to β-1,3-glucuronyltransferase (SQV-8) that labels the Golgi; to a chaperonin (HSP-60) targeted to mitochondria; to LAMP (LMP-1), a resident protein of lysosomes; to the alpha subunit of the 20S subcomplex (PAS-7) of the 26S proteasome; to dynamin (DYN-1) and to the α-subunit of the adaptor complex 2 (APA-2) as markers for sites of clathrin-mediated endocytosis; to the MAGUK, protein disks large (DLG-1) and cadherin (HMR-1), both of which label adherens junctions; to a cytoskeletal linker of the ezrin-radixin-moesin family (ERM-1), which localized to apical membranes; to an ERBIN family protein (LET-413) which localizes to the basolateral membrane of epithelial cells and to an adhesion molecule (SAX-7) which localizes to the plasma membrane at cell-cell contacts. In addition to working in whole mount immunocytochemistry, most of these antibodies work on western blots and thus should be of use for biochemical fractionation studies.

**Conclusions/Significance:**

We have produced a set of monoclonal antibodies to subcellular components of the nematode *C. elegans* for the research community. These reagents are being made available through the Developmental Studies Hybridoma Bank (DSHB).

## Introduction

Antibodies are critical tools for the study of cell and developmental biology in metazoans. They are widely used for the detection and characterization of cellular components *in situ*, in cell culture, and in the test tube. Although other means to detect and study proteins *in vivo* have been developed over the last decade including the extensive use of green fluorescent protein (GFP) and GFP-derivatives, antibodies remain exceedingly valuable reagents for modern biology research.

Both monoclonal and polyclonal antibodies can be made using different methodologies. Polyclonal sera have the advantage of typically being of higher affinity, but they are non-renewable resources. Monoclonal antibodies expressed by antibody expressing hybridomas are generally of more modest affinity, but higher specificity since each monoclonal recognizes only a single epitope. The greatest value of monoclonal reagents is that they can be produced in limitless quantities. Another is that they can be used in conjunction with polyclonal antibodies raised in other species for double labeling.

Though monoclonal and polyclonal antibodies can be produced in the research laboratory setting, most research labs working with vertebrates avail themselves of the wide array of both monoclonal and polyclonal antibodies available for purchase from numerous companies. By contrast, few such commercial reagents are available for work with model organisms such as *C. elegans*. Since most *C. elegans* proteins are significantly divergent from their vertebrate orthologs few commercial reagents are useful for the study of *C. elegans*. Furthermore, only a very modest number of immunological reagents have been made to *C. elegans* cellular components. Most of these antibody reagents are polyclonals made by individual investigators. As a result, many are not widely available (either because the sera is not being distributed or has all been used) or are only available in limited quantities.

Previously a number of monoclonals have been developed against specific *C. elegans* components. A few individual scientists have expended the effort to develop monoclonals against specific reagents of use in their research (e.g. α-PAR-3 and α-CHA-1; [1], [2]). Another approach by researchers in an attempt to obtain monoclonal antibodies to one organism has been to use the entire organism or cellular parts (*e.g.* head, ovary extracts, muscle) of the organism as an immunogen. This approach has been taken for the organism, *Ascaris suum* [3], *Macrostomum* sp. [4], *Aspergillus* [5], *Drosophila* [6], and *C. elegans* [7], [8], [9], [10], [11]. A difficulty with this approach is the enormous amount of time and expense it takes to characterize the group of monoclonal antibodies, since the antigen recognized by the monoclonal is unknown without further characterization.

In this study, we describe the creation and characterization of mouse monoclonal antibodies against a variety of nematode proteins with the aim of providing a set of tools that can be used by the scientific community to label specific cellular and subcellular compartments in *C. elegans*. The targets chosen include common organelles (*e.g.* endoplasmic reticulum, endosomes), specialized organelles (*e.g.* synaptic vesicles), subcellular domains common to all cells (*e.g.* centrosomes, nuclear membrane), and macromolecular complexes (*e.g.* proteasomes, replication origin complex). These reagents are being made available to the scientific community through the Developmental Studies Hybridoma Bank (DSHB), which provides monoclonal reagents in the form of cell lines and cell products at modest cost.

## Results

To develop monoclonal reagents that detect distinct subcellular compartments and structures for *C. elegans* we opted primarily to create monoclonal antibody lines against protein that had previously been documented to be detectable at endogenous levels in wild type *C. elegans* using a polyclonal sera. Below we describe each monoclonal reagent that we created that performed adequately in western blot and/or whole mount immunocytochemistry on wild type animals. Table 1 provides a summary of these antibodies. A list of other antigens to which we developed monoclonal antibodies, which failed in our tests to detect endogenous protein is provided in the supplementary methods.

**Table 1:** Monoclonal antibodies described in this study.

Gene	mAb name(s)	Subcellular localization	Westerns	Immuno-histochemistry
SNB-1	SB1	synaptic vesicles	+++	+++
UNC-10	RIM RIM2	synaptic active zones	−	+++^1^
DLG-1	DLG1	adherens junctions	+++	+++
HMR-1	HMR1	adherens junctions	−	+++
ERM-1	ERM1	Apical membranes	+++	+++
LET-413	LET413	basolateral membranes	+++	+++
SAX-7	SAX7	plasma membrane at cell-cell contacts	+++ 2	+++
HCP-4	HCP4	centromeres	+	+++
LMN-1	LMN1	nuclear membranes	+++	+++
DAO-5	DAO5	nucleosomes	++^3^	+++
ORC-2	ORC2	replication origins	++	+++
PAS-7	PAS7	26S proteasome	+++	++
TAC-1	TAC1	Centrosomes	+++	+++
HSP-60	HSP60	mitochondria	+++	+++
CAV-1	CAV1	caveolae^4^	+++	+++
CYP-33a	CYP33a	ER	+	+++
SQV-8	SQV8	Golgi	+++	+++
LMP-1	LMP1	lysosomes	+++	+++^5^
RME-1	RME1	recycling endosomes	+++	+++
DYN-1	DYN1	sites of clathrin-mediated endocytosis	+++	+++
APA-2	APA2	clathrin adaptor	+	++
-------	his6	his_6_ tag	+++	+++

+++ = easily detected ++ = detected + = detectable − = not detected.

footnotes. See Text for additional details.

1 RIM, but not RIM2 also detects staining of the pharynx that does not disappear in *unc-10* mutants.

2 SAX7 monoclonal detects only a 60 kD proteolytic fragment.

3 multiple forms seen on Westerns which may represent multimers.

4 It is unclear if *C. elegans* has caveolae as traditionally defined.

5 some nervous system staining which remains present in *lmp-1* null mutants.

### SNB-1

**Figure 1:**
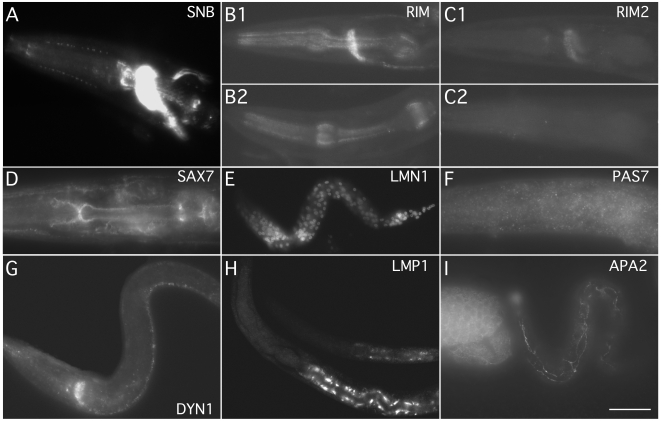
Whole mount immunocytochemistry of *C. elegans* larvae using monoclonal antibodies. A) The head of an adult animal shows the synaptic rich regions of the nervous system detected by the anti-synaptobrevin SB1 antibody. B1) Head of an L4 animal showing punctate synaptic staining of the nervous system detected by the RIM antibody. Note that some pharyngeal staining is also observed. B2) Head of an L4 animal showing loss of the punctate nervous system staining, but not the pharyngeal staining, in *unc-10(md1117)*. C1) Head of an L4 animal showing punctate synaptic staining in the nervous system detected by the RIM2 antibody. C2) Head of an L4 animal showing loss of the punctate nervous system staining in *unc-10(md1117)*. D) Head of an adult animal showing anti-SAX-7 staining of the neurons in the nerve ring ganglia and pharynx. E) An L1 animal showing staining of the nervous system with the anti-LMN-1 antibody. F) L4 animal showing punctate cytosolic anti-PAS-7 staining. G) An L2/L3 animal showing staining of the nervous system with the anti DYN-1 antibody. H) L1 (top) and L2 animals showing the staining of the intestine with the anti-LMP-1 antibody. I) an egg and L1 animal showing the APA-2 staining. APA-2 is particularly strong in the seam cells in L1. Scale bar = 20 µm.

**Figure 2:**
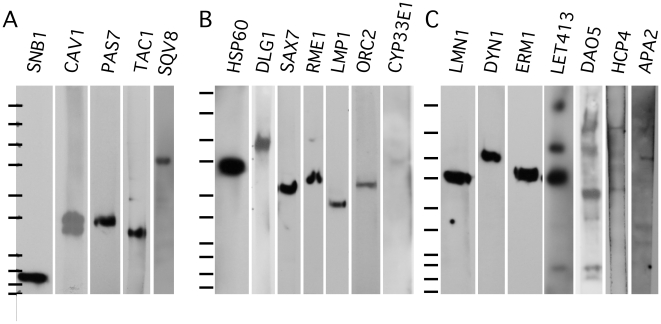
Western analysis using monoclonal antibodies. Western blots of whole wild type *C. elegans* extracts divided into three panels based upon acrylamide concentration used for SDS-polyacrylamide gel electrophoresis. The antibody used is labeled above each blot fragment. Approximately 20 to 30 µg of extract mixed staged extract was loaded in each lane. Molecular weight markers (BioRad Precision Plus Protein All Blue Standards) from top to bottom 250,150, 100, 75, 50, 37, 25, 20, 15, 10 kDa. the contrast was lowered on the images of the CAV1 and DAO5 westerns to permit visualization of multiple bands in a strong exposure.

*snb-1* encodes a neuronal v-SNARE synaptobrevin (VAMP) which is localized primarily to synaptic vesicles in *C. elegans* [12]. Monoclonal antibodies against *C. elegans* SNB-1, without the transmembrane region were made by immunizing mice with a His_6_-tagged fusion protein to amino acids 1-86 which had previously been used for the generation of rabbit polyclonal antibodies [12]. Three stable hybridoma cell lines (2E6, 5D1, and 5G1) were produced and all were of the isotype IgG1. All three of the monoclonal antibodies were examined on whole mount animals and found to have an immunostaining pattern similar to what had previously been reported with a polyclonal antibody [12]; bright signals in the nerve ring, dorsal and ventral nerve cords (Figure 1A). Western blots of wild type and mutant animals [12], *snb-1(md247)*, showed that the monoclonal antibody was specific Figure 2A. A 16 kDa band was seen in wild type lysate, and in *snb-1*(*md247*) lysates this band shifted to 19 kDa and was reduced in intensity (data not shown) as has previously been reported [13]. Of the three hybridoma cell lines, 5D1 was re-named SB1 and was sent to the Developmental Studies Hybridoma Bank (DHSB). This SB1 monoclonal antibody has been previously used on westerns to detect SNB-1 protein that had been immunoprecipitated by the polyclonal antibody from soluble extracts of *C. elegans* [14]. Finally, the epitope of this monoclonal (PRPSNKRLQQ) had been deduced by others [15].

### UNC-10

*unc-10* encodes a homolog of the vertebrate proteins RIM1 and RIM2 which is specifically localized to the periphery of the active zone in neurons in *C. elegans* [16], [17]. The *C. elegans* UNC-10 monoclonal antibodies were made by immunizing mice with a His_6_-tagged fusion protein to the N-terminus zinc finger domain, amino acids 1–144 which had previously been used for the generation of rabbit polyclonal antibodies [17]. Seven stable hybridoma cell lines (1D8, 1H4, 2E11, 2F3, 3H9, 4A5, and 4E11) were produced of the isotype IgG1, except for 2F3, which was of the isotype IgG2a. All seven monoclonal antibodies immunostained whole mount animals with a pattern that had previously been reported with the polyclonal antibody [17]: it detects a dense network of puncta in the nerve ring, dorsal and ventral nerve cords (Figure 1B1 & C1 and data not shown). Immunostaining was absent in *unc-10(md1117)* with the monoclonal antibodies 2E11 and 4E11 (Figure 1C1 and C2 and data not shown). A similar pattern of immunostaining was seen with the monoclonal antibody, 4A5, except some faint staining was seen in the pharynx in the mutant strain (Figure 1B2). Western blots of extracts of transgenic animals carrying the N-terminal domain of UNC-10 documented that the antibody will detect the zinc finger domain on westerns [14], but the antibody has not been reliable in detecting native UNC-10 perhaps due to difficulties in transferring the protein to membranes (data not shown). The 4A5 and 4E11 hybridoma cell lines, re-named RIM and RIM2, have been submitted to DHSB in Iowa for distribution to the scientific community.

### DLG-1

**Figure 3:**
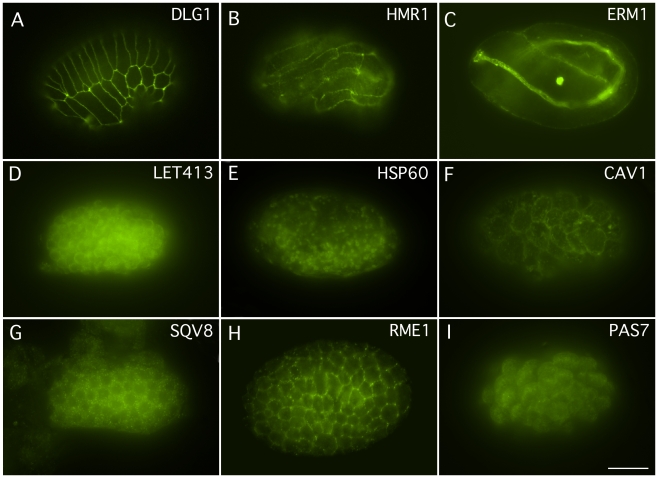
Whole mount immunocytochemistry of embryos using monoclonal antibodies. A) Pre-comma staged embryo showing anti-DLG-1 staining in developing hypodermal cells. B) A three-fold embryo showing anti-HMR-1 staining of adherens junctions. C) A three-fold embryo showing anti-ERM-1 staining in the apical junctions in the developing pharynx and intestine. D) A pre-comma embryo showing anti-LET-413 staining. E) A one and half-staged embryo showing anti-HSP-60 staining. F) A ∼40 cell embryo showing anti-CAV-1 staining. G) A pre-comma staged animal showing anti-SQV-8 staining. H) A pre-comma staged embryo showing anti-RME-1 staining. I) An ∼60 staged embryo showing anti-PAS-7 staining. Scale Bar = 10 µm.

*dlg-1* encodes a *C. elegans* homolog of the membrane-associated guanylate kinase (MAGUK) family of scaffolding proteins that includes drosophila *disks large* protein, after which *dlg-1* is named. In *C. elegans*, DLG-1 is primarily associated with the adherens junctions [18], [19]. The monoclonal antibody against *C. elegans* DLG-1 was made by immunizing mice with a His_6_-tagged fusion protein encompassing amino acids 204–593 (PDZ domain 1–3). This construct contained the same DLG-1 coding fragment that had previously been reported for the generation of rabbit and rat polyclonal antibodies [19]. One stable hybridoma cell line, 5E2, was produced and isotyped as IgG1. On whole mount immunostain of wild type *C. elegans*, an immunostain pattern was seen that had previously been reported with the polyclonal antibody and DLG-1-GFP transgenes [18], [19], [20], [21]: adherens junctions of hypodermis, gut, and pharynx (Figure 3A and data not shown). Analysis of the monoclonal on western blots of wild type animal lysates showed that the 5E2 monoclonal antibody recognized an 107 kDa protein band (Figure 2B). 5E2, re-named DLG1, has been submitted to the DHSB.

### HMR-1

*hmr-1* encodes a member of the classical cadherin family of cell adhesion molecules. *hmr-1* is expressed in many tissues and timed during development; among other places it is highly expressed in apical junctions of hypodermal cells during embryonic development and later additionally becomes localized to the apical margins of the intestine and pharynx [22], [23], [24]. The monoclonal antibody against *C. elegans* HMR-1 was made by immunizing mice with a His_6_-tagged fusion protein encompassing amino acids: 1099–1223. Previously, a polyclonal antibody had been reported in the literature to be made from fourteen amino acids (HMR-1a isotype amino acids: 1196–1209 of the C-terminus of HMR-1 [23]. Two stable hybridoma cell lines, 8H10 and 1A7, were produced. The 8H10 monoclonal was the isotype IgM and the 1A7 monoclonal was a mixed isotype of IgG1+IgG2a+IgM. On whole mount immunostain of wild type animals, an immunostain pattern was seen that had previously been reported in the literature for the polyclonal antibody and the GFP-fusion construct [22], [23], [24]: nervous system, adherens junctions, blastomeres of embryos, regions of contact between blastomeres, apical margins of all hypodermal, intestinal, and pharyngeal cells (Figure 3B and data not shown). With the 8H10 monoclonal antibody, no protein bands could be detected on western blots (data not shown). With the 1A7 monoclonal antibody a protein band of 130 kDa was observed which is in agreement with the expected size of the HMR-1 protein. Despite the potential benefits of the 1A7 line, the 8H10 monoclonal antibody, re-named HMR1, has been submitted to the DHSB because it is of a single isotype.

### ERM-1

*erm-1* encodes a homolog of the ezrin-radixin-moesin (ERM) family of cytoskeletal linker proteins. It localizes most prominently to the apical membranes in the polarized alimentary tract, but is also found on the apical cell cortex of many cells during development [25]. Monoclonal antibodies against ERM-1 were made by immunizing mice with a His_6_-tagged fusion protein encompassing amino acids 209–563 of ERM-1. This fusion protein is similar to the fusion protein, His_6_-ERM-1, amino acids 207–563, that was used to make a previously reported polyclonal antibody [25]. Four stable hybridoma cell lines (2H11, 5E2, 5G2, and 7G2) were produced and were isotyped as IgG1. The ELISA assays indicated that the monoclonal antibodies, 7G2 and 5G2, were specific to the ERM-1 fusion protein and not to the His_6_-tag. Whereas, the monoclonal antibodies, 2H11 and 5E2, reacted to the His_6_-tag. On whole mount immunostaining of wild type, an immunostain pattern was observed with the 7G2 and 5G2 monoclonal antibodies that resembled the pattern that had previously been reported for an ERM-1 polyclonal antibody and the GFP-ERM-1 reporters [25], [26]: cell cortex of most cells, cell cortex of contacting membranes in early stages, the apical cortex of the pharyngeal-intestinal tract, hypodermal cells (Figure 3C and data not shown). Immunoblots of *C. elegans* wild type lysates were probed with the monoclonal antibodies. Both the 5G2 and 7G2 monoclonal antibodies recognized a single protein band of 72 kDa on western blots (Figure 2C and data not shown), in good agreement with the 65 kDa protein detected with polyclonal sera [25]. Since the 7G2 antibody occasionally also detected a 10 kDa band on westerns, the 5G2 monoclonal antibody, re-named ERM1, was submitted to the DHSB.

### LET-413

*let-413* encodes a protein homolog of ERBIN, DENSIN-180 and SCRIB, an epithelial basolateral membrane protein, in the LAP family of proteins (contains leucine repeats and PDZ domains) [27]. Monoclonal antibodies were made against amino acids, 460–606. This is similar to the domain that was used in making the polyclonal antibody, amino acids 469–576 [28]. A polyclonal antibody was also made to a 19 amino acid peptide, amino acids 592–606 [27]. Two stable hybridoma cell lines, 4E3 and 5C12, were obtained and all were the isotype IgM. On whole mount immunostains of wild type, a staining pattern was observed that was similar to what had been reported for the polyclonal antibodies and the GFP-construct [27], [28], [29]: all cells of early embryos, epidermal cells, spermatheca, pharynx, intestine, uterus, and nerve ring (Figure 3D and data not shown). On western blots of wild type lysates, the 5C12 monoclonal antibody detected protein bands of 76 kDa, as well as fainter bands of 110 kDa and 200 kDa (Figure 2C). This size is in good agreement with the predicted Mw of the LET-413 protein, 75 kDa, which was recognized by the polyclonal antibodies [27]. The 5C12 monoclonal antibody, re-named LET413, has been submitted to the DHSB.

### SAX-7

*sax-7* encodes a homolog of the cell adhesion molecule L1/CAM which localizes most prominently to sites of cell-cell contact in the nervous system. Monoclonal antibodies were made against a His_6_-tagged fusion to the SAX-7 cytoplasmic domain, amino acids 1051–1144. Four stable hybridoma cell lines (5.2.1, 5.2.6, 11.2.1, and 11.2.3) were produced. Two of these monoclonals, 5.2.1 and 5.2.6, were isotyped as IgG2b and the other two monoclonals, 11.2.1 and 11.2.3, were isotyped as IgG1. On whole mount immunostain of wild type *C. elegans*, an immunostain pattern was observed that had previously been reported with the polyclonal antibody which had been raised to a peptide on the C-terminus cytoplasmic domain of SAX-7 [30], [31], [32]: plasma membrane at sites of cell-cell contact throughout development, nervous system, pharynx, hypodermis, body wall muscles, and germline (Figure 1D and data not shown). Surprisingly, western blots of wild type lysates showed that the monoclonal antibodies only recognized a 55 kDa protein band (Figure 2B). Immunoblots of *C. elegans* that had been probed with the C-terminus SAX-7 peptide antibody detected bands of 200 kDa, 185 kDa, 120 kDa, and 65 kDa [30], [31]. Another group that used the same polyclonal antibody had detected protein bands of 160 kDa, 140 kDa, and 45 kDa on westerns [33]. All three research groups proposed that the 45–65 kDa protein band was the result of post-translational cleavage of the long and short forms of the SAX-7 protein. The relative abundance of these isoforms is unclear, though the 65 kD band was by far the brightest signal in the only westerns that were shown in full [30]. The larger forms appeared to be phosphorylated [30]. Thus, by western analysis, it appears that these monoclonal antibodies only recognize the post-translational cleaved product and not the short or long version of SAX-7. This could be because the antibody recognizes the unphosphorylated version of an epitope that is phosphorylated *in vivo*. The monoclonal line 11.2.1, re-named SAX7, has been submitted to the DHSB.

### HCP-4

**Figure 4:**
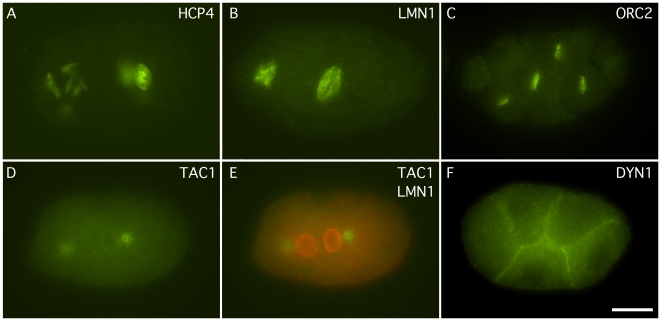
Whole mount immunocytochemistry of 2, 4 and 6 cell embryos using monoclonal antibodies. A) A two cell embryo showing anti HCP-4 staining of chromosomes. B) A two cell embryo showing anti-LMN-1 staining of the nuclear envelope. C) A six cell embryo showing ORC-2 staining of chromosomes. D) A two cell embryo showing staining of anti-TAC-1 of centrosomes. E) Double labeling using the anti-TAC-1 monoclonal and an anti-LMN-1 polyclonal antibody. F) A six cell embryo showing anti-DYN-1 staining. Scale Bar 10 µm.

*hcp-4* encodes a homolog of CENP-C which localizes to the centromere of chromosomes [34], [35]. Due to the holocentric nature of *C. elegans* chromosomes, their centromeres are more extended, diffuse and discontinuous than typical centromeres [36], [37]. The *C. elegans* HCP-4 monoclonal antibodies were made by immunizing mice with a His_6_-tagged fusion protein consisting of amino acids 9–210. Three stable hybridoma cell lines (2E2, 3F4, 4B7) were obtained and all were of the isotype IgM, except 4B7, which appeared to be a mix of the isotypes, IgM, IgG1, and IgG2a. The two monoclonals, 2E2 and 3F4, were used to examine the immunostain pattern on whole mount embryos and were found to stain in a pattern that was similar to what had been previously reported for the polyclonal antibody [34], [35]: nuclei and chromosomes (Figure 4A). The monoclonal antibodies against HCP-4 were examined on western blots of wild type lysates of *C. elegans* and found to recognize faint protein bands of 125 kDa and 80 kDa (Figure 2C). Two protein bands, of 136 kDa and 70 kDa, had previously been detected on westerns of wild type embryo extracts using an HCP-4 polyclonal antibody, and the 70 kDa band was determined to be independent of the primary antibody [35]. 2E2 was re-named HCP4 and has been submitted to the DHSB.

### LMN-1

*lmn-1* encodes a *C. elegans* homolog of nuclear lamin, a structural nuclear envelope protein of the intermediate filament super family that associates with the nuclear membrane [38]. A rabbit anti-LMN-1 polyclonal antibody directed against the rod and tail region of *C. elegans* LMN-1 (amino acids 217–550), which recognized endogenous LMN-1 has previously been reported [38], [39]. Monoclonal antibodies were made against a full length *C. elegans* His_6_-tagged LMN-1 fusion protein by immunizing mice. Four stable hybridoma cell lines (1B6, 2A7, 2G1, and 3D7) were produced and all were isotyped as IgG1. These monoclonals were used to immunostain wild type whole mount animals and were found to label in a pattern that had been previously reported for the polyclonal antibody [38], [39], [40]: nuclear periphery (inner) and nuclear interior of most cells (Figure 1E and 4B). Western blot analysis of wild type lysates probed with these monoclonal antibodies revealed that they recognized a single protein band of 73 kDa (Figure 2C), in reasonable agreement with the 64–66 kDa detected on westerns by polyclonal sera [38], [39]. The 2G1 monoclonal antibody, re-named LMN1, has been submitted to the DHSB.

### DAO-5

**Figure 5:**
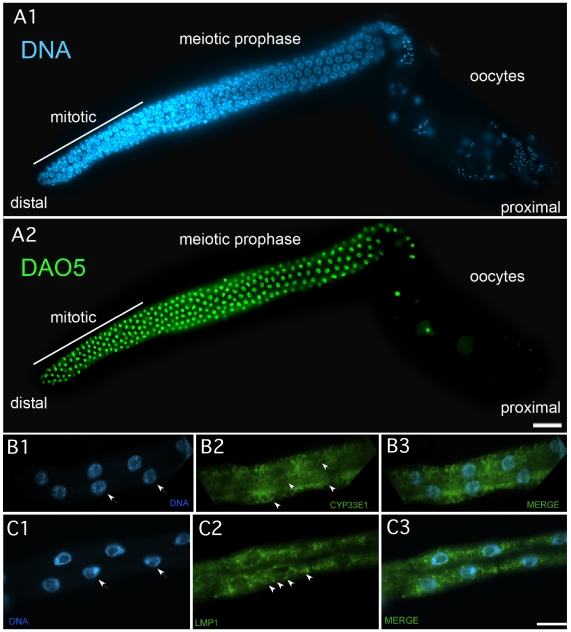
Immunocytochemistry of dissected gonad and intestines using monoclonal antibodies. A) Dissected gonad stained with DAPI (A1) to visualize nuclei and immunostained with anti-DAO-5 antibodies (A2). B) Dissected intestine stained with DAPI (B1) to visualize nuclei (arrows) and immunostained with anti CYP-33E1 antibodies (B2) and the merged view (B3). C) Dissected intestine stained with DAPI (C1) to visualize nuclei (arrows) and immunostained with anti LMP-1 antibodies (C2) and the merged view (C3).

*dao-5* encodes a homolog of the nucleolar phosphoprotein Nopp140 [41]. Monoclonal antibodies against *C. elegans* DAO-5 were made by immunizing mice with a His_6_-tagged fusion protein to amino acids 751–971. Six stable hybridoma cell lines (5E9, 7G8, 11A8, 13D5, 14C2, and 13H10) were produced and were the isotype IgG1, except for 5E9 which was the isotype IgG2b, and 14C2 which was a mix of the isotypes IgG1 and IgG2b. In whole mount immunocytochemistry, the single isotype monoclonals all stained the nucleolus and nucleoplasm of somatic cells and germ cells (Figure 5A1 and A2), which was similar to what was seen with another nucleolus protein, nucleostemin [42]. Western blots of wild type animal lysates revealed that the monoclonal antibodies recognized multiple protein bands of 150 kDa, a dimer, and 60 kDa. With longer exposures, protein bands of 100 kDa, a dimer, 36 kD, 20 kDa and 10 kDa were observed (Figure 2C). The western analysis is consistent with prior reports that multiple forms of this protein are often detected on westerns due to post-translational modification, proteolysis, dimerization, trimerization and alternative splicing [43], [44], [45], [46], [47]. The monoclonal antibody 5E9, re-named DAO5, has been submitted to the DHSB.

### ORC-2

*orc-2* encodes an evolutionarily conserved subunit of the origin replication complex [48]. The monoclonal antibody against *C. elegans* ORC-2 was made by immunizing mice with a full length His_6_-tagged fusion protein, which was purified under denaturing conditions. Three stable hybridoma cell lines (1H7, 4G8, and 4D4) were produced and all were isotyped as IgG1. All three of the monoclonal antibodies were examined on whole mount animals and found to immunostain the nucleus and what appears to be the gonadal primordium (Figure 4C and data not shown). Previously, it has been reported that the Drosophila and human ORC2 proteins localize predominantly to the nucleus [49], [50]. All three monoclonal antibodies recognized a protein band of 49 kDa on western blots, the expected size of the ORC-2 protein (Figure 2B). 4D4, renamed ORC2, has been submitted to the DHSB.

### PAS-7

*pas-7* encodes the *C. elegans* ortholog of an evolutionarily conserved subunit of the 26S proteasome subunit which is involved in regulated protein degradation [51]. Monoclonal antibodies were made against a purified His_6_-tagged PAS-7 full length fusion protein. Six stable hybridoma cell lines (1A1, 2H4, 5B3, 5H2, 6C1, and 6C6) were produced and all were found to be the isotype IgG1. All six monoclonal antibodies were examined on lysates of *C. elegans* whole mounts and were found to label the nucleus as well as the cytoplasm (Figure 1F and 3I) which was similar to what has been reported for ataxin, ATX-3, which was proposed to escort ubiquitylated substrates for proteasome degradation, was found to be expressed in all developmental stages and was found in the nucleus and cytoplasm [52], [53]. The 6C1 and 5B3 antibodies were found to label multiple protein bands on western blots of wild type extracts, while 1A1, 2H4, 5H2 and 6C6 were found to specifically label a single protein band of 28 kDa, consistent with the predicted size of PAS-7 (Figure 2A and data not shown). The monoclonal antibody 1A1, re-named PAS7, has been submitted to the DHSB.

### TAC-1

*tac-1* encodes a protein homologous to the transforming acid coiled coil protein family which localizes to centrosomes in a cell cycle dependent manner [54]. A polyclonal antibody directed against full length TAC-1 recognized the endogenous protein in early embryos [54]. A His_6_-tagged TAC-1 full length fusion protein was used to immunize mice. Four stable hybridoma cell lines (4.5.4, 4.5.6, 6.2.1, and 6.4.2) were produced, and all were isotyped as IgG1. On whole mount immunostain of *C. elegans* embryos, a pattern was seen which was similar to that reported for both a polyclonal antibody and TAC-1::GFP fusions [54], [55]: staining at meiotic spindle poles, at centrosomes in one-cell embryos and in the cytoplasm (Figure 4D and E). Western blots of wild type extracts showed that the monoclonal antibodies recognized primarily a 35 kDa protein band (Figure 2A). The 4.5.6 monoclonal antibody, re-named TAC1, was submitted to the DHSB.

### HSP-60

*hsp-60* encodes a mitochondrial targeted heat shock protein. Monoclonal antibodies were made against *C. elegans* His_6_-tagged HSP-60a fusion protein, amino acids 1–547. Four stable hybridoma cell lines (1G3, 1H12, 2H6, 2H12) were obtained. All were isotyped as IgG1, except for 1G3 that was isotyped as IgG2b. On whole mount immunostain of wild type *C. elegans*, an immunostain pattern was observed similar to that previously reported for mitochondrial staining [56], [57]: signal around most nuclei, speckles in mitotic germ cells, foci in the cytoplasm of embryonic cells, and elongated tubular morphology in body wall muscle (Figure 3E). Immunoblots of *C. elegans* wild type lysates revealed that the monoclonals recognized a 65 kDa band in agreement with the 60 kD size of the HSP-60a form of the protein (Figure 2B). The monoclonal antibody, 1H12, re-named HSP60, has been submitted to DHSB.

### CAV-1

*cav-1* encodes one of two caveolin homologs in *C. elegans*. Vertebrate caveolins are the major proteins required for the formation of plasma membrane invaginations called caveolae [58]. It remains unclear whether *C. elegans* has caveolae or if *C. elegans cav* proteins localizes to them. However, both CAV-1 and CAV-2 localize to both the plasma membrane and to intracellular structures referred to as CAV bodies [59], [60]. Monoclonal antibodies against *C. elegans* CAV-1 were made by immunizing mice with a full-length His_6_-tagged fusion protein. A polyclonal antibody raised against a C-terminal peptide of *C. elegans* CAV-1, amino acids 219–232, has been previously reported to detect endogenous CAV-1 [61]. Six stable hybridoma cell lines (1A3, 1A7, 1B4, 1F6, 1F12, and 3F1) were produced and were of the isotype IgG1, except for 1B4 which was the isotype IgG3. All seven monoclonal antibodies immunostained whole mount animals with a pattern that had previously been reported with the polyclonal antibody and GFP-fusion constructs [59], [61], [62]: gonad germ cells, most embryonic cells, neurons, ventral nerve cord, commissures and body wall muscles (Figure 3F and data not shown). Western blot analysis of wild type lysates revealed that the 1F6 monoclonal antibody recognized two protein bands of 29 kDa and 32 kDa (Figure 2A). The polyclonal antibody to the C-terminus peptide of CAV-1 was reported to recognize a single band of 31 kDa [61]. SDS-PAGE that had been done on the full-length protein which had been expressed in COS-7 cells, revealed a protein of 32–33 kDa [63]. The mammalian caveolin protein has been found to exist in both a and b forms, which differ by 3 kD in size [64], analogous to what we observe with the IF6 monoclonal antibody. 1F6, re-named CAV1, has been submitted to the DHSB.

### CYP-33E1

*cyp-33E1* encodes a cytochrome P450 family member involved in the metabolism of isoniazid and likely localized to the endoplasmic reticulum similarly to most P450 family members [65]. A full length His_6_-tagged fusion protein of *C. elegans* CYP-33E1 was used to immunize mice in the production of monoclonal antibodies to CYP-33E1. Two stable hybridoma cell lines, 1D6 and 1G3, were produced and were isotyped as IgM. On whole mount immunostain of wild type *C. elegans* (Figure 5B), a similar pattern was seen that had previously been reported for rough endoplasmic reticulum membrane proteins and endoplasmic reticulum proteins [66]. Analysis of these monoclonals on western blots of wild type animal lysates showed that they labeled a protein band of 73 kDa (Figure 2B). Two faint band of 90 kDa and 110 kDa could also be detected. The predicted size of CYP-33E1 is 58 kDa. The monoclonal antibody, 1G3, re-named CYP33E1 has been submitted to DHSB.

### LMP-1

*lmp-1* encodes a homolog of the lysosome associated membrane protein family (LAMPs) and is highly expressed and localized to lysosomal-like granules in the *C. elegans* intestine which can be detected in wild type animals using an anti-LMP-1 polyclonal sera [67]. The *C. elegans* monoclonal antibodies against LMP-1 were made by immunizing mice with a His_6_-fusion protein consisting of amino acids 18–160 of LMP-1. Two stable hybridoma cell lines, 2E2 and 2H11, were obtained and all were of the isotype IgG2b. Both monoclonals were used to examine the immunostain pattern on whole mount wild type and *lmp-1(nr2045)* animals [67]. In wild type animals, the monoclonals were found to stain in a pattern that was similar to what had been previously reported for the polyclonal antibody and the GFP-construct [67], [68]: subcellular granules distributed throughout the early embryo, intestinal granules from late embryo to adults, coelomocytes, lysosomes (Figure 1H, 5C). In mutant animals, the staining pattern that was seen in the wild type animals disappeared, but the immunostaining of the nervous system remained and became more pronounced (data not shown). This could be due to the monoclonal antibodies cross-reacting to another LAMP protein such as UNC-46, which has a conserved secondary structure with LMP-1 and is localized at synapses and expressed in GABA neurons in *C. elegans* [69]. Western blots of wild type and mutant animals, *nr2045*, showed that the two monoclonal antibodies against LMP-1 were specific. A 37 kDa protein band was detected in wild type *C. elegans* lysate but not in *nr2045* mutant lysates (Figure 2B and data not shown). The monoclonal antibody 2E2, re-named LMP1, has been submitted to the DHSB

### RME-1

*rme-1* encodes an evolutionarily conserved protein specifically associated with recycling endosomes [70]. An anti-RME-1 polyclonal serum detects RME-1 in a punctate pattern in multiple tissues including the germline and intestine [70]. *C. elegans* monoclonal antibodies against RME-1 were made by immunizing mice with a His_6_-RME-1 fusion protein consisting of amino acids 333–575 of the RME-1d isoform. Nine stable hybridoma cell lines (4E12,5G11,6B6,6D1,6F10, 7E2, 8D8, 10A5, and 10B10) were obtained, and all were isotyped as IgG1. Two of the monoclonal antibodies, 5G11 and 7E2, were examined on wild type whole mount animals and were found to stain in a similar pattern that had been reported for the polyclonal antibody and the GFP-constructs [70]: germline cytoplasm, intestine, pharynx, associated with the periphery of endocytic organelles (Figure 3H and data not shown; also see [71]). A similar pattern of staining was seen with the 4E12 and 6B6 monoclonal antibodies (Data not shown and B. Grant unpublished data). Western blots of wild type lysates showed that a protein band of 70 kDa was detected by the 5G11, 7E2, 6B6, and 4E12 monoclonals (Figure 2B and data not shown). The other monoclonals labeled only very weakly (data not shown). The monoclonal 5G11, re-named RME1, has been submitted to the DHSB.

### SQV-8

*sqv-8* encodes a Golgi enzyme glucuronyl transferase that is a homolog of three glucuronyl transferases, GlcAT-I, GlcAT-P, and GlcAT-D that are involved in the synthesis of different glycoconjugates [72], [73]. The *C. elegans* SQV-8 monoclonal antibodies were made by immunizing mice with a His_6_-tagged SQV-8 fusion protein encompassing amino acids 150–349. This is the same domain used to make SQV-8 polyclonal antibodies [59]. Two stable hybridoma cell lines, 5B3 and 3B7, were produced and isotyped IgG1 (3B7) and IgG2b (5B3). On whole mount immunostaining of wild type *C. elegans* (Figure 3G and data not shown), all of the antibodies exhibited similar staining patterns, labeling structures (probably Golgi) around the nucleus and also stain the vulva [59]. On western blots of wild type *C. elegans* lysates, a band of 75 kDa was recognized by the 5B3 monoclonal antibody (Figure 2A). The predicted mass of SQV-8 is 41 kDa. The 5B3 monoclonal antibody, re-named SQV8, has been submitted to the DHSB.

### DYN-1

*dyn-1* encodes a homolog of dynamin 1 which regulates clathrin mediated endocytosis. Dynamin is concentrated at sites of clathrin mediated endocytosis, where it binds to the neck of budding clathrin coated pits. A polyclonal antibody directed against the C-terminal half of the *C. elegans* DYN-1 protein detects the endogenous protein in the nervous system [74]. Monoclonal antibodies against *C. elegans* DYN-1 were made by immunizing mice with a full length His_6_-tagged DYN-1 fusion protein (generously provided by Z. Zhou and N. Lu; Baylor College of Medicine). Five stable hybridoma cell lines (1A2, 2D6, 3H1, 5B1, and 5C2) were produced and found to be the isotype, IgG2a, except 5C2 which was the isotype IgG1. On whole mount immunostain of wild type *C. elegans*, an immunostain pattern was seen that had previously been reported with the polyclonal antibody and GFP-fusion constructs [74], [75], [76], [77], [78]: nerve ring, ventral and dorsal nerve cord, pharyngeal neurons, pharyngeal-intestinal valve, intestinal-rectal valve, intestinal cell apical surface, gonadal sheath, spermatheca, coelomocytes, cleavage furrow membranes (newly formed) and the midbody of dividing embryos (Figure 1G and 4F and data not shown). Analysis of the 1A2, 3H1, and 5B1 monoclonal antibodies on western blots of wild type animal lysates showed that they recognized a protein band of 100 kDa, in agreement with the predicted size of the protein (Figure 2C). 5B1, renamed DYN1, has been submitted to the DHSB.

### APA-2

*apa-2 (apt-4)* encodes an adaptin for the alpha subunit of the adaptor protein complex 2 (AP2) that is involved in endocytosis [79]. A polyclonal antibody has been raised against the C-terminal domain of *C. elegans* APA-2, amino acids 618–925 [80]. Six stable hybridoma cell lines (1C8, 7D1, 7F5, 9C3, 10D1, and 11B11) were produced and were of the isotype IgG1. All monoclonal antibodies, except 1C8, immunostained whole mount animals with a pattern that had previously been reported with the YFP-fusion constructs and polyclonal antibody [80], [81]: pharynx, intestine, body wall muscle, hypodermis, seam cells and motor neurons (Figure 1I and data not shown). Western blot analysis of wild type N2 lysates revealed that the 11B11 monoclonal antibody recognized protein bands of 102 kDa and 250 kDa (Figure 2C), which is in good agreement with the predicted size of the protein, 104 kDa. The monoclonal antibody, 11B11, re-named APA2, has been submitted to the DHSB.

## Discussion

We have isolated a modest set of monoclonal antibodies against a variety of *C. elegans* epitopes. The original goal of our project was to isolate 60 monoclonals to epitopes of *C. elegans*, but we succeeded in isolating only 21 monoclonals that met our criteria in detecting endogenous antigen on whole mount *in situs*, and/or western blots. We also isolated a monoclonal that recognizes a non-*C. elegans* epitope, the His_6_-tag, which was used to purify many of the fusion proteins. In addition, we isolated 20 monoclonals that did not meet our criteria in detecting the endogenous antigen on whole mounts and/or westerns. These 20 monoclonals were able to detect the recombinant antigen by ELISA and westerns, but do not: 1) detect the endogenous target protein on westerns and/or whole mount *in situs*, 2) immunostain expected patterns as was reported with the polyclonals and GFP-fusion constructs, and instead stain unexpected structures, 3) detect an endogenous protein band of an expected molecular weight on westerns and/or detect multiple bands. A list and brief description of these 20 additional monoclonals is provided in the supplemental materials section (Table S1; File S1). It should be noted that we did not analyze a large set of fixation conditions in testing these monoclonals. Thus, it may certainly be the case that some of these would recognize endogenous, or over-expressed targets in *C. elegans* using distinct fixation protocols or epitope recovery techniques. Likewise, the protocols for using the monoclonals that we have successfully used to detect antigen *in situ* may also not be optimal. In addition, we have not optimized the transfer conditions or transfer membranes for western analysis. Finally, we have not examined transgenic lines that over express antigens to test our panel of monoclonal lines.

Although we, or our colleagues [71], have performed experiments to demonstrate that some of the monoclonal antibodies are specific for the intended targets, we have not done so for all. There are several reasons for this decision. First, in many cases the appropriate tools are not available or have not been characterized. For example, null mutant alleles are unavailable or poorly characterized (e.g. knockout alleles which have not been confirmed or backcrossed). In other cases, mutants are lethal and balanced with markers that segregate aneuploids. In these cases significant experimental effort would have been required to develop or characterize the reagents needed to enable us to document the specificity of the monoclonal lines. Such effort would have come at the expense of our primary goal to create monoclonals for the community. Second, specificity is condition dependent. A monoclonal can be specific on whole mounts under one fixation condition, but not under another. Hence, since most researchers will be using these reagents in combination with other immunological reagents they have developed, it is likely that the conditions under which most of these tools will be used will differ from the high throughput methanol fixation protocol we used. Third, lack of specificity does not abrogate the potential use of a monoclonal. For example, the RIM1 antibody described in this paper is not specific for UNC-10 as it also recognizes signal in the pharynx, but it is still useful for the analysis of neuromuscular junctions in the ventral nerve cord. Thus, we opted to utilize our limited financial resources to maximize the number of monoclonal we could provide for the community, rather than to perform specificity experiments that for all intensive purposes would need to be repeated by scientists in their specific experimental systems.

The list of antigens that were chosen for this project was based on a collaborative effort among various *C. elegans* researchers. Input was solicited, and a list of 60 antigens for the production of monoclonals was assembled. Nine research laboratories further assisted us by their contribution of supplies towards our project in the form of cDNA clones, expression constructs, fusion protein, and/or polyclonal antibodies. All of these contributions, both in supplies and recommendations, were valuable resources.

The design for the antigenic epitopes that were used in making the monoclonal antibodies was based on the design of previously published *C. elegans* polyclonal sera. In cases where there were no previous polyclonals, the design of published mammalian polyclonals was taken into consideration in the design of the antigenic epitope. Finally, in cases where a polyclonal did not exist or the polyclonals reported in the literature were weak or not very specific in immunolabeling as compared to the analogous GFP-fusion constructs, we based our design of the epitope on the region that we thought would give us the best antigenic response. The logic for basing the epitopes on previous reported polyclonals were that since they had already been shown to be both antigenic and abundant, chances were that a successful monoclonal antibody could be obtained. However, most monoclonals that we produced were less effective at detecting targets in whole mount *in situs* than the polyclonal sera that was raised against the same antigen. Furthermore, many of the more robust antigens (as defined by the antigenicity and quality of polyclonal sera) did not yield useful monoclonal antibodies. One example of this was UNC-64, syntaxin, which was a very robust antigen but failed to yield a useful monoclonal in four fusion attempts. So, while the ability to raise polyclonal sera is likely a reasonable prerequisite to use in assessing whether to attempt to raise a monoclonal antibody, it is certainly not a direct indicator of the likelihood of success.

A variable in our project as it was undertaken, was the decision to inject with a His_6_-tagged fusion protein and to IV- or IP-challenge with a GST-tagged fusion protein. This was done only if the mouse polyclonal sera showed immunogenicity to both tagged fusion proteins on ELISAs. This approach did seem to increase our chances in obtaining successful monoclonal antibodies (Examples are: SAX-7, DLG-1, LMN-1, DAO-5, HSP-60, CYP-33E1, and SQV-8). However, there were cases where the mouse polyclonals only recognized one of the two fusion proteins, probably due to the intrinsic folding of the protein and exposure of the available antigenic epitope to the antibody. We believe that our chances in obtaining more successful monoclonal antibodies would have been increased if we had made even more fusion proteins to a variety of tags, both C- and N-terminus.

In conclusion, we present a set of monoclonal antibodies that we believe will be useful tools for the scientific community. These monoclonals can be used to label subcellular and cellular components, as well as macromolecular complexes. They will provide an unlimited source of antibody that cannot be exhausted as polyclonals can, and will enable the elimination of some artifacts that arise from immunolabeling using polyclonals that have been made from different animals. These monoclonals are being made available through the Developmental Studies Hybridoma Bank (DHSB).

## Materials and Methods

### Construction of expression plasmids

Fusion constructs were made from restriction enzyme digested purified PCR products that were amplified from *C. elegans* cDNA made from total RNA that was reverse transcribed using an oligo-dT or a random primer from a mixed-stage population of wild type N2 worms. The *unc-10* and *rab-3* fusion constructs were made from *C. elegans* cDNA clones obtained from Yuji Kohara. Details of all constructs made are available in File S2.

### Expression and Purification of Fusion Proteins

Fusion constructs were transformed into competent BL21 (DE3) cells (Invitrogen) and grown overnight at 37°C in Luria Broth (LB) supplemented with the appropriate antibiotic (100 µg/ml of ampicillin, 20 µg/ml of choramphenicol, or 30 µg/ml of kanamycin. The cells were diluted 100 times in fresh pre-warmed media, and grown to an OD_600_ = 0.6–0.7. Isopropyl β-D-thiogalactoside (IPTG) was added to 1 mM to induce expression of the fusion protein and the cultures was incubated overnight at room temperature.

His_6_ fusion proteins were batch purified on Nickel-nitrilotriacetic acid agarose (Qiagen, Valencia, CA) according to the manufacturer's protocols. The proteins were purified either in 8 M urea and renatured by dialysis against PBS (SNB-1, DLG-1, CAV-1, HCP-4, TAC-1, SAX-7, ERM-1, LMP-1, HMR-1, DAO-5, ORC-2, PAS-7, CYP-33E1, HSP-60, SQV-8, LET-413) or were purified under native conditions (UNC-10, SNB-1, APA-2, RME-1, and LMN-1) with modified buffers and dialyzed against PBS [82].

GST fusion proteins were purified with glutathione-agarose beads (Sigma-Aldrich, St. Louis, MO), under native conditions [83] and dialyzed into PBS [82]. The purity of the fractions was verified by sodium dodecyl-sulfate polyacrylamide gel electrophoresis (SDS-PAGE), and the concentration was determined by Bradford assays.

### Vertebrate Animals

Work with vertebrate animals (mice) in this study was performed under the guidance of the Office of Laboratory Animal Welfare (OLAW) in accordance with the Animal Welfare Act and complies fully with the Guide for the Care and use of Laboratory Animals (National Academic Press, 1996). Mice were housed and cared for by veterinarians in the Washington University School of Medicine Division of Comparative Medicine (DCM) Clinical Sciences Research Building animal facility. Immunization and blood drawing was performed by DCM staff. Mice were sacrificed to obtain spleen cells using C0_2_ inhalation, the recommend method of euthanasia by the Washington University School of Medicine Animal Studies Committee and a method approved by the American Veterinary Medical Association Guidelines on Euthanasia (June, 2007). The research involving the use of mice described in this manuscript was approved by the Washington University Animal Studies Committee (approval #20040341 and #20070289). Washington University's OLAW Animal Welfare Assurance Number is A3381-01 and Washington University is fully accredited by the AAALAC.

### Immunization

Three female inbred BALB/c mice (Taconics, Hudson, NY), 5–6 weeks of age, were immunized subcutaneously with a 1∶1 emulsified mixture of recombinant fusion protein in phosphate-buffered saline, pH 7.4 (PBS) at 50 µg per mouse using complete Freund's adjuvant (Sigma-Aldrich, St Louis, MO). Booster immunizations were done subcutaneously with a 1∶1 emulsified mixture of the fusion protein in PBS at 50 µg per mouse using incomplete Freund's adjuvant (Sigma-Aldrich). The Washington University Division of Comparative Medicine (DCM) animal facility housed the animals and administered all injections. To assess immunity and titer levels, both indirect ELISAs with the fusion protein as the coating antigen and immunofluorescence microscopy with fixed worms were performed. A final challenge injection of the fusion protein at 37.5 µg in PBS was done intravenously or intraperitoneally, 3–4 days prior to the fusion of the splenic lymphocytes with the myeloma cells. Detailed immunization schedules are provided in the supplemental methods.

### Cell Fusions

Mice were submitted to the Washington University School of Medicine Hybridoma Center (WUHC, St. Louis, MO http://pathology.wustl.edu/research/hybridoma.php), or to the Saint Louis University (SLU) Custom Hybridoma Development Service Facility (medschool.slu.edu/antibody/). WUHC performed all fusions except for TAC-1 and SAX-7, which were performed by the SLU Hybridoma Facility. The splenic lymphocytes were isolated and fused with P3X63Ag8.653 myeloma cells [84]. Indirect ELISA and whole mount *in situ* screening was performed to identify positives in the Nonet lab. Fusions that were performed by the SLU Hybridoma Facility were screened by their facility on immuno-dot blots with the fusion protein, prior to submitting the hybridoma supernatants to the Nonet lab for further screening. Cells that gave positive results were subcloned by limiting dilution by the hybridoma facility. The first subclone and final expanded supernatants were also screened on whole mount worms with immunofluorescence microscopy. The isotypes of the monoclonal antibodies were determined by ELISA with the following commercial anti-isotype reagents: goat anti-mouse IgG1(γ), IgG2a (γ), IgG2b (γ), IgG3 (γ), IgM (µ), IgA (α) (Caltag Labs, Burlingame, CA), rabbit anti-Mouse IgG, Kappa (USBiological, Swampscott, MA), goat anti-mouse IgG (1+2a+2b+3) Fcγ and goat anti-mouse IgG (H+L) (Jackson ImmunoResearch, West Grove, PA).

### Indirect ELISAs

Immulon 2HB 96-well flat bottom plates (VWR, Batavia, IL) were coated overnight at 4°C with 100 µl per well of the fusion protein in PBS at 1 ng/µl. The plates were washed with PBS and blocked with 2% bovine serum albumin (BSA) in PBS for 1–2 hours at RT. The plates were then washed twice with PBST (PBS plus 0.05% Tween-20), and the supernatants were dispensed into the wells at 50 µL/well and allowed to incubate for 1–2 hours at room temperature. Three washes with PBST were performed, followed by a 1-hour incubation with peroxidase-conjugated goat anti-mouse IgG (H+L) or (Fcγ) diluted in PBS. The plates were washed three times with PBST and the detection reagent, ABTS Peroxidase Substrate system (KPL), was applied. After incubation at RT for 15 minutes, the absorbance was measured at 405 nm, using a Perkin-Elmer plate reader.

To confirm specificity, all monoclonal antibodies were tested against two different tagged versions of the fusion protein (e.g. a His_6_-tagged protein and a GST tagged protein). In addition, they were also tested against tag-only controls (His_6_ or GST) in ELISA assays. The lone exception is the DYN-1 monoclonal antibody, which was only tested on one tagged fusion protein and the tag only control.

### Immunocytochemistry

*C. elegans* were grown at 22.5°C on nematode growth media (NGM) as described previously [85]. N2 wild type, *lmp-1(nr2045)* [67], *unc-10(md1117)* [17], and *snb-1(md247)* [12] strains were used in the study.

Two fixation conditions were used for the whole-mount immunohistochemistry: Bouin's [86] and methanol/acetone. For the methanol/acetone fix, tubes of frozen worms (washed off of large NGM plates) were thawed on ice and incubated in 2 volumes of cold methanol at 4°C for 10 minutes, sonicated on ice with a tip sonicator two times for 5 seconds each at maximal setting to break open the worms. The tube was incubated on ice for 10 minutes and spun down briefly in a microcentrifuge. The supernatant was aspirated and 2 volumes of −20°C acetone were added to the pellet. The worms were incubated on ice for 10 minutes and spun down briefly in a microcentrifuge. The supernatant was removed and 2 volumes of AbA (PBS + 0.05% Tween-20 + 0.5% BSA) were added to the pellet. The worms were then incubated with rolling agitation at RT for 30 minutes and spun down in a microcentrifuge. The supernatant was aspirated and two volumes of AbA + 20% glycerol was added to the pellet. The worms were then aliquoted and flash frozen in liquid N_2_ and stored at −80°C until needed.

For immunostaining, fixed worms were incubated in BLOCK [2% BSA + 2% gelatin+ 2% powdered milk (Carnation) +0.05% Tween-20 in PBS] for 1–2 hours. The worms were washed three times in PBST (PBS +0.1% Tween20), and incubated overnight at 4°C with a 1∶10 dilution of the hybridoma tissue culture supernatant in BLOCK. Worms were washed three times in PBST and incubated for 1 hour with the secondary antibody diluted in BLOCK. The secondary antibodies used to visualize the binding pattern of the primary antibodies were goat anti-mouse IgG Alexa Fluor® 488 or 568 (Invitrogen, Carlsbad, CA) at a 1∶2000 dilution. Gonads and intestines were fixed and stained as previously described [87].

Samples were examined on a Zeiss Akioskop equipped with an X-CITE 120 mercury bulb (EXFO) and standard epifluorescence filters. Images were captured using a Regita EXi cooled CCD camera and viewed using the Open Lab software (Improvision, PerkinElmer, Boston, MA). Image panels were assembled in Photoshop. In some cases, the corners of rotated images were colored to match the background. Additionally, other stained material in the field of view was excised to simplify images and replaced with background signal. Original images are available upon request. Gonad and Intestine images were assembled as previously described [87].

### Western Analysis

Western analysis was performed as described [13]. Mixed-staged animals were grown to near starvation and diluted 5 fold with an isotonic sucrose: HEPES solution (0.36 M sucrose, 12 mM HEPES, and protease inhibitors). The animals were sonicated for 10 seconds on ice, and then microcentrifuged for 15 minutes. Lysates typically contained about 3 mg/ml protein, and 20–30 µg was loaded onto a 15% SDS-PAGE gel and transferred to PVDF (Osmonics, Minnetonka, MN) using the a semi-dry transfer method using the manufacturer's protocol (BioRad, Hercules, CA). Hybridoma supernatants were diluted 1∶10 in PBS, and the secondary goat anti-mouse IgG (H+L) peroxidase conjugated antibody (Jackson ImmunoResearch) was used at a 1∶15,000 dilution. The ECL detection kit (GE Healthcare, Piscataway, NJ) was used to detect the labeling of the antibodies.

## Supporting Information

File S1 Additional monoclonal cell lines that did not meet are criteria to be described in main text.
(0.32 MB DOC)

File S2 Supplemental methods detailing construction of immunogen expressing clones.
(0.13 MB DOC)

Table S1 List of the Supplemental mAbs.
(0.07 MB DOC)
