# Supplementary material for: A Monoclonal Antibody Toolkit for C. elegans
Source: PLoS One. 2010 Apr 13;5(4):e10161. doi: 10.1371/journal.pone.0010161 (PMC2854156; doi:10.1371/journal.pone.0010161)
Supplement: File S1 — Additional monoclonal cell lines that did not meet are criteria to be described in main text. (0.32 MB DOC) [file pone.0010161.s001.doc]

The following document describes monoclonal lines isolated that we do not feel confident recognize the predicted products in whole mounts immunohistochemistry or Westerns (or both), even through the specifically detect the immunogen used by ELISA assays.

**UAF-1:**

*uaf-1* encodes the large protein subunit of the splicing factor U2AF, a component of the spliceosome [1]. Monoclonal antibodies against UAF-1 were made by immunizing mice with a full length His_6_-UAF-1 fusion protein. Two stable hybridoma cell lines, 3C5 and 5C5, were obtained and all were of the isotype IgG1. Both monoclonals were used to examine the immunostain pattern on whole mount wild type animals. The monoclonals were found to stain in a pattern that was similar to what had previously been reported for an α-UAF-1 polyclonal antibody, which is predominantly nuclear [2]. Similarly, a monoclonal antibody to mammalian U2AF^65^ was found to label the protein in the nucleoplasm and in nuclear speckles [3]. Immunoblots of *C. elegans* wild type lysates were probed with the monoclonal antibodies. Only the 3C5 monoclonal antibody works and recognized protein bands of 120 kDa, 70 kDa, 68 kDa, 64 kDa, 38 kDa, and 16 kDa, with the 38 kDa band the most prominent. In Bouin’s pre-fixed worms that were then extracted with SDS-PAGE buffer, 38 kDa and 200 kDa protein bands were observed with this monoclonal. The predicted sizes of the isoforms are: 16 kDa, 53 kDa, and 55 kDa. Previously, the polyclonal antibody was reported to recognize a 65 kDa protein [1].

**NCA-1:**

*nca-1* encodes a novel cation channel with similarity to calcium and sodium channels [4,5]. A polyclonal antibody directed against the C-terminal half of the *C. elegans* NCA-1c isoform consisting of amino acids: 1731-1914 detects the endogenous protein in the nervous system.[6]. The *C. elegans* NCA-1 monoclonal antibodies were made by immunizing mice with a His6-tagged NCA-1 fusion protein consisting of amino acids 1160-1861 of the NCA-1d isoform. This domain included the same domain used to make the polyclonal antibodies with an additional 29 amino acids in the middle. Three stable hybridoma cell lines (3G12, 11G5, and 13F6) were produced and found to be the isotype IgG1. On whole mount immunostain of wild type *C. elegans*, an immunostain pattern was seen that had not been previously reported with the polyclonal antibody. It labeled outside of the nucleus and labeled cell bodies. Analysis of the 3G12 monoclonal antibody on western blots of wild type animal lysates showed that it recognized a protein band of 215 kDa, which is close to the predicted size of the protein. A band of 220 kDa was also seen, but of lower intensity. This band is close to the predicted size of the NCA-1c isoform. Thus, the monoclonal may recognize both isoforms of NCA-1 on westerns.

**HIM-3:**

*him-3* encodes an HORMA domain protein that’s a homolog of yeast HOP1 protein, which is thought to act on chromatin for the formation of the synaptonemal complex during meiosis [7]. Monoclonal antibodies were made against the C-terminus of HIM-3 including amino acids, 105-291. This domain is very similar to the domain that was reported for the production of polyclonal antibodies, amino acids 107-291 [7]. Four stable hybridoma cell lines (11.2.3, 11.6.1, 11.8.1, and 11.12.2) were produced and all were isotyped as IgG1. These monoclonal antibodies were used to stain wild type whole mount animals and were found to label hypodermal cells, gonad, nervous system, and intestine. This is not in agreement with what has been reported for the staining pattern of the polyclonal antibody: condensing chromosomes in early prophase I and to the cores of synapsed and unsynapsed chromosomes located in the gonad [7]. Western blot analysis of wild type lysates probed with these monoclonal antibodies revealed bands of 38 kDa, 80 kDa, 92 kDa, and 150 kDa,. The predicted size of the HIM-3 protein is 33 kDa. A western blot that was reported previously on yeast lysates probed with an α-HOP1 antibody, revealed 70 kDa, 33 kDa, and 30 kDa bands [8].

**SQV-2:**

*sqv-2* encodes a homolog of the Golgi resident enzyme glycosaminoglycan galactosyltransferase II (Gal-T2) which is involved in post-translational protein glycosylation [9]. A His_6_-tagged full length SQV-2 fusion protein was used to immunize mice. Five stable hybridoma cell lines (3B1, 3C1, 4A4, 5F1, 10D11) were produced and all were found to be of the isotype IgG1. On whole mount immunostaining of wild type *C. elegans*, all of the antibodies exhibited similar staining patterns labeling structures (probably Golgi) around the nucleus and also stain the vulva as has been previously reported for the anti-SQV-2 polyclonal antibodies [10]. On western blots of wild type *C. elegans* lysates, a 47 kDa protein band was recognized by the 10D11 monoclonal antibody in reasonable agreement with the 38 kDa predicted Mw of SQV-2 . However, reactivity of this monoclonal was also strong to the 50 kDa protein of the Precision Plus Protein All Blue standards (Biorad).

**AEX-6 (RAB-27):**

*aex-6* encodes a Rab27 homolog of the Rab family of GTP-binding proteins and several lines of evidence suggests it associates with dense core vesicles [11,12]. Monoclonal antibodies were made by injecting mice with a His6-tagged fusion protein to AEX-6, amino acids 1-212. This is the same domain that was used to make the polyclonal antibodies [11]. Eight stable hybridoma cell lines, 9H4, 10D1, 10E10, 10F3, 11A10, 12B2, 12G3, and 12H3, were obtained and were all of the isotype IgM. On whole mount immunostain of wild type *C. elegans,* an immunostain pattern was observed that labeled body-wall muscle, sphincter and anal depressor muscle, and faintly labeled head neurons. The polyclonal antibody was reported to be detected on whole mounts in the nervous system, and the GFP-construct was reported to be detected both in the nervous system and in the intestine [11]. On western blots of wild type *C. elegans* lysate, protein bands of 37-50 kDa, 70 kDa, and 110 kDa were detected with the 12H3 and 10F3 monoclonals. No protein bands could be detected with the other monoclonals.

**RAB-3:**

*rab-3* encodes a rab3 homolog of the Rab family of GTP-binding proteins which is associated with synaptic vesicles [13]. The *C. elegans* monoclonal antibodies against RAB-3 were made by immunizing mice with a His6-tagged fusion protein consisting of amino acids, 1-219 of the RAB-3b isotype. This is almost the identical region that was used to make α-RAB-3 polyclonal antibodies, amino acids 6-219 [13]. Two stable hybridoma cell lines, 9F11 and 13G8, were produced and 9F11 was isotyped as IgG2 and 13G8 was isotyped as IgG1. On whole mounts, the monoclonals were found to not stain in a pattern that had been reported for the polyclonal antibody: nerve ring, dorsal cord, and ventral cord [13]. Instead, the monoclonal antibodies stained muscle, pharynx, and undetermined cell types. Western blot analysis of wild type *C. elegans* lysate with the monoclonals revealed that the 13G8 antibody recognized a protein band of 250 kDa, whereas no protein bands could be detected with the 9F11 antibody.

**UNC-64:**

*unc-64* encodes a neuronal syntaxin that is involved in vesicle fusion and localizes relatively uniformly to the plasma membrane of neurons [14]. Monoclonal antibodies were made against a His6-tagged UNC-64 fusion protein, full length minus the last 26 amino acids which a polyclonal antibody had also been made against [14]. Three stable hybridoma cell lines, 5E2, 8B11, and 8D2, were produced and all were the isotype IgM. Whole mount immunostain of wild type *C. elegans* produced no staining pattern. On western blots of wild type *C. elegans* lysates, the 5E3 monoclonal antibody recognized an 58 kDa band with an anti-IgM secondary antibody, and in the mutant strain, *unc-64(md130),* an 15X decrease was observed in the intensity of the 58 kDa band. This is in good agreement with what has been reported for the UNC-64 polyclonal antibody [14]. It is possible that this antibody recognizes specific syntaxin in a SNARE complex, but not syntaxin monomers.

Since staining on whole mount animals with the first set of monoclonals was not observed, a second attempt to make monoclonal antibodies to UNC-64 was made by immunizing mice with a C-terminus His6-tagged UNC-64 fusion protein minus the last 26 amino acids. Two stable hybridoma cell lines, 2G6 and 2H4, were obtained and all were the isotype IgG1. Whole mount immunostaining with these antibodies was mixed. Neuronal staining and staining of coelomocytes was observed as had been reported for the UNC-64 polyclonal antibody [14], but staining of what looked like intestinal nuclei and muscle was also observed. Since the 2G6 monoclonal antibody seemed to be of a higher titer, it was analyzed on western blots of wild type *C. elegans* lysates. Two protein bands with a strong intensity of 68 kDa and 56 kDa were observed, and two protein bands with a lower intensity of 36 kDa and 20 kDa were also observed.. In mutant lysates, *unc-64(md130)*, the 69 kDa and 36 kDa bands were the only two protein bands to stain with a lower intensity, 15X as compared to wild type. Thus, the UNC-64 monoclonal antibody, 2G6, recognizes at least two different proteins (with a similar epitope) and is not specific to just the UNC-64 protein.

**UNC-29:**

*unc-29* encodes a nicotinic AChR, a non-alpha subunit of the nicotinic acetylcholine receptor family [15]. Monoclonal antibodies were made by immunizing mice with a GST-tagged UNC-29 fusion protein, amino acids 348-431. This was the same domain which a polyclonal antibody had been made to [16]. Three stable hybridoma cell lines were produced, 4A9, 5H1, and 6B8, and all were isotyped as IgG1, except for 5H1 which was isotyped as IgM. Whole mount immunostaining of wild type *C. elegans* animals revealed staining of muscle and nervous system. The polyclonal antibody was reported to stain the nervous system: dorsal cords, ventral cords, and nerve ring [16]. The GFP-UNC-29 fusion expression construct was reported to stain body muscles and a neuronal cell body [15]. Analysis of these monoclonal on westerns of *C. elegans* lysates revealed that no protein bands could be detected except with the 5H1 monoclonal. The 5H1 monoclonal detected protein bands of 110 kDa, and the expected size of the UNC-29 protein is 56 kDa.

**SAS-5:**

*sas-5* encodes a coiled coil protein required for daughter centriole formation [17]. Monoclonal antibodies were made by immunizing mice with a full length GST-tagged fusion protein (a generous gift from P. Gönczy). This is the same fusion protein that had been utilized previously for the polyclonal antibody [17]. Two stable hybridoma cell lines were produced, 1B1 and 7B2, and all were isotyped as IgM. The monoclonal antibodies did not stain wild type whole mounts in a pattern that was reported for the polyclonal and the GFP-construct. Instead of centriole staining, the monoclonals stained the nucleus of embryos [17,18]. Western blot analysis of wild type lysates revealed that the monoclonal antibodies recognized several protein bands that were not the expected size for the protein which was 46 kDa. Instead, the 1B1 monoclonal labeled a protein band greater than 300 kDa and protein bands less than 30 kDa. The 7B2 monoclonal labeled protein bands of 88 kDa and 37 kDa.

**EEA-1:**

*eea-1* encodes an ortholog of early endosome antigen 1[19]. Monoclonal antibodies were made by immunizing mice with a His6-tagged fusion protein, amino acids 973-1205. This domain is the same one that the polyclonal antibody had been previously made against [20]. Four stable hybridoma cell lines, 2C2, 2F10, 3E5, and 4D6, were produced and all were isotyped as IgM. Immunostaining of *C. elegans* animals revealed a staining pattern that labeled internal structures in a punctate pattern, but these structures were not identified. Cortical puncta were visualized in embryos with the polyclonal antibody and GFP-expression construct [21,22]. No protein bands could be detected on western blots of wild type *C. elegans* lysates, except with the 2C2 monoclonal antibody which detected protein bands of predominantly 10 kDa, 15 kDa, 25 kDa, with fainter protein bands detected at 100 kDa, 150 kDa, and 250 kDa.

**RME-2:**

*rme-2* encodes a member of the low-density lipoprotein (LDL) receptor family of yolk receptors. *rme-2* is expressed in developing oocytes and in the proximal region of the germ line [23,24]. Monoclonal antibodies against *C. elegans* RME-2 were made by immunizing mice with a His6-tagged fusion protein to amino acids 180-670, the same region as the RME-2-EXT polyclonal antibody [23]. Four stable hybridoma cell lines, 1B10, 1F2, 3C4, and 4C3, were produced and all were isotyped as IgM, except for 4C3 which was an IgG1. On whole mount immunostain of wild type *C. elegans*, a pattern that was different from what had been reported for the polyclonal antibody was observed. Muscle staining was seen with the IgM monoclonals, and the 4C3 monoclonal stained pharynx, oocytes, endosomal like structures, and uterus. Analysis of these monoclonals on western blots revealed that no protein bands could be detected; whereas, the polyclonal could detect a band of 110 kDa [23].

**RME-8:**

*rme-8* encodes a J-domain protein that functions in endocytosis in many cell types in *C. elegans* [25]. RME-8 monoclonal antibodies were made by immunizing mice with amino acids, 2065-2256, which is similar to the domain that a polyclonal antibody was made to, amino acids 2064-2270 [25]. Three stable hybridoma cell lines, 8H5, 9B4, and 9F3, were made and isotyped as IgM, except for an IgG1, 9B4. Upon examining the monoclonals on wild type animals, the pattern of immunostaining was similar to what had been previously reported for the polyclonal antibody: hypodermis, muscle, gonad, spermatheca, and intestine [25,26]. Analysis of these monoclonals on western blots of wild type animals resulted in no protein bands being stained, unlike the polyclonal antibody which recognized a protein band of 260 kDa [25].

**PAR-1:**

*par-1* encodes a serine-threonine kinase that plays a role in establishing early embryonic polarity and for morphogenesis of the vulva of *C. elegan*s [27,28,29]. A His6-tagged fusion protein, amino acids 453-674, was used to immunize mice in the production of PAR-1 monoclonal antibodies. Polyclonal antibodies had previously been made to PAR-1 using this same domain and to amino acids 780-12132 [28]. Two stable hybridoma cell lines, 3F6 and 3F10, were produced and were isotyped as IgG1. On whole mount immunostain of wild type *C. elegans*, a very weak diffuse staining of embryos was observed. Analysis of these monoclonals on western blots of wild type animal lysates revealed that no protein bands were detected.

**PAR-6:**

*par-6* encodes a PDZ-domain protein that plays an important role in the polarity of *C. elegans* embryos [30,31]. Monoclonal antibodies to PAR-6 were made by immunizing mice with a full length His6-tagged fusion protein, which is the same domain that had previously been used to produce polyclonal antibodies [30]. Two hybridoma cell lines, 3H9 and 8H8, were produced and were all isotyped as IgG1. On whole mount immunostain of wild type animals, an immunostain pattern was observed that was similar to what had been reported for the GFP- construct and polyclonal antibody: throughout the cytoplasm and periphery of embryos, apical regions of intestine, pharynx, and spermatheca [30,31,32]. On analysis of the monoclonals on western blots of wild type extracts, a protein band of 33 kDa, 38 kDa, and 125 kDa was observed. The predicted size of PAR-6 is 38 kDa. The polyclonal antibody was predicted to detect a protein band 34 kDa [30].

**GLR-1:**

*glr-1* encodes an AMPA (non-NMDA) type ionotropic glutamate receptor subunit [33,34]. GLR-1 monoclonal antibodies were made by immunizing mice with a His6-tagged fusion protein, amino acids 871-962. Polyclonal antibodies had previously been made to the ectodomain [35].

One stable hybridoma cell line was obtained, 4G11, and was isotyped as IgG2b. Whole mount immunostaining of *C. elegans* animals revealed staining of muscle, nervous system, and unknown cell types. The polyclonal, GFP-, and YFP-constructs were reported to label nerve ring, ventral cord, specific neurons, and interneuron cell bodies, [33,34,36,37,38,39]. Western blots of *C. elegans* wild type lysates probed with the GLR-1 monoclonal antibody, revealed that the antibody recognized a protein band of 76 kDa which is not in agreement with the expected size of the protein which is 108 kDa.

**EAT-4:**

*eat-4* encodes an ortholog of BNPI, a mammalian brain-specific sodium-dependent inorganic phosphate co-transporter I involved in glutamate transport into vesicles [40,41]. Monoclonal antibodies against EAT-4 were made by immunizing mice with a His6-tagged fusion protein, amino acids 508-576. This is the same domain that a polyclonal antibody had been made against, where a rat GST-BNPI fusion protein consisted of the last 68 amino acids [42]. Two stable hybridoma cell lines were obtained, 2A5 and 4B10, and were all isotyped as IgG1. On whole mount immunostain of wild type animals, a pattern of immunostaining was observed that labeled muscle and other cell types. This staining pattern did not match what had previously been reported for the EAT-4 polyclonal, GFP- and lacZ-constructs, which detected the endogenous protein in a subset of *C. elegans* pharyngeal neurons and glutamatergic neurons [41,43]. Western analysis of wild type lysates with the monoclonal antibodies revealed that they recognized a band of 75 kDa and 115 kDa. The polyclonal had previously been shown to recognize a protein band in rat brain and COS cells of 60 kDa [42].

**TRAP-2:**

*trap-2* encodes the beta subunit for the translocon-associated complex localized to the ER (endoplasmic reticulum) membrane [44]. A His6-tagged fusion protein, amino acids 1-188, was used to immunize mice. Four stable hybridoma cell lines, 5F3, 7D6, 13B9, and 15C1, were isolated and all were isotyped as IgG1. On whole mount immunostain of wild type *C. elegans*, a pattern of immunostaining was seen that was not similar to what had been reported for the YFP-construct, cell bodies of neurons [44]. Instead, diffuse staining could be seen in different cells throughout the animal. Western analysis of wild type *C. elegans* lysate with the monoclonals revealed that no protein bands could be detected.

**CUP-5:**

*cup-5* encodes an ortholog of the human mucolipin-1 gene, TRPML1[45,46]. To make monoclonals, a His6-tagged fusion protein, amino acids 550-668, was made to the CUP-5d isoform and injected into mice. Previously a polyclonal antibody had been made to the amino and carboxyl terminus of TRPML1, amino acids 1-70 and 520-576[47]. A polyclonal antibody to rat mucolipin-1 had also been made to amino acids 99-282 [48]. Three stable hybridoma cell lines were obtained, 2F8, 5G10, and 7D4, and all were isotyped as IgG1. On whole mount immunostaining of *C. elegans*, all of the antibodies exhibited similar staining patterns labeling spermatocytes very strongly, with faint staining observed in gonad and possibly neurons. GFP-constructs were previously reported to strongly label coelomocytes, cytoplasmic vesicles, and plasma membrane-proximal structures in neurons [45,49]. No coelomocyte labeling was seen with the monoclonals. Western analysis of wild type *C. elegans* lysates revealed that the monoclonals recognized several protein bands. The 5G10 monoclonal recognized a protein band of 75 kDa, the 2F8 monoclonal recognized protein bands of 43 kDa and 73 kDa, and the 7D4 monoclonal recognized protein bands of 46 kDa, 75 kDa and 110 kDa on westerns. The predicted size of the CUP-5 protein is 70-76 kDa. The α-TRPML1 polyclonal antibody recognized protein bands of 35-40 kDa and 65 kDa on western blots of HEK 293 lysates [47]. The α-rat-mucolipin-1 polyclonal antibody recognized a protein band of 60 kDa band on western blots of rat liver fractions that was enriched in lysosomes, and 34, 40, and 55 kDa protein bands in the late endosome fractions [48].

**ELKS-1:**

*elks-1* encodes a homolog of ELKS, ERC, and CAST, which are active zone proteins [50]. *C. elegans* monoclonal antibodies against ELKS-1, were made by immunizing mice with a His6-tagged fusion protein that was the same fusion protein used for making polyclonal antibodies [50]. Three stable hybridoma cell lines were produced, 2A9, 2F5, and 3D3, and all were isotyped as IgG1. The monoclonal antibodies were examined on whole mounts of wild type animals and a staining pattern was observed with immunolabeling of gonad, muscle, nervous system, and unidentified cells. The monoclonals did not stain nervous system which has been reported for the polyclonal antibody [50]. On western blots of wild type lysates, the monoclonals detected a protein band of 73 kDa. This does not match with the predicted size of the protein which is 96 kDa [50].

These monoclonal lines were also obtained as a repeat fusion:

TAC-1:

Mice were injected with a GST-tagged fusion protein of full length TAC-1 (a generous gift from P. Gönczy). Four stable hybridomas, 1A5, 1E9, 2A3, and 2F5, were produced and were all isotyped as IgM. On whole mounts, the monoclonals immunostained nuclei, which is what had not been reported with the polyclonal antibody [51]. On western blots of wild type extracts, the 1A5 and 2F5 monoclonals did not label any protein bands. The 1E9 monoclonal labeled protein bands of 10-15 kDa, 100 kDa, and 250 kDa, on westerns, and the 2A3 monoclonal labeled protein bands of 10-20 kDa and 250 kDa on westerns.

None of these monoclonals have been submitted to the DSHB, but we will make every effort to them available to researchers who wish to perform further analysis to determine what they are detecting.

**Materials and Methods**

**Fusion Protein purification:**

The following fusion proteins were purified via native conditions and dialyzed against PBS: SAS-5, UNC-64, AEX-6, RAB-3, UNC-29, RME-2, RME-8, EEA-1, and ELKS-1. The following fusion proteins were purified via non-native conditions (8M urea) and renatured by dialysis in PBS: UAF-1, SQV-2, HIM-3, NCA-1, PAR-1, LET-413, GLR-1, EAT-4, PAR-6, TRAP-2, CUP-5.

**Vectors used for constructs:**

pRSETA, pRSETB, pRSETC His6-tag vectors (Invitrogen, Carlsbad, CA)

pHO2d His6 tag vector [52 ; gift from P. Hanson]

pHO4d His6-Myc-tag vector[53 ; gift from P. Hanson]

pET24b His6-tag vector (Invitrogen, Carlsbad, CA)

pQE32 His6-tag vector (Qiagen, Valencia, CA)

NM1986, pVM, is a modification of the vector, pRSETA, with a linker: 5’- CCATGGAATTCGGCGGCCGCATAGTACCTAAGTACTAGACAAGCTT, cloned into the NcoI/HindIII site of pRSETA.

pET SUMO-His6-tag vector (Invitrogen; Carlsbad, CA)

NM1840, pSUMOpST, is a modification of the vector, pET SUMO (Invitrogen) with a linker: 5’-AGCGACCAAGTGGGCGGTCCGGGATCCGGAGCTCGAGATCTGCAGCTGGTACCATGGAATTCGGCGGCCGCATAGTACCTAAGTACTA, cloned into the vector, pET SUMO by TOPO TA cloning (Invitrogen).

pGEX2T,pGEX3X, pGEX4T-1 GST-tag vectors GE Healthcare, Piscataway, NJ)

pBHA yeast shuttle vector with LexA DNA-binding domain [54 ; Clontech, Palo Alto, CA]

**Expression Vector Construction**

**UAF-1 constucts:**

NM1461, pGEX-2T:UAF-1, included a full length N-terminus GST tagged domain of UAF-1 (U2AF65), amino acids 1-496, cloned into the pGEX-2T vector (gift from T. Blumenthal and A.R. Zorio).

NM1494, SUAF1/pRSETA, UAF-1, amino acids 1-171, was an intermediate construct derived from NM1461. NM1461 was digested with BamHI/PstI and the 514 bp fragment was cloned into the BamHI/PstI site of the vector pRSETA.

NM1514, UAF-1/pRSETA, included a full length N-terminus His6 tagged domain of UAF-1 from NM1461, amino acids 1-496. NM1461 was digested BsrGI/HindIII and the fragment was cloned into the BsrGI/HindIII site of the vector NM1494, SUAF1/pRSETA.

NM1494:

ATGCGGGGTTCTCATCATCATCATCATCATGGTATGGCTAGCATGACTGGTGGACAGCAAATGGGTCGGGATCTGTACGACGATGACGATAAGGATCGATGGGGATCCATGAGTGATCACCAGGATGGCATGAAACTTGAAGATATTGAACGCCAATTTTTGGACGTTGCTCAACGTGAAGGAGGTCTCGAAGCGATTCAGCCGACCACTGGACCACTGGAAAATGAAGAAAATCTGAAATCATCGACTGGTGGTGGTGGTGGAGAGGATGATAATGATCGGAAAAAGAGAAAACGATCGAGAAGTAGAGATCGCGATACGAGAAGACGCTCGAGAAGTCGTGATCGTGGAGAACGTCGTGGTGGTGGAGGTGGAGGTGATCGTGATCGTTCTCGATCCCGTGAACGTCGTCGAGGCGGTGGTGGTCGTGATGAGCCGAGACGTCGTGGAGGTGATGATGAAGCCCGTTCGCGCCGTGAGCCGGAGCCCCAGAAGCCTCGTGAGCCCAAAAAGTACCGATTCTGGGATGTTCCGCCGACTGGATTCGAGACAACTACACCGATGGAGTACAAGAATATGCAGGCGGCTGGACAGGTTCCACGTGGATCTGTACAGTCTGCAGCTGGTACCATGGAATTCGAAGCTTGA

NM1514:

ATGCGGGGTTCTCATCATCATCATCATCATGGTATGGCTAGCATGACTGGTGGACAGCAAATGGGTCGGGATCTGTACGACGATGACGATAAGGATCGATGGGGATCCATGAGTGATCACCAGGATGGCATGAAACTTGAAGATATTGAACGCCAATTTTTGGACGTTGCTCAACGTGAAGGAGGTCTCGAAGCGATTCAGCCGACCACTGGACCACTGGAAAATGAAGAAAATCTGAAATCATCGACTGGTGGTGGTGGTGGAGAGGATGATAATGATCGGAAAAAGAGAAAACGATCGAGAAGTAGAGATCGCGATACGAGAAGACGCTCGAGAAGTCGTGATCGTGGAGAACGTCGTGGTGGTGGAGGTGGAGGTGATCGTGATCGTTCTCGATCCCGTGAACGTCGTCGAGGCGGTGGTGGTCGTGATGAGCCGAGACGTCGTGGAGGTGATGATGAAGCCCGTTCGCGCCGTGAGCCGGAGCCCCAGAAGCCTCGTGAGCCCAAAAAGTACCGATTCTGGGATGTTCCGCCGACTGGATTCGAGACAACTACACCGATGGAGTACAAGAATATGCAGGCGGCTGGACAGGTTCCACGTGGATCTGTACAGTCTGCAGTGCCCGTTGTTGGCCCATCAGTGACTTGTCAATCACGTCGTCTCTACGTTGGAAATATTCCGTTCGGATGCAATGAAGAAGCTATGCTCGACTTTTTCAATCAACAAATGCACCTCTGTGGATTGGCTCAAGCGCCTGGAAATCCGATTCTTCTGTGCCAAATAAATTTGGACAAAAATTTCGCATTCATAGAATTCCGTTCAATAGACGAAACGACGGCCGGAATGGCATTTGACGGAATCAATTTCATGGGACAACAGTTGAAAGTTCGACGTCCAAGAGACTATCAACCATCTCAAAATACGTTTGATATGAACTCTCGTATGCCTGTTTCAACGATTGTCGTCGATTCGGCCAATAAGATCTTTATCGGTGGTCTGCCCAACTATTTGACTGAGGATCAGGTAAAAGAGCTTTTGTGCTCATTTGGACCGCTTAAGGCGTTCTCGTTGAACGTTGATTCGCAAGGAAATAGCAAAGGATACGCGTTCGCCGAATATTTGGACCCAACTCTCACTGATCAGGCCATCGCCGGGCTCAACGGTATGCAACTCGGCGACAAGCAGCTCGTCGTTCAACTTGCCTGTGCTAATCAACAACGTCATAACACAAATCTGCCGAACTCTGCGTCAGCAATTGCCGGAATTGATCTCTCCCAAGGAGCCGGCCGAGCCACTGAGATCCTTTGTCTCATGAATATGGTGACTGAGGATGAGCTGAAAGCTGATGATGAGTACGAAGAGATTCTGGAGGATGTACGCGATGAATGCTCAAAATATGGAATTGTTCGCTCGTTGGAAATTCCAAGACCGTATGAGGATCATCCGGTTCCAGGTGTTGGGAAGGTTTTCGTCGAATTCGCCTCCACATCCGACTGTCAACGTGCTCAGGCTGCTCTGACCGGACGAAAATTCGCGAATCGCACCGTCGTCACCTCCTACTATGACGTCGACAAGTACCACAATCGTCAATTCTAAgcccatggaattcgaagctt

**NCA-1 Constructs:**

NM1922, NCA-1/SUMOpST, included a N-terminus His6-Myc tagged NCA-1d, amino acids 1660–1861. Primers, #3315: 5’-AGGGGATCCGCGATGGATGCAGAAGGAGTACTTC and #3316: 5’-ATAGTTTAGCGGCCGCCTAATCAACAAGGGAATTC, were used. The fragment was cloned into the BamHI/NotI site of the vector SUMOpST .

NM2001, NCA-1/pRSETA, included a N-terminus His6-tagged NCA-1d from NM1922. NM1922 was digested with BamHI/HindIII and cloned into the BamHI/HindIII site of the vector pRSETA.

NM1887, NCA-1/pGEX-4T, included a N-terminus GST tagged NCA-1d, amino acids 1660 -1861. Primers, #3315: 5’-AGGGGATCCGCGATGGATGCAGAAGGAGTACTTC and #3316: 5’-ATAGTTTAGCGGCCGCCTAATCAACAAGGGAATTC, were used. The fragment was cloned into the BamHI/NotI site of the vector pGEX4T-1.

NM1922:

ATGGGCAGCAGCCATCATCATCATCATCACGGCAGCGGCCTGGTGCCGCGCGGCAGCGCTAGCATGTCGGACTCAGAAGTCAATCAAGAAGCTAAGCCAGAGGTCAAGCCAGAAGTCAAGCCTGAGACTCACATCAATTTAAAGGTGTCCGATGGATCTTCAGAGATCTTCTTCAAGATCAAAAAGACCACTCCTTTAAGAAGGCTGATGGAAGCGTTCGCTAAAAGACAGGGTAAGGAAATGGACTCCTTAAGATTCTTGTACGACGGTATTAGAATTCAAGCTGATCAGACCCCTGAAGATTTGGACATGGAGGATAACGATATTATTGAGGCTCACAGAGAACAGATTGGTGGTAGCGACCAAGTGGGCGGTCCGGGATCCGCGATGGATGCAGAAGGAGTACTTCCATTTGGTGGAGGACATCCGGTTATTCATTCATCAGGACATTCTTCAATTTCACATGAGGAAACTGTTGCACAAAGACTTCGATTTGAAAATATTCGAGGAGATAGTGTTGATACAGAAGAGACGGAGAGTAGTGAAGAAGAAACTCCACCTCCAATAAGGAAGAAGGCTGCTGTTAAGAATCGAAGAGGAAGTATTCCAGATGTTCTTTCGAGAACTGGTCTCTTTCAAGAAGCTGCTCGAAAGTTCATGGTTGGTAGCTCGAGTGAGAAAAAACAAGTCAAGTCGCGATCGCCTGAAACCGTCCAGTTGCTTCCGAAACGAGCTAATTCTGAAATCCGAAAGGGTTCCGGACAGCCAAAGAATTTTCATCTCCAGCTAAACGTCTATGATCTACCTGACGTAGAAGAACGTGGAGAAGATTCTCCATTTTCTCCCAAAAACTTGTCTGATGATTTTAATGGAGAGCATTCACCATTGGTGATAACTCCATCCTTGCCTGTACCACCGACACATGGAAGCCCTCGACCACTTATGCCATGTGAAACGACAAAAGATATTGAAAAATGGTGGAATTCCCTTGTTGATTAGgcggccgc

NM2001:

ATGCGGGGTTCTCATCATCATCATCATCATGGTATGGCTAGCATGACTGGTGGACAGCAAATGGGTCGGGATCTGTACGACGATGACGATAAGGATCGATGGGGATCCGCGATGGATGCAGAAGGAGTACTTCCATTTGGTGGAGGACATCCGGTTATTCATTCATCAGGACATTCTTCAATTTCACATGAGGAAACTGTTGCACAAAGACTTCGATTTGAAAATATTCGAGGAGATAGTGTTGATACAGAAGAGACGGAGAGTAGTGAAGAAGAAACTCCACCTCCAATAAGGAAGAAGGCTGCTGTTAAGAATCGAAGAGGAAGTATTCCAGATGTTCTTTCGAGAACTGGTCTCTTTCAAGAAGCTGCTCGAAAGTTCATGGTTGGTAGCTCGAGTGAGAAAAAACAAGTCAAGTCGCGATCGCCTGAAACCGTCCAGTTGCTTCCGAAACGAGCTAATTCTGAAATCCGAAAGGGTTCCGGACAGCCAAAGAATTTTCATCTCCAGCTAAACGTCTATGATCTACCTGACGTAGAAGAACGTGGAGAAGATTCTCCATTTTCTCCCAAAAACTTGTCTGATGATTTTAATGGAGAGCATTCACCATTGGTGATAACTCCATCCTTGCCTGTACCACCGACACATGGAAGCCCTCGACCACTTATGCCATGTGAAACGACAAAAGATATTGAAAAATGGTGGAATTCCCTTGTTGATTAGgcggccgcatagtacctaagtactagacaagctt

NM1887:

ATGTTCGAAGATCGTTTATGTCATAAAACATATTTAAATGGTGATCATGTAACCCATCCTGACTTCATGTTGTATGACGCTCTTGATGTTGTTTTATACATGGACCCAATGTGCCTGGATGCGTTCCCAAAATTAGTTTGTTTTAAAAAACGTATTGAAGCTATCCCACAAATTGATAAGTACTTGAAATCCAGCAAGTATATAGCATGGCCTTTGCAGGGCTGGCAAGCCACGTTTGGTGGTGGCGACCATCCTCCAAAATCGGATCTGGTTCCGCGTGGATCCGCGATGGATGCAGAAGGAGTACTTCCATTTGGTGGAGGACATCCGGTTATTCATTCATCAGGACATTCTTCAATTTCACATGAGGAAACTGTTGCACAAAGACTTCGATTTGAAAATATTCGAGGAGATAGTGTTGATACAGAAGAGACGGAGAGTAGTGAAGAAGAAACTCCACCTCCAATAAGGAAGAAGGCTGCTGTTAAGAATCGAAGAGGAAGTATTCCAGATGTTCTTTCGAGAACTGGTCTCTTTCAAGAAGCTGCTCGAAAGTTCATGGTTGGTAGCTCGAGTGAGAAAAAACAAGTCAAGTCGCGATCGCCTGAAACCGTCCAGTTGCTTCCGAAACGAGCTAATTCTGAAATCCGAAAGGGTTCCGGACAGCCAAAGAATTTTCATCTCCAGCTAAACGTCTATGATCTACCTGACGTAGAAGAACGTGGAGAAGATTCTCCATTTTCTCCCAAAAACTTGTCTGATGATTTTAATGGAGAGCATTCACCATTGGTGATAACTCCATCCTTGCCTGTACCACCGACACATGGAAGCCCTCGACCACTTATGCCATGTGAAACGACAAAAGATATTGAAAAATGGTGGAATTCCCTTGTTGATTAGgcggccgc

**HIM-3 Constructs:**

NM2025, HIM-3/pVM, included a N-terminus His6 tagged domain of HIM-3, amino acids 105-291. Primers, #3372: 5’-AGGGGATCCGCGATGGTGTTCTACAAGAACGAGG and #3373: 5’-ATAGTTTAGCGGCCGCCTAGTCCTTATTCTTCTTCGT, were used. The fragment was cloned into the BamHI/NotI site of the vector pVM.

NM2048, HIM-3/pGEX4T-1, included a N-terminus GST tagged domain of HIM-3 from NM2025. NM2025 was digested with BamHI/NotI and the fragment was cloned into the BamHI/NotI site of the vector pGEX4T-1.

NM2025:

ATGCGGGGTTCTCATCATCATCATCATCATGGTATGGCTAGCATGACTGGTGGACAGCAAATGGGTCGGGATCTGTACGACGATGACGATAAGGATCGATGGGGATCCGCGATGGTGTTCTACAAGAACGAGGAAGACATTAATGAAGTGTTTGCGTTCAGGTTTGCTTATGGAGATGAAGGAGAGATTTTCGTATCTCTAAATAACGGAATCGACACGAACGAAAGCAGTCAAGAACTGTTACAGGCGAAATTCGTTGATACCGACAACACGAAACAAATGTTTGCAAGCACAATCAAGAAACTTCACCGGTGTATCAAGAAAATGGAGCCGCTTCCTCAAGGGTCCGACGCGAGTTTCCGTGTTTCGTACACTGAAAAAGCTCCAAAAGACTATACACCCGAAGGCTATCTGCTGAGTCCTATGTTCTATACGCTGAATCAGGATATTAGGAAAGCATCTATTGGAATCGTGTGTGGCGGTCATCACAAAATTCAAATGCTTGCTGCTTCACAATACTTGAAGCAAGACTTCGATCTGGATAAAACGACCACACTCAATCCAAATATGTCAATCATGGCAAACCAATCGAAAAGGAAGGGACGAATTTCCAGAGATTCCCCTTACGGTCTCAGTCAGGGCATTACGAAGAAGAATAAGGACTAGgcggccgc

NM2048:

ATGTTCGAAGATCGTTTATGTCATAAAACATATTTAAATGGTGATCATGTAACCCATCCTGACTTCATGTTGTATGACGCTCTTGATGTTGTTTTATACATGGACCCAATGTGCCTGGATGCGTTCCCAAAATTAGTTTGTTTTAAAAAACGTATTGAAGCTATCCCACAAATTGATAAGTACTTGAAATCCAGCAAGTATATAGCATGGCCTTTGCAGGGCTGGCAAGCCACGTTTGGTGGTGGCGACCATCCTCCAAAATCGGATCTGGTTCCGCGTGGATCCGCGATGGTGTTCTACAAGAACGAGGAAGACATTAATGAAGTGTTTGCGTTCAGGTTTGCTTATGGAGATGAAGGAGAGATTTTCGTATCTCTAAATAACGGAATCGACACGAACGAAAGCAGTCAAGAACTGTTACAGGCGAAATTCGTTGATACCGACAACACGAAACAAATGTTTGCAAGCACAATCAAGAAACTTCACCGGTGTATCAAGAAAATGGAGCCGCTTCCTCAAGGGTCCGACGCGAGTTTCCGTGTTTCGTACACTGAAAAAGCTCCAAAAGACTATACACCCGAAGGCTATCTGCTGAGTCCTATGTTCTATACGCTGAATCAGGATATTAGGAAAGCATCTATTGGAATCGTGTGTGGCGGTCATCACAAAATTCAAATGCTTGCTGCTTCACAATACTTGAAGCAAGACTTCGATCTGGATAAAACGACCACACTCAATCCAAATATGTCAATCATGGCAAACCAATCGAAAAGGAAGGGACGAATTTCCAGAGATTCCCCTTACGGTCTCAGTCAGGGCATTACGAAGAAGAATAAGGACTAGgcggccgc

**SQV-2 Constructs:**

NM1969, SQV-2/pGEX4T-1, included a N-terminus GST tagged full length SQV-2, amino acids: 1-330. Primers, #3379: 5’-CCGGAGGGGATCCGCGATGAGATTCTACCGAAC and #3380: 5’-ATAGTTTAGCGGCCGCTTATGGAATATTCGATCC, were used. The fragment was cloned into the BamHI/NotI site of the vector, pGEX4T-1.

NM2031, SQV-2/pVM, included a N-terminus His6 tagged full length SQV-2 from NM1969. NM1969 was digested with BamHI/NotI and cloned into the BamHI/NotI site of the vector, pVM.

NM1969:

ATGTTCGAAGATCGTTTATGTCATAAAACATATTTAAATGGTGATCATGTAACCCATCCTGACTTCATGTTGTATGACGCTCTTGATGTTGTTTTATACATGGACCCAATGTGCCTGGATGCGTTCCCAAAATTAGTTTGTTTTAAAAAACGTATTGAAGCTATCCCACAAATTGATAAGTACTTGAAATCCAGCAAGTATATAGCATGGCCTTTGCAGGGCTGGCAAGCCACGTTTGGTGGTGGCGACCATCCTCCAAAATCGGATCTGGTTCCGCGTGGATCCGCGATGAGATTCTACCGAACATATTTGCTCGTAGCTGGCGCTTTTTGCTCATTGTGCACACTTGCAGTAATATTCAATTGTGGATGGGATGATTCACCGCCAGCAACACCTTCAGCCATCAATGGCGGTGGCTCCAATGCTCCTTTAATCTCCTCTCCAACTAATCTTCCCGAAACATTTCTGTACATTTCAATTCTGACGTCACCAAACGAAACAGAACGACGTCAAAATGTCCGTGACACATGGTTCCGCCTATCAACTAAAGGACCGTCCGTTTTTATCGCAAAATTCGCCGTCGGAACGATGGGCCTCGCGGCCGAAGATCGTCGGTTGCTGGCCGAGGAAAATGAGAAATTCGGCGATTTGGCGCTTCTCGACCGCCATGAAGAGTCCTATGAGAGGCTGGCAAAGAAGACTTTGGCCTGTTTTGTACACGCTTTTGCCAATTTTAAATTCAAATTTTTCTTGAAGACCGACATCGACTCATTCGTCCGAATCACCCCACTAATCATAAATCTCAAACAAATTCAAGATCCAATGCTCTACTGGGGATTCCTAGATGGTCGAGCTAAACCATTCCGTAAAGGAAAATGGAAAGAACCCGAATGGAATCTGTGTGATCGTTATCTTCCATATCAACTTGGCGGTGGTTATGTGCTCTCTTATGAGCTCATTCGATTCTTGGCAATCAATGCCCAACTCTTCCGACACTATCGGAATGAAGATGTGTCGGTAGGCGCCTGGATAGGCGGCCTAGATGTTAAATATGTACATGATCCGAGATTTGATACCGAATGGAGATCCCGTGGATGTAATAATGAGTATTTAATTACTCATAAGCACACGGAGCAAGAGATGCAAGAGATGTTTGAAAATTTGAAGAAAACTGGAAAACTTTGTGCTAAAGAGTTCCAAAAACATCCATCCTACGTGTACGATTTCTCGAAAGCACCCAGCGAATGTTGTACAAGAGTCAACGGATCGAATATTCCATAAgcggccgc

NM2031:

ATGCGGGGTTCTCATCATCATCATCATCATGGTATGGCTAGCATGACTGGTGGACAGCAAATGGGTCGGGATCTGTACGACGATGACGATAAGGATCGATGGGGATCCGCGATGAGATTCTACCGAACATATTTGCTCGTAGCTGGCGCTTTTTGCTCATTGTGCACACTTGCAGTAATATTCAATTGTGGATGGGATGATTCACCGCCAGCAACACCTTCAGCCATCAATGGCGGTGGCTCCAATGCTCCTTTAATCTCCTCTCCAACTAATCTTCCCGAAACATTTCTGTACATTTCAATTCTGACGTCACCAAACGAAACAGAACGACGTCAAAATGTCCGTGACACATGGTTCCGCCTATCAACTAAAGGACCGTCCGTTTTTATCGCAAAATTCGCCGTCGGAACGATGGGCCTCGCGGCCGAAGATCGTCGGTTGCTGGCCGAGGAAAATGAGAAATTCGGCGATTTGGCGCTTCTCGACCGCCATGAAGAGTCCTATGAGAGGCTGGCAAAGAAGACTTTGGCCTGTTTTGTACACGCTTTTGCCAATTTTAAATTCAAATTTTTCTTGAAGACCGACATCGACTCATTCGTCCGAATCACCCCACTAATCATAAATCTCAAACAAATTCAAGATCCAATGCTCTACTGGGGATTCCTAGATGGTCGAGCTAAACCATTCCGTAAAGGAAAATGGAAAGAACCCGAATGGAATCTGTGTGATCGTTATCTTCCATATCAACTTGGCGGTGGTTATGTGCTCTCTTATGAGCTCATTCGATTCTTGGCAATCAATGCCCAACTCTTCCGACACTATCGGAATGAAGATGTGTCGGTAGGCGCCTGGATAGGCGGCCTAGATGTTAAATATGTACATGATCCGAGATTTGATACCGAATGGAGATCCCGTGGATGTAATAATGAGTATTTAATTACTCATAAGCACACGGAGCAAGAGATGCAAGAGATGTTTGAAAATTTGAAGAAAACTGGAAAACTTTGTGCTAAAGAGTTCCAAAAACATCCATCCTACGTGTACGATTTCTCGAAAGCACCCAGCGAATGTTGTACAAGAGTCAACGGATCGAATATTCCATAAgcggccgc

**AEX-6 Constructs:**

NM1123, RB27/pHO2d(-CXXC), included a C-terminus His6-Myc tagged AEX-6, amino acids 1-211. Primers, #1356: 5’-CATGGCCATGGGTGACTACGACTATCTC and #1357: 5’-TCGGGTCGACGCATAGGAAGAAGCGGCCG were used. The fragment was cloned into the NcoI/SalI site of the vector pH02d.

NM1117, RB27/pBHA, included full length AEX-6, amino acids 1-215. Primers, #1342: 5' GTCGGATCCGTATGGGTGACTACGACTATCTC and #1343: 5' TCGGCTGCAGTCAGCAATTTGCACAATAGGAAG, were used. The fragment was cloned into the BamHI/PstI site of the yeast shuttle vector pBHA [55].

NM1559, RB27/pGEX3, included a N-terminus His6 tagged AEX-6 from NM1117. NM1117 was digested with BamHI/NaeI and cloned into the BamHI/SmaI site of the vector pGEX3.

NM1123:

ccATGGGTGACTACGACTATCTCATCAAATTTCTCGCGCTCGGCGATTCGGGAGTCGGAAAAACGTCGTTTTTGCATCGTTACACGGATAACACGTTCACCGGACAATTCATTTCCACTGTTGGAATTGATTTTAAAGAGAAAAAAGTGGTCTACAAGAGTTCACGTGGCGGATTCGGTGGTCGTGGTCAACGAGTTTTACTTCAGTTATGGGACACTGCCGGACAAGAGAGATTCCGTTCTCTAACAACAGCGTTCTTCCGAGATGCAATGGGATTTATATTGATCTTCGATATTACAAATGAGCAATCATTTCTCAATATTCGAGACTGGCTATCACAATTGAAGGTACATGCCTATTGCGAGCAACCTGACATTATCATATGCGGTAACAAAGCAGACCTCGAAAATCGGCGTCAAGTCAGTACCGCCCGTGCAAAGCAGCTGGCTGATCAATTGGGGCTGCCGTACTTCGAGACGTCCGCCTGCACATCTACCAATGTGGAGAAATCTGTGGATTGTCTACTCGATTTGGTTATGCAAAGAATTCAACAATCTGTTGAGACGTCGTCTCTTCCGTTGTCAGAATGCCGCGGAGTCAGCTTGGACGGAGACCCATCGGCCGCTTCTTCCTATGCGTCGACAAGCTTGAATTCGGGCCACCATCACCACCATCACTAG

NM1117:

ggatccgtATGGGTGACTACGACTATCTCATCAAATTTCTCGCGCTCGGCGATTCGGGAGTCGGAAAAACGTCGTTTTTGCATCGTTACACGGATAACACGTTCACCGGACAATTCATTTCCACTGTTGGAATTGATTTTAAAGAGAAAAAAGTGGTCTACAAGAGTTCACGTGGCGGATTCGGTGGTCGTGGTCAACGAGTTTTACTTCAGTTATGGGACACTGCCGGACAAGAGAGATTCCGTTCTCTAACAACAGCGTTCTTCCGAGATGCAATGGGATTTATATTGATCTTCGATATTACAAATGAGCAATCATTTCTCAATATTCGAGACTGGCTATCACAATTGAAGGTACATGCCTATTGCGAGCAACCTGACATTATCATATGCGGTAACAAAGCAGACCTCGAAAATCGGCGTCAAGTCAGTACCGCCCGTGCAAAGCAGCTGGCTGATCAATTGGGGCTGCCGTACTTCGAGACGTCCGCCTGCACATCTACCAATGTGGAGAAATCTGTGGATTGTCTACTCGATTTGGTTATGCAAAGAATTCAACAATCTGTTGAGACGTCGTCTCTTCCGTTGTCAGAATGCCGCGGAGTCAGCTTGGACGGAGACCCATCGGCCGCTTCTTCCTATTGTGCAAATTGCTGActgcag

NM1559:

ATGTCCCCTATACTAGGTTATTGGAAAATTAAGGGCCTTGTGCAACCCACTCGACTTCTTTTGGAATATCTTGAAGAAAAATATGAAGAGCATTTGTATGAGCGCGATGAAGGTGATAAATGGCGAAACAAAAAGTTTGAATTGGGTTTGGAGTTTCCCAATCTTCCTTATTATATTGATGGTGATGTTAAATTAACACAGTCTATGGCCATCATACGTTATATAGCTGACAAGCACAACATGTTGGGTGGTTGTCCAAAAGAGCGTGCAGAGATTTCAATGCTTGAAGGAGCGGTTTTGGATATTAGATACGGTGTTTCGAGAATTGCATATAGTAAAGACTTTGAAACTCTCAAAGTTGATTTTCTTAGCAAGCTACCTGAAATGCTGAAAATGTTCGAAGATCGTTTATGTCATAAAACATATTTAAATGGTGATCATGTAACCCATCCTGACTTCATGTTGTATGACGCTCTTGATGTTGTTTTATACATGGACCCAATGTGCCTGGATGCGTTCCCAAAATTAGTTTGTTTTAAAAAACGTATTGAAGCTATCCCACAAATTGATAAGTACTTGAAATCCAGCAAGTATATAGCATGGCCTTTGCAGGGCTGGCAAGCCACGTTTGGTGGTGGCGACCATCCTCCAAAATCGGATCTGATCGAAGGTCGTGGGATCCGTATGGGTGACTACGACTATCTCATCAAATTTCTCGCGCTCGGCGATTCGGGAGTCGGAAAAACGTCGTTTTTGCATCGTTACACGGATAACACGTTCACCGGACAATTCATTTCCACTGTTGGAATTGATTTTAAAGAGAAAAAAGTGGTCTACAAGAGTTCACGTGGCGGATTCGGTGGTCGTGGTCAACGAGTTTTACTTCAGTTATGGGACACTGCCGGACAAGAGAGATTCCGTTCTCTAACAACAGCGTTCTTCCGAGATGCAATGGGATTTATATTGATCTTCGATATTACAAATGAGCAATCATTTCTCAATATTCGAGACTGGCTATCACAATTGAAGGTACATGCCTATTGCGAGCAACCTGACATTATCATATGCGGTAACAAAGCAGACCTCGAAAATCGGCGTCAAGTCAGTACCGCCCGTGCAAAGCAGCTGGCTGATCAATTGGGGCTGCCGTACTTCGAGACGTCCGCCTGCACATCTACCAATGTGGAGAAATCTGTGGATTGTCTACTCGATTTGGTTATGCAAAGAATTCAACAATCTGTTGAGACGTCGTCTCTTCCGTTGTCAGAATGCCGCGGAGTCAGCTTGGACGGAGACCCATCGGCCGCTTCTTCCTATTGTGCAAATTGCTGActgcagccaagctaattccgggcgaatttcttatgatttatgatttttattattaaataagttataaaaaaaataagtgtatacaaattttaaagtgactcttaggttttaaaacgaaaattcttattcttgagtaactctttcctgtaggtcaggttgctttctcaggtatagcatgaggtcgctcttattgaccacacctctaccggcatgccggg

**RAB-3 Constructs:**

NM759, pBHA-rab3, included a full length RAB-3b, amino acids 1-219. Primers, #510: 5’- GTCGGATCCGTATGGCGGCTGGCGGACAACCTCAAGGCGC and #511: 5’- TCGGCTGCAGTTAGCAATTGCATTGCTGTTG, were used. The fragment was cloned into the BamHI/PstI site of the yeast shuttle vector pBHA. [55].

NM1952, RAB-3/pRSETC, included a N-terminus His6 tagged RAB-3, amino acids 1-219. NM759, pBHA-rab3, was digested with BamHI/PstI and the *rab-3* insert was cloned into the vector pRSETC.

NM1255, pGEX-3/RB3, included a N-terminus GST tagged RAB-3b, amino acids 1-219. NM759, pBHA-rab3, was digested with BamHI/NaeI. This *rab-3* fragment was then cloned into the BamHI/SmaI site of the vector pGEX-3.

NM759:

GGGATCCGTATGGCGGCTGGCGGACAACCTCAAGGCGCTACACCGGGACAACCCGATCAGAACTTTGACTACATGTTCAAGCTCCTGATAATCGGAAATTCATCAGTTGGAAAAACATCATTCCTCTTCCGTTACTGTGATGATTCATTCACTTCTGCCTTCGTCTCTACTGTCGGAATCGATTTCAAAGTGAAAACTGTGTTCCGTGGAGACAAACGAGTCAAACTTCAAATCTGGGATACCGCCGGACAGGAGAGGTACCGTACCATCACCACCGCCTACTATCGTGGAGCAATGGGATTCATTCTGATGTATGACATCACTAATGAAGAGTCTTTTAATAGTGTTCAGGATTGGTGCACTCAAATCAAGACATACTCATGGGAAAATGCTCAAGTTGTTTTGGTTGGAAATAAATGTGATATGGACTCTGAAAGAGTTGTATCTATGGATAGGGGACGCCAACTTGCTGATCAACTTGGTTTGGAATTCTTCGAAACATCAGCCAAGGAGAACATTAATGTAAAGGCAGTTTTTGAGAAGTTGGTGGAGATTATTTGTGATAAGATGGCAGAGAGTTTGGATAAGGACCCACAGCAACAGCCAAAAGGACAGAAGCTCGAAGCGAATCCGACCCAAAAGCCTGCTCAACAGCAATGCAATTGCTAActgcag

NM1952:

ATGCGGGGTTCTCATCATCATCATCATCATGGTATGGCTAGCATGACTGGTGGACAGCAAATGGGTCGGGATCTGTACGACGATGACGATAAGGATCGATGGATCCGTATGGCGGCTGGCGGACAACCTCAAGGCGCTACACCGGGACAACCCGATCAGAACTTTGACTACATGTTCAAGCTCCTGATAATCGGAAATTCATCAGTTGGAAAAACATCATTCCTCTTCCGTTACTGTGATGATTCATTCACTTCTGCCTTCGTCTCTACTGTCGGAATCGATTTCAAAGTGAAAACTGTGTTCCGTGGAGACAAACGAGTCAAACTTCAAATCTGGGATACCGCCGGACAGGAGAGGTACCGTACCATCACCACCGCCTACTATCGTGGAGCAATGGGATTCATTCTGATGTATGACATCACTAATGAAGAGTCTTTTAATAGTGTTCAGGATTGGTGCACTCAAATCAAGACATACTCATGGGAAAATGCTCAAGTTGTTTTGGTTGGAAATAAATGTGATATGGACTCTGAAAGAGTTGTATCTATGGATAGGGGACGCCAACTTGCTGATCAACTTGGTTTGGAATTCTTCGAAACATCAGCCAAGGAGAACATTAATGTAAAGGCAGTTTTTGAGAAGTTGGTGGAGATTATTTGTGATAAGATGGCAGAGAGTTTGGATAAGGACCCACAGCAACAGCCAAAAGGACAGAAGCTCGAAGCGAATCCGACCCAAAAGCCTGCTCAACAGCAATGCAATTGCTAActgcag

NM1255:

ATGTCCCCTATACTAGGTTATTGGAAAATTAAGGGCCTTGTGCAACCCACTCGACTTCTTTTGGAATATCTTGAAGAAAAATATGAAGAGCATTTGTATGAGCGCGATGAAGGTGATAAATGGCGAAACAAAAAGTTTGAATTGGGTTTGGAGTTTCCCAATCTTCCTTATTATATTGATGGTGATGTTAAATTAACACAGTCTATGGCCATCATACGTTATATAGCTGACAAGCACAACATGTTGGGTGGTTGTCCAAAAGAGCGTGCAGAGATTTCAATGCTTGAAGGAGCGGTTTTGGATATTAGATACGGTGTTTCGAGAATTGCATATAGTAAAGACTTTGAAACTCTCAAAGTTGATTTTCTTAGCAAGCTACCTGAAATGCTGAAAATGTTCGAAGATCGTTTATGTCATAAAACATATTTAAATGGTGATCATGTAACCCATCCTGACTTCATGTTGTATGACGCTCTTGATGTTGTTTTATACATGGACCCAATGTGCCTGGATGCGTTCCCAAAATTAGTTTGTTTTAAAAAACGTATTGAAGCTATCCCACAAATTGATAAGTACTTGAAATCCAGCAAGTATATAGCATGGCCTTTGCAGGGCTGGCAAGCCACGTTTGGTGGTGGCGACCATCCTCCAAAATCGGATCTGATCGAAGGTCGTGGGATCCGTATGGCGGCTGGCGGACAACCTCAAGGCGCTACACCGGGACAACCCGATCAGAACTTTGACTACATGTTCAAGCTCCTGATAATCGGAAATTCATCAGTTGGAAAAACATCATTCCTCTTCCGTTACTGTGATGATTCATTCACTTCTGCCTTCGTCTCTACTGTCGGAATCGATTTCAAAGTGAAAACTGTGTTCCGTGGAGACAAACGAGTCAAACTTCAAATCTGGGATACCGCCGGACAGGAGAGGTACCGTACCATCACCACCGCCTACTATCGTGGAGCAATGGGATTCATTCTGATGTATGACATCACTAATGAAGAGTCTTTTAATAGTGTTCAGGATTGGTGCACTCAAATCAAGACATACTCATGGGAAAATGCTCAAGTTGTTTTGGTTGGAAATAAATGTGATATGGACTCTGAAAGAGTTGTATCTATGGATAGGGGACGCCAACTTGCTGATCAACTTGGTTTGGAATTCTTCGAAACATCAGCCAAGGAGAACATTAATGTAAAGGCAGTTTTTGAGAAGTTGGTGGAGATTATTTGTGATAAGATGGCAGAGAGTTTGGATAAGGACCCACAGCAACAGCCAAAAGGACAGAAGCTCGAAGCGAATCCGACCCAAAAGCCTGCTCAACAGCAATGCAATTGCTAActgcagccaagctaattccgggcgaatttcttatgatttatgatttttattattaaataagttataaaaaaaataagtgtatacaaattttaaagtgactcttaggttttaaaacgaaaattcttgttcttgagtaactctttcctgtaggtcaggttgctttctcaggtatagcatgaggtcgctcttattgaccacacctctaccggcatgccggg

**UNC-64 Constructs:**

NM679, pGEX2T-TX, included a N-terminus GST tagged version of UNC-64, amino acids 1-266. The primers, #170: 5’- CCGGGATCCATGACTAAGGACAGATTG and #143: 5’- CAAGATCTACTTCTTCCTTCGCGCCTTC, were used. The fragment was digested with BamHI/BglII and cloned into the BamHI site of the vector pGEX2T.

NM1801, UNC-64/pRSETA, included a N-terminus His6 tagged version of NM679. NM679 was digested with BamHI/SmaI, and the fragment was cloned into the BamHI/PvuII site of the vector pRSETA.

NM1899, UNC-64int/pHO4d, included the UNC-64 domain of NM1801. NM1801 was digested with XbaI/HindIII, and the fragment was cloned into the XbaI/HindIII site of the vector pHO4d.

NM1923, UNC-64/pHO4d, included a C-terminus His6-myc tagged version of NM679. The primers, #230: 5’- CCACAAATTGATAAGTACTTG and #3332: 5’-CGATTCAAGCTTATCTTCCTTCGCGCCTTCGA, were used. The fragment was cloned into the BseRI/HindIII site of the vector UNC-64int/pHO4d (NM1899).

NM679:

ATGTTCGAAGATCGTTTATGTCATAAAACATATTTAAATGGTGATCATGTAACCCATCCTGACTTCATGTTGTATGACGCTCTTGATGTTGTTTTATACATGGACCCAATGTGCCTGGATGCGTTCCCAAAATTAGTTTGTTTTAAAAAACGTATTGAAGCTATCCCACAAATTGATAAGTACTTGAAATCCAGCAAGTATATAGCATGGCCTTTGCAGGGCTGGCAAGCCACGTTTGGTGGTGGCGACCATCCTCCAAAATCGGATCTGGTTCCGCGTGGATCCATGACTAAGGACAGATTGTCCGCTTTAAAAGCGGCGCAGTCCGAGGATGAGCAGGACGATGATATGCACATGGACACCGGGAATGCACAATATATGGAGGAGTTTTTTGAGCAGGTTGAAGAGATTCGTGGAAGTGTGGATATTATTGCGAATAATGTTGAAGAGGTCAAGAAGAAGCATTCGGCAATTTTATCAAATCCAGTTAACGATCAGAAAACCAAAGAAGAACTCGACGAGTTGATGGCAGTTATCAAAAGAGCTGCGAATAAAGTGCGCGGCAAACTGAAATTGATCGAAAATGCCATTGATCACGACGAGCAAGGAGCCGGAAATGCGGATCTTCGAATTCGAAAAACTCAACACAGCACATTATCGAGACGATTCGTCGAAGTGATGACTGATTATAATAAGACACAGACTGATTATCGAGAGAGGTGTAAGGGACGAATTCAGAGACAACTCGATATTGCTGGAAAACAAGTCGGAGATGAGGATTTGGAGGAAATGATTGAGAGCGGAAATCCGGGAGTATTTACACAAGGAATCATCACAGATACCCAACAGGCAAAACAAACGCTAGCCGATATTGAAGCTCGTCACAATGATATCATGAAATTGGAAAGTTCAATTCGAGAGCTTCACGACATGTTCATGGATATGGCTATGCTTGTCGAATCTCAGGGAGAGATGGTTGATCGAATTGAGTACAATGTGGAGCACGCGAAAGAATTTGTTGATCGAGCAGTAGCTGATACGAAGAAAGCCGTTCAATATCAGTCGAAGGCGCGAAGGAAGAAGTAGatccccggg

NM1801:

ATGCGGGGTTCTCATCATCATCATCATCATGGTATGGCTAGCATGACTGGTGGACAGCAAATGGGTCGGGATCTGTACGACGATGACGATAAGGATCGATGGGGATCCATGACTAAGGACAGATTGTCCGCTTTAAAAGCGGCGCAGTCCGAGGATGAGCAGGACGATGATATGCACATGGACACCGGGAATGCACAATATATGGAGGAGTTTTTTGAGCAGGTTGAAGAGATTCGTGGAAGTGTGGATATTATTGCGAATAATGTTGAAGAGGTCAAGAAGAAGCATTCGGCAATTTTATCAAATCCAGTTAACGATCAGAAAACCAAAGAAGAACTCGACGAGTTGATGGCAGTTATCAAAAGAGCTGCGAATAAAGTGCGCGGCAAACTGAAATTGATCGAAAATGCCATTGATCACGACGAGCAAGGAGCCGGAAATGCGGATCTTCGAATTCGAAAAACTCAACACAGCACATTATCGAGACGATTCGTCGAAGTGATGACTGATTATAATAAGACACAGACTGATTATCGAGAGAGGTGTAAGGGACGAATTCAGAGACAACTCGATATTGCTGGAAAACAAGTCGGAGATGAGGATTTGGAGGAAATGATTGAGAGCGGAAATCCGGGAGTATTTACACAAGGAATCATCACAGATACCCAACAGGCAAAACAAACGCTAGCCGATATTGAAGCTCGTCACAATGATATCATGAAATTGGAAAGTTCAATTCGAGAGCTTCACGACATGTTCATGGATATGGCTATGCTTGTCGAATCTCAGGGAGAGATGGTTGATCGAATTGAGTACAATGTGGAGCACGCGAAAGAATTTGTTGATCGAGCAGTAGCTGATACGAAGAAAGCCGTTCAATATCAGTCGAAGGCGCGAAGGAAGAAGTAGatccccctg

NM1899:

tctagaaataattttgtttaactttaagaaggagatatacatATGCGGGGTTCTCATCATCATCATCATCATGGTATGGCTAGCATGACTGGTGGACAGCAAATGGGTCGGGATCTGTACGACGATGACGATAAGGATCGATGGGGATCCATGACTAAGGACAGATTGTCCGCTTTAAAAGCGGCGCAGTCCGAGGATGAGCAGGACGATGATATGCACATGGACACCGGGAATGCACAATATATGGAGGAGTTTTTTGAGCAGGTTGAAGAGATTCGTGGAAGTGTGGATATTATTGCGAATAATGTTGAAGAGGTCAAGAAGAAGCATTCGGCAATTTTATCAAATCCAGTTAACGATCAGAAAACCAAAGAAGAACTCGACGAGTTGATGGCAGTTATCAAAAGAGCTGCGAATAAAGTGCGCGGCAAACTGAAATTGATCGAAAATGCCATTGATCACGACGAGCAAGGAGCCGGAAATGCGGATCTTCGAATTCGAAAAACTCAACACAGCACATTATCGAGACGATTCGTCGAAGTGATGACTGATTATAATAAGACACAGACTGATTATCGAGAGAGGTGTAAGGGACGAATTCAGAGACAACTCGATATTGCTGGAAAACAAGTCGGAGATGAGGATTTGGAGGAAATGATTGAGAGCGGAAATCCGGGAGTATTTACACAAGGAATCATCACAGATACCCAACAGGCAAAACAAACGCTAGCCGATATTGAAGCTCGTCACAATGATATCATGAAATTGGAAAGTTCAATTCGAGAGCTTCACGACATGTTCATGGATATGGCTATGCTTGTCGAATCTCAGGGAGAGATGGTTGATCGAATTGAGTACAATGTGGAGCACGCGAAAGAATTTGTTGATCGAGCAGTAGCTGATACGAAGAAAGCCGTTCAATATCAGTCGAAGGCGCGAAGGAAGAAGTAGatccccctggtaccatggaattcgaagctt

NM1923:

ATGCGGGGTTCTCATCATCATCATCATCATGGTATGGCTAGCATGACTGGTGGACAGCAAATGGGTCGGGATCTGTACGACGATGACGATAAGGATCGATGGGGATCCATGACTAAGGACAGATTGTCCGCTTTAAAAGCGGCGCAGTCCGAGGATGAGCAGGACGATGATATGCACATGGACACCGGGAATGCACAATATATGGAGGAGTTTTTTGAGCAGGTTGAAGAGATTCGTGGAAGTGTGGATATTATTGCGAATAATGTTGAAGAGGTCAAGAAGAAGCATTCGGCAATTTTATCAAATCCAGTTAACGATCAGAAAACCAAAGAAGAACTCGACGAGTTGATGGCAGTTATCAAAAGAGCTGCGAATAAAGTGCGCGGCAAACTGAAATTGATCGAAAATGCCATTGATCACGACGAGCAAGGAGCCGGAAATGCGGATCTTCGAATTCGAAAAACTCAACACAGCACATTATCGAGACGATTCGTCGAAGTGATGACTGATTATAATAAGACACAGACTGATTATCGAGAGAGGTGTAAGGGACGAATTCAGAGACAACTCGATATTGCTGGAAAACAAGTCGGAGATGAGGATTTGGAGGAAATGATTGAGAGCGGAAATCCGGGAGTATTTACACAAGGAATCATCACAGATACCCAACAGGCAAAACAAACGCTAGCCGATATTGAAGCTCGTCACAATGATATCATGAAATTGGAAAGTTCAATTCGAGAGCTTCACGACATGTTCATGGATATGGCTATGCTTGTCGAATCTCAGGGAGAGATGGTTGATCGAATTGAGTACAATGTGGAGCACGCGAAAGAATTTGTTGATCGAGCAGTAGCTGATACGAAGAAAGCCGTTCAATATCAGTCGAAGGCGCGAAGGAAGATAAGCTTGAATTCGGGCCACCATCACCACCATCACGGCGAACAGAAACTGATCAGCGAAGAAGATCTGAACTAG

**UNC-29 Constructs:**

NM1456, pGEX-3X-unc29c, included a N-terminus tagged UNC-29, amino acids 348-431 (gift from M. Gendrel).

NM1456:

ATGTCCCCTATACTAGGTTATTGGAAAATTAAGGGCCTTGTGCAACCCACTCGACTTCTTTTGGAATATCTTGAAGAAAAATATGAAGAGCATTTGTATGAGCGCGATGAAGGTGATAAATGGCGAAACAAAAAGTTTGAATTGGGTTTGGAGTTTCCCAATCTTCCTTATTATATTGATGGTGATGTTAAATTAACACAGTCTATGGCCATCATACGTTATATAGCTGACAAGCACAACATGTTGGGTGGTTGTCCAAAAGAGCGTGCAGAGATTTCAATGCTTGAAGGAGCGGTTTTGGATATTAGATACGGTGTTTCGAGAATTGCATATAGTAAAGACTTTGAAACTCTCAAAGTTGATTTTCTTAGCAAGCTACCTGAAATGCTGAAAATGTTCGAAGATCGTTTATGTCATAAAACATATTTAAATGGTGATCATGTAACCCATCCTGACTTCATGTTGTATGACGCTCTTGATGTTGTTTTATACATGGACCCAATGTGCCTGGATGCGTTCCCAAAATTAGTTTGTTTTAAAAAACGTATTGAAGCTATCCCACAAATTGATAAGTACTTGAAATCCAGCAAGTATATAGCATGGCCTTTGCAGGGCTGGCAAGCCACGTTTGGTGGTGGCGACCATCCTCCAAAATCGGATCTGATCGAAGGTCGTGGGATCCCGAAATCGGCATCTGAGCGAAGCGCTGTCCGTTCTGGAATGGCCCAACTACCAGGAGTTGGACAGTTTACATTAAGCCCATCAGCTCATCATCCACTATGTCCATCAGCTGATGATCGAACAACTACAATTCGAAATACTGCATCAAATGAGACGTCTGCATATTATCCATTAAGTACTGATGCTCTACGAGCAATTGATGCAATTGAGTATATTACGGAACATTTGAAAAGAGACGAGCAACATAAATCGGGAATTCATCGTGActgactgacgatctgcctcgcgcgtttcggtgatgacggtgaaaacctctgacacatgcagctcccggagacggtcacagcttgtctgtaagcggatgccgggagcagacaagcccgtcagggcgcgtcagcgggtgttggcgggtgtcggggcgcagccat

**SAS-5 Constructs:**

NM1878, SAS-5/pGEX-4T, included a full length N-terminus GST tagged SAS-5, amino acids 1-404. Primers, # 3335: 5’- CCGGGATCCGCGATGAATAATTACGACGACTTACCCTGC and #3336: 5’-AAGAGGCGGCCGCCTCATTTCCTGCGAGCGTATTTTTC were used. The fragment was cloned into the BamHI/NotI site of the vector pGEX4T-1.

NM1961, SAS-5/pRSETA, included a full length N-terminus His6 tagged SAS-5 from NM1878. NM1878 was PCR’d with. primers, #3335 (SAS5F1: 5’- CCGGGATCCGCGATGAATAATTACGACGACTTACCCTGC and #3391: 5’-TCTTCAAGCTTATCATTTCCTGCGAGCGTATTTTTCACG. The fragment was cloned into the BamHI/HindIII site of the vector pRSETC.

NM1878:

ATGTTCGAAGATCGTTTATGTCATAAAACATATTTAAATGGTGATCATGTAACCCATCCTGACTTCATGTTGTATGACGCTCTTGATGTTGTTTTATACATGGACCCAATGTGCCTGGATGCGTTCCCAAAATTAGTTTGTTTTAAAAAACGTATTGAAGCTATCCCACAAATTGATAAGTACTTGAAATCCAGCAAGTATATAGCATGGCCTTTGCAGGGCTGGCAAGCCACGTTTGGTGGTGGCGACCATCCTCCAAAATCGGATCTGGTTCCGCGTGGATCCGCGATGAATAATTACGACGACTTACCCTGCTCTATTTACTTCAAAAAACCAACTGTCCAGGAGTTTGTGGATCAACCGAGAGTCTTCGAAGATTCTGAAGTCCCTGCCTTTCAAGAAGTTCTTCAGCTTCCACAAGAAAGGACAAAACCCCCAGTACCATCTACGCAGCCAATTGTCGCCGCTGTAGAAGTAGCCAAGAAAAAATCGTGTCTTTCGGCTCCGAAACCAAGAAAAGAGCCACCTTCGCATCCAGCGCTTCGCCAAAAAACAGTTGCGTTTGGAAAAACAGTTAACGTTTCCCAAACTGTCGAGGGAACATCTCGGAATTCAAAAAAAGTGCTTGCTTCAACAATGTCTGCCCAAAATACGACAACGACTGAAGAACAGGCAGCTGAAAACTGGCGGGACGCTATGAAAACAGAACTGCAAACGATACGAACAGAGATTCAAGAAGAAACGGCCCGGCGACAAGAAGAGCTCAATGCTCAAAATCTTGTTAAAATGCAAGAATTGATGTCAAACTTCTTTCAGAAGATAACGATTCCTAAACAGCAAGCGATCGAACCAGTTGAAAAAGACAAAGAAAATTTTCATGAGAGTCCTCGTCAATCAAGACAGCAGAAGCCAGCTAGTAAAATCGCTTCGGCACGAGAAGTTATCAAAAGAGACGGTGTTATACCTCCAGAAGCATTAACAATTATTGAGCAACGCCTTCGCTCGGACCCAATGTTTCGCCAACAAATTGATAATGTTCTAGCAGACGCTGAATGTGATGCTAACAGAGCTGCATATTCTCCACCACCACCAATGTCTGAAGTTAGATACGGATCTGGTGTGAATCCTGCTTTGATGAGAGAAACACTTACGGTAGAACGAAGTATTCGATATGACAACGGACTCGCTTCTATTGATTCTAGACAGTGGACAAATGAGAGACGTGATAATCGTGCTCCTGACTCATACCGTACCTACGAACCGGATCAACCATGTCACTCACTCTATCAAAAGGGTCAAAGTATTAGCTATTATCCGTCAGAAGCAGCTGGAAAAACGACAGCTAGAAACAATCGAACTGGATACTACGTGGAAGATTCATCAGATCATGAAGAGGATGTTGTAGTCAATAAACGGGGACAGAACTATCACGAGCAAGCGGTACCTGAGACTCCAGCTGAACGAGAACGCCGTATTCGTGAAAAATACGCTCGCAGGAAATGAggcggccgc

NM1961:

ATGCGGGGTTCTCATCATCATCATCATCATGGTATGGCTAGCATGACTGGTGGACAGCAAATGGGTCGGGATCTGTACGACGATGACGATAAGGATCGATGGGGATCCGCGATGAATAATTACGACGACTTACCCTGCTCTATTTACTTCAAAAAACCAACTGTCCAGGAGTTTGTGGATCAACCGAGAGTCTTCGAAGATTCTGAAGTCCCTGCCTTTCAAGAAGTTCTTCAGCTTCCACAAGAAAGGACAAAACCCCCAGTACCATCTACGCAGCCAATTGTCGCCGCTGTAGAAGTAGCCAAGAAAAAATCGTGTCTTTCGGCTCCGAAACCAAGAAAAGAGCCACCTTCGCATCCAGCGCTTCGCCAAAAAACAGTTGCGTTTGGAAAAACAGTTAACGTTTCCCAAACTGTCGAGGGAACATCTCGGAATTCAAAAAAAGTGCTTGCTTCAACAATGTCTGCCCAAAATACGACAACGACTGAAGAACAGGCAGCTGAAAACTGGCGGGACGCTATGAAAACAGAACTGCAAACGATACGAACAGAGATTCAAGAAGAAACGGCCCGGCGACAAGAAGAGCTCAATGCTCAAAATCTTGTTAAAATGCAAGAATTGATGTCAAACTTCTTTCAGAAGATAACGATTCCTAAACAGCAAGCGATCGAACCAGTTGAAAAAGACAAAGAAAATTTTCATGAGAGTCCTCGTCAATCAAGACAGCAGAAGCCAGCTAGTAAAATCGCTTCGGCACGAGAAGTTATCAAAAGAGACGGTGTTATACCTCCAGAAGCATTAACAATTATTGAGCAACGCCTTCGCTCGGACCCAATGTTTCGCCAACAAATTGATAATGTTCTAGCAGACGCTGAATGTGATGCTAACAGAGCTGCATATTCTCCACCACCACCAATGTCTGAAGTTAGATACGGATCTGGTGTGAATCCTGCTTTGATGAGAGAAACACTTACGGTAGAACGAAGTATTCGATATGACAACGGACTCGCTTCTATTGATTCTAGACAGTGGACAAATGAGAGACGTGATAATCGTGCTCCTGACTCATACCGTACCTACGAACCGGATCAACCATGTCACTCACTCTATCAAAAGGGTCAAAGTATTAGCTATTATCCGTCAGAAGCAGCTGGAAAAACGACAGCTAGAAACAATCGAACTGGATACTACGTGGAAGATTCATCAGATCATGAAGAGGATGTTGTAGTCAATAAACGGGGACAGAACTATCACGAGCAAGCGGTACCTGAGACTCCAGCTGAACGAGAACGCCGTATTCGTGAAAAATACGCTCGCAGGAAATGAtaagctt

**EEA-1 Constructs:**

NM1462, pET24b:EEA-1, included a C-terminus his6 tagged EEA-1, amino acids 973-1205 (gift from Barth Grant).

NM1783, EEA-1/pGEX4T-1, included N-terminus GST tagged EEA-1, amino acids: 973-1205. Primers #3172: 5’-AGGGGATCCGCGATGATTCAAGAGAAGGAAACTAC and #3173: 5’-GGCCGCTCGAGCGGTCATTTCTGGGAATCAGTGAA, were used. The fragment was cloned into the BamHI/XhoI site of the vector pGEX4T-1.

NM1783:

ATGTTCGAAGATCGTTTATGTCATAAAACATATTTAAATGGTGATCATGTAACCCATCCTGACTTCATGTTGTATGACGCTCTTGATGTTGTTTTATACATGGACCCAATGTGCCTGGATGCGTTCCCAAAATTAGTTTGTTTTAAAAAACGTATTGAAGCTATCCCACAAATTGATAAGTACTTGAAATCCAGCAAGTATATAGCATGGCCTTTGCAGGGCTGGCAAGCCACGTTTGGTGGTGGCGACCATCCTCCAAAATCGGATCTGGTTCCGCGTGGATCCGCGATGATTCAAGAGAAGGAAACTACGATTGCAAGGATGACATCCAGCAAAACACAATTTGAAGCCATGTTCGCCGACGTTCAACAGACACTTTCGAAAGAAATAAATGATAAAACTGAAGAAATTGAACGGCTCATGGAAAGAATTGATAGCCTTGAAAAGGTGAATCATTCAAGAATCGAAGAATTAGAGAGCAGATTGACTCAAAGAGAACGAGTCGTTGAATCTCTTGAAGCTGATTTGGCTGCAGTCAGAAATATCGAACAAGAGAAACTTGATGAGTTGCAGAAATTGAAAGAAGAATTTGATGAATTGAAAAAAGCTGAAACAATGTGGCAAGCAGAGAAAGATATGCTAATCGAGCGGTGTCTTGGCAGTGAAAGTGACATTGAATACGAGAAAGAGAGATCACAAGAGAATAAGAGAAGATTTGATGATGCTCTTTCAGCCATGCATGAACTTGGAAGAGCCAATCAGTCACTGCAGATTGATGCCGACCGTTACAGTTCTCGGAAATGGCTTGACGATGCAGAAGCAATTAATTGTACCGAATGTGGAAAGGTGTTTTCTCTGACAGTTCGAAAGCATCATTGCCGAGTTTGTGGAAAGATTTATTGCAATCCATGCAGTTCAAAATCAGTCCGAATTGCGAGTGCCAAGAATCCAGTCCGAGCCTGCAACACGTGCTTCACTGATTCCCAGAAATGAccgctcgag

**RME-2 Constructs:**

NM1451, pET24b:RME-2_EXT, included a N-terminus T7-tag and C-terminus His6-tagged RME-2, amino acids 181-659. PCR'd from the cDNA clone yk8d2 and cloned into the BamHI/XhoI site of the pET24b vector (gift from Barth Grant).

NM1815, RME-2/pGEX4T-1, included a N-terminus GST tagged RME-2 from NM1451. NM1451 was PCR’d with Primers, #3176: 5’-CCGGAATTCCCGATGTCCTCGACTGATTTTCACGATG and #3177: 5’-CCGCTCGAGCGGCCTCAGGTCTGGTAGAGTGAGTG. The fragment was cloned into the EcoRI/XhoI site of the vector, pGEX4T-1.

NM1815:

ATGTTCGAAGATCGTTTATGTCATAAAACATATTTAAATGGTGATCATGTAACCCATCCTGACTTCATGTTGTATGACGCTCTTGATGTTGTTTTATACATGGACCCAATGTGCCTGGATGCGTTCCCAAAATTAGTTTGTTTTAAAAAACGTATTGAAGCTATCCCACAAATTGATAAGTACTTGAAATCCAGCAAGTATATAGCATGGCCTTTGCAGGGCTGGCAAGCCACGTTTGGTGGTGGCGACCATCCTCCAAAATCGGATCTGGTTCCGCGTGGATCCCCGGAATTCCCGATGTCCTCGACTGATTTTCACGATGATGTTCATCTTGTCGATCCAACCTTTTTCGCTAATGAAGACAATAAGTGTCGGAGTGGATACACAATGTGCCATAGCGGAGACGTCTGCATACCTGACAGTTTTCTTTGTGACGGCGATCTAGATTGTGATGATGCTTCGGACGAGAAAAACTGCCAAACTAATGCTCCAAGCGAAGAAGAATATCTTTCTGGGCAAGCCGATCACATGCATTCGTGCTCAGCAGCAGGAATGTATTCTTGTGGAACAAAAGGATCCGAAATTGGCGTTTGTATTCCGATGAATGCCACGTGTAATGGGATCAAGGAGTGTCCACTAGGAGATGACGAGTCAAAACATTGCTCCGAATGTGCCAGAAAGCGATGTGACCACACATGTATGAACACTCCACACGGGGCTCGCTGCATTTGTCAAGAAGGATATAAGCTTGCCGATGACGGACTCACTTGCGAGGATGAAGATGAGTGTGCAACTCATGGGCACTTGTGCCAGCATTTCTGTGAAGATCGTTTGGGTTCCTTTGCATGCAAATGTGCCAACGGTTATGAGCTTGAAACGGATGGGCATTCTTGTAAATACGAGACAACCACTACGCCAGAAGGATATTTGTTCATCAGTCTTGGTGGAGAAGTTCGACAGATGCCATTGGCAGATTTCACCGATGGTTCAAATTACTCGCCGATTCAAAAGTTTGCTGGCCACGGAACCATCAGATCGATCGACTTCATGCATCGCAACAACAAAATGTTCATGTCAATTTCTGATGAGCACGGTGATCCAACTGGCGAATTGTCAGTGTCCGACAATGGATTGATGAGAGTTCTTCGAGAAAATGTCATTGGAGTGAGCAACGTGGCAGTCGACTGGATTGGTGGAAACGTTTTCTTCACACAAAAATCTCCATCTCCAAGCGCTGGGATTTCCATCTGCACAATGAGCGGAATGTTCTGTCGCCGAGTTATCGAAGGCAAAGAACAAGGACAATCCTATCGTGGTCTTGTTGTTCACCCGATGCGCGGTCTCATCATCTGGATCGATTCTTATCAGAAATATCATCGCATCATGATGGCTAATATGGATGGGTCTCAGGTCAGAATCCTTCTCGACAACAAGTTGGAAGTTCCATCAGCTCTTGCCATCGACTACATCCGCCACGATGTCTATTTTGGAGATGTTGAACGTCAGTTGATCGAAAGAGTCAATATCGACACGAAAGAGCGCCGCGTAGTGATTTCGAACGGAGTTCATCATCCGTATGACATGGCTTACTTCAATGGTTTCCTATACTGGGCAGATTGGGGAAGCGAGTCATTAAAGGTTCAAGAGATGACCCATCATCATTCGAGTCCTCAAGTCATCCATACTTTCAATCGTTATCCATATGGTATTGCTGTCAATCACTCACTCTACCAGACCTGAggccgctcgag

**RME-8 Constructs:**

NM1449, pET24b:RME-8, included a N-terminus T7-tag and C-terminus His6 tagged RME-8, amino acids 2065 to 2256. It was PCR’d from the cDNA, yk212c7, and cloned into the BamHI/XhoI site of the pET24b vector (gift from Barth Grant).

NM1812, RME-8/pGEX4T-1, includes a N-terminus GST tagged RME-8 from NM1449. NM1449 was PCR’d with primers, #3174: 5’-AGGGGATCCGCGATGGCCTTTGTTGAACTGGTCCGTC and #3175: 5’-GGCCGCTCGAGCGGTCATTGATGCATTGGTGGTGGCT. The fragment was cloned into the BamHI/XhoI site of the vector pGEX4T-1.

NM1812:

ATGTTCGAAGATCGTTTATGTCATAAAACATATTTAAATGGTGATCATGTAACCCATCCTGACTTCATGTTGTATGACGCTCTTGATGTTGTTTTATACATGGACCCAATGTGCCTGGATGCGTTCCCAAAATTAGTTTGTTTTAAAAAACGTATTGAAGCTATCCCACAAATTGATAAGTACTTGAAATCCAGCAAGTATATAGCATGGCCTTTGCAGGGCTGGCAAGCCACGTTTGGTGGTGGCGACCATCCTCCAAAATCGGATCTGGTTCCGCGTGGATCCGCGATGGCCTTTGTTGAACTGGTCCGTCATCATCCGAATACTGCTGATCAATTGCCATCTCAAGGATATCTACCACAGTTCTGCACTGCAATGTGTCTTCAAAACACATCTGCGTCTCGTTCAGCAATTCTAATTCTTCAAGAATTGAGCGAAAATCAATTCTGTTGTGATGCTCTTTCTCAGCTGCCATGTATTGATGGAATAATGAAATCAATGAAGAATCAACCAAGTTTGATGAGAGAATCGGCGCACGCTTTAAAATGTCTCATGAAGAGAAATACAGGAGAATTGGCTCAACAAATGCTCTCATGTGGAATGGTTCCATATTTGTTACAAGTTTTGGATAGTTCAATGAATGGAGTATCAAACGGAGCAGCTGCTCGTGCCGAGATTGTTGACGCATTAAAAAGCGCAATTTTAGATCTGAAAGTTGGTCAGAAGATTGCTGAGATTTTAGACAAGAGTCCCGTCTGGGCACAGTTTAAAGATCAACGACATGATTTATTCTTGCCAGA

AGCGAGAACTCAAGCTATTACAGGAGGTCCAACTGGTGTTGCTGGATATCTAACAGAAGGAATGTTCAATCCACCACCAATGAGCAATCAGCCACCACCAATGCATCAATGAccgctcgag

**PAR-1 Constructs:**

NM1606, PAR-1/pGEX-3X, included a N-terminus GST tagged domain of PAR-1, amino acids 454-674 (gift from K. Kemphues and M. Beers).

NM1622, PAR-1/pRSETA, included a N-terminus His6 tagged domain of PAR-1 from NM1606. Primers, #2829: 5’-AGGGATCCGCGATGCAACTCGGATTCAACAAAG and #2474: 5’- GGCATCCGCTTACAGACAAG, were used. The fragment was cloned into the BamHI/EcoRI site of the vector pRSETA.

NM1622:

ATGCGGGGTTCTCATCATCATCATCATCATGGTATGGCTAGCATGACTGGTGGACAGCAAATGGGTCGGGATCTGTACGACGATGACGATAAGGATCGATGGGGATCCGCGATGCAACTCGGATTCAACAAAGCCGCCATTTTGGAAAGCGTCGAGAAGGAAAAGTTTGAAGATATTCATGCAACGTATTTGCTTTTAGGAGAACGAAAATCTGATATGGATGCCAGTGAAATCACAATGGCCCAATCACTGCTCAGCCATTCGTCAATCAATGTTTCGTCGTCTCTGGGTCAGCACCCGGCCGGTGTTATCACTAGGGAGCATGTGACGTCATCATCAGCGTCTGGAAGCTCTGCTTCACCATCCCGATACTCTCGATCCTCAGCAACAGCCACAGGTGCATCAATCACTGCAGGATCAGCGCTCGCATCGGCTGCGAATGCTCAGAAACATCAGCAATCATCGGCAGCTCCATCGTCAGGATCTTCGTCGTCGAGGAGAAGCTCGCAAAACGATGCGGCAGCGACGGCGGCTGGTGGTACTGTAGTGATGAGTGGAACAAGGCATGGTGGAGTTCAGATGAGAGCTCAGCCGACGAGTCGGCAAGCGACAATCAGTTTATTGCAACCGCCGAGTTATAAGCCTTCCTCCAACACCACGCAAATTGCTCAAATTCCGCCACTATTCAATAGAAACTCAACGGCAACCTCATCAGCTGCTCAACCTTCAACAGGAATCACCGGTACTCGAAAGATTGCTGATCCAAAAGGACGAATTCGAAGCTTGATCCGGCTGCTAACAAAGCCCGAAAGGAAGCTGAGTTGGCTGCTGCCACCGCTGAGCAATAACTAG

**PAR-6 Constructs:**

NM1607, pQEPAR6, included a N-terminus His6 tagged domain of PAR-6, amino acids 1-309, cloned into the BamHI/SalI site of the vector pQE32 (gift from K Kemphues and M. Beers).

His6-PAR-6 fusion protein (gift from K. Kemphues and M. Beers).

NM1806, PAR-6/pGEX4T-1, included a N-terminus GST tagged full length domain of PAR-6, amino acids 1-309 amino acids. Primers, #3167: 5’-AGGGGATCCGCGATGTCCTACAACGGCTCCTAC and #3168: 5’-AAGAGGCGGCCGCCTCAGTCCTCTCCACTGTCC, were used. The fragment was cloned into the BamHI/NotI site of the vector pGEX4T-1.

NM1806:

ATGTTCGAAGATCGTTTATGTCATAAAACATATTTAAATGGTGATCATGTAACCCATCCTGACTTCATGTTGTATGACGCTCTTGATGTTGTTTTATACATGGACCCAATGTGCCTGGATGCGTTCCCAAAATTAGTTTGTTTTAAAAAACGTATTGAAGCTATCCCACAAATTGATAAGTACTTGAAATCCAGCAAGTATATAGCATGGCCTTTGCAGGGCTGGCAAGCCACGTTTGGTGGTGGCGACCATCCTCCAAAATCGGATCTGGTTCCGCGTGGATCCGCGATGTCCTACAACGGCTCCTACCATCAAAATCATCATTCAACACTTCAAGTCAAATCGAAATTTGATTCTGAATGGCGTCGTTTCTCAATACCAATGCATTCTGCGTCTGGTGTCTCTTACGATGGGTTCCGGAGCCTTGTCGAGAAGCTTCATCACCTGGAAAGTGTCCAATTCACACTGTGCTACAACTCGACTGGCGGTGATCTTCTACCAATCACAAATGACGATAACCTCCGAAAATCGTTCGAATCGGCTCGCCCACTGCTCCGTTTGCTCATTCAACGCCGCGGTGAATCATGGGAAGAGAAATATGGTTATGGTACAGACTCTGATAAACGATGGAAGGGAATTTCATCACTTATGGCACAAAAACCACCGAAACGCAGTTATTCAATCTCGAATCCCGAAGATTTTCGACAGGTCTCTGCAATCATCGACGTGGACATTGTGCCAGAAGCTCATCGCCGTGTCCGTCTCTGCAAGCACGGACAAGAACGACCACTTGGATTCTATATTCGTGACGGAACATCGGTTCGAGTGACAGAACGAGGCGTCGTCAAAGTTTCAGGAATTTTCATTTCCCGGCTTGTCGATGGTGGGCTCGCCGAGTCCACAGGCCTTCTTGGTGTTAATGACGAGGTGCTCGAGGTGAATGGAATTGAGGTTCTCGGAAAGACGCTGGATCAGGTCACTGATATGATGGTCGCCAATGCTCATAACTTGATTATTACTGTAAAACCTGCCAACCAACGAAACACACTGTCACGAGGACCGTCACAACAAGGAACACCGAACGCCAGTGAGATGTCGGCCGCCACCGCAGCCGCTACAGGTGGAATTCAGCGCCCGATGAAAATGAACGGAAGCTCCGACGGCAGTTATCATCCGAAACAGCACGACGCAAATGATTCGGACAGTGGAGAGGACTGAggcggccgc

**GLR-1 Constructs:**

NM1658, GLR-1/pRSETA, included a N-terminus His6 tagged GLR-1, amino acids 871-962. Primers #2895: 5’-AGGGGATCCGCGATGTTGGCTGCACTCGGG and #2896: 5’-ATGGTACCCCTCAGACAGCTGTGTTGTAGA were used. The fragment was cloned into the BamHI/KpnI site of the vector pRSETA.

NM1794, GLR-1/pGEX4T-1, included a N-terminus GST tagged GLR-1, amino acids 871-96. Primers, #3170: 5’-AGGGGATCCGCGATGTTGGCTGCACTCGGG and #3171: 5’-GGCCGCTCGAGCGGTCAGACAGCTGTGTTGTA were used. The fragment was cloned into the BamHI/XhoI site of the vector pGEX4T-1.

NM1658:

ATGCGGGGTTCTCATCATCATCATCATCATGGTATGGCTAGCATGACTGGTGGACAGCAAATGGGTCGGGATCTGTACGACGATGACGATAAGGATCGATGGGGATCCGCGATGTTGGCTGCACTCGGGGAATTCTTGTATCGAAGTAGGATTGAAGCGAGGAAATCTAATTCCAATTCTATGGTGGCGAATTTTGCGAAAAATTTGAAAAGTGCATTGTCATCTCAATTAAGATTATCAGTCGAAGGAGGTGCAGTTGCACAACCAGGATCTCAATCTCATAATGCAATTAGAAGACAACAGGTAGCTGCATTCTTGCCTGCAAATGAAAAAGAAGCTTTCAATAATGTGGATCGACCTGCAAACACTCTCTACAACACAGCTGTCTGAggggtacc

NM1794:

ATGTTCGAAGATCGTTTATGTCATAAAACATATTTAAATGGTGATCATGTAACCCATCCTGACTTCATGTTGTATGACGCTCTTGATGTTGTTTTATACATGGACCCAATGTGCCTGGATGCGTTCCCAAAATTAGTTTGTTTTAAAAAACGTATTGAAGCTATCCCACAAATTGATAAGTACTTGAAATCCAGCAAGTATATAGCATGGCCTTTGCAGGGCTGGCAAGCCACGTTTGGTGGTGGCGACCATCCTCCAAAATCGGATCTGGTTCCGCGTGGATCCGCGATGTTGGCTGCACTCGGGGAATTCTTGTATCGAAGTAGGATTGAAGCGAGGAAATCTAATTCCAATTCTATGGTGGCGAATTTTGCGAAAAATTTGAAAAGTGCATTGTCATCTCAATTAAGATTATCAGTCGAAGGAGGTGCAGTTGCACAACCAGGATCTCAATCTCATAATGCAATTAGAAGACAACAGGTAGCTGCATTCTTGCCTGCAAATGAAAAAGAAGCTTTCAATAATGTGGATCGACCTGCAAACACTCTCTACAACACAGCTGTCTGAccgctcgag

**EAT-4 Constructs:**

NM1698, EAT-4/pRSETA, included a N-terminus His6 tagged EAT-4, amino acids 508-576. Primers, #2974: 5’-GGCCGCTCGAGCGGATGTGGTCGAATAAGGAATTG and #2975: 5’-CCGGAATTCCGTCACTACCACTGCTGATAATGCGG were used. The fragment was cloned into the XhoI/EcoRI site of the vector pRSETA.

NM1785, EAT-4/pGEX4T-1, included a N-terminus GST tagged EAT-4, amino acids 508-576. Primers, #3157: 5’-CCGGAATTCCGGATGTGGTCGAATAAGGAATTG and #3158: 5’-CCGCTCGAGCGGCCCTACCACTGCTGATAATG were used. The fragment was cloned into the EcoRI/XhoI site of the vector pGEX4T-1.

NM1698:

ATGCGGGGTTCTCATCATCATCATCATCATGGTATGGCTAGCATGACTGGTGGACAGCAAATGGGTCGGGATCTGTACGACGATGACGATAAGGATCGATGGGGATCCGAGCTCGAGCGGATGTGGTCGAATAAGGAATTGGTGAATAAAACTGGAATCAATGGAACTGGATACGGAGCTGCTGAGACTACATTCACACAGTTACCAGCCGGTGTTGATTCTTCTTATCAAGCTCAGGCGGCTCCAGCTCCTGGAACCAACCCGTTCGCTTCAGCTTGGGATGAACATGGATCCTCTGGAGTTGTGGAAAATCCGCATTATCAGCAGTGGTAGTGAcggaattc

NM1785:

ATGTTCGAAGATCGTTTATGTCATAAAACATATTTAAATGGTGATCATGTAACCCATCCTGACTTCATGTTGTATGACGCTCTTGATGTTGTTTTATACATGGACCCAATGTGCCTGGATGCGTTCCCAAAATTAGTTTGTTTTAAAAAACGTATTGAAGCTATCCCACAAATTGATAAGTACTTGAAATCCAGCAAGTATATAGCATGGCCTTTGCAGGGCTGGCAAGCCACGTTTGGTGGTGGCGACCATCCTCCAAAATCGGATCTGGTTCCGCGTGGATCCCCGGAATTCCGGATGTGGTCGAATAAGGAATTGGTGAATAAAACTGGAATCAATGGAACTGGATACGGAGCTGCTGAGACTACATTCACACAGTTACCAGCCGGTGTTGATTCTTCTTATCAAGCTCAGGCGGCTCCAGCTCCTGGAACCAACCCGTTCGCTTCAGCTTGGGATGAACATGGATCCTCTGGAGTTGTGGAAAATCCGCATTATCAGCAGTGGTAGggccgctcgag

**TRAP-2 Constructs:**

NM1905, TRAP-2/SUMOpST, included a N-terminus His6-Myc tagged TRAP-2, amino acids 1-188. Primers, #3325: 5’-GGCCGCTCGAGCGATGAAATTCTCACTCTTCGCCCTA and #3324: 5’-AAGAGGCGGCCGCCTTAAGTAGACTTCTTCTTGAT, were used. The fragment was cloned into the XhoI/NotI site of the vector pSUMOpST.

NM1958, TRAP-2/pRSETC, included a N-terminus His6 tagged TRAP-2 from NM1905. NM1905 was digested with XhoI/HindIII and cloned into the XhoI/HindIII site of the vector pRSETC.

NM1908, TRAP-2/pGEX4T-1, included a N-terminus GST tagged TRAP-2, amino acids 1-188. Primers, #3323: 5’-CCGGAATTCCCGATGAAATTCTCACTCTTCGCCCTA and #3324: 5’-AAGAGGCGGCCGCCTTAAGTAGACTTCTTCTTGAT, were used. The fragment was cloned into the EcoRI/NotI site of the vector pGEX4T-1.

NM1905:

ATGGGCAGCAGCCATCATCATCATCATCACGGCAGCGGCCTGGTGCCGCGCGGCAGCGCTAGCATGTCGGACTCAGAAGTCAATCAAGAAGCTAAGCCAGAGGTCAAGCCAGAAGTCAAGCCTGAGACTCACATCAATTTAAAGGTGTCCGATGGATCTTCAGAGATCTTCTTCAAGATCAAAAAGACCACTCCTTTAAGAAGGCTGATGGAAGCGTTCGCTAAAAGACAGGGTAAGGAAATGGACTCCTTAAGATTCTTGTACGACGGTATTAGAATTCAAGCTGATCAGACCCCTGAAGATTTGGACATGGAGGATAACGATATTATTGAGGCTCACAGAGAACAGATTGGTGGTAGCGACCAAGTGGGCGGTCCGGGATCCGGAGCTCGAGCGATGAAATTCTCACTCTTCGCCCTACTTTTTGTAGTGGTCAGCTGCGTTGATGTAGGAACTCAGACCAGGGATGCCTTCATCCTTGCCCACAAGCAGCCACTCTCCACATATGCCGTGGAGAACATGGACTTTGTCTTGGAATACGGACTCTACAACGTCGGAGACAAGCCAGCGCAGAAGGTCACCATCGACGATCGTCACTCGTTCCCAACCAACTCGTTCGACATCGTCAAGGGACTTCTCTTCGTGCACTTCGAGCAGATCCCAGCCGGAAGCAACGTGACCCACTCAGTTGTCATCCGCCCAAGAGCCTTCGGATTCTTCAACTACACCGCCGCCCAGGTCACTTACTACACCGACAACGAGAACCATCACGTCACCCTTACCAACACTCCTGGAGAAGGATACATCTACCGCCAACGCGAGTACGACAGACGATTTGCTCCAAAGTACACCTACTTCCTCGTCTTCTTCCTCATCGTCGCCCCAACCACTCTCGGATCCTTCCTTCTCTTCCAACAATCAAAGGCTCGCTTCCCAAATGTTATCAAGAAGAAGTCTACTTAAggcggccgc

NM1958:

ATGCGGGGTTCTCATCATCATCATCATCATGGTATGGCTAGCATGACTGGTGGACAGCAAATGGGTCGGGATCTGTACGACGATGACGATAAGGATCGATGGATCCGACCTCGAGCGATGAAATTCTCACTCTTCGCCCTACTTTTTGTAGTGGTCAGCTGCGTTGATGTAGGAACTCAGACCAGGGATGCCTTCATCCTTGCCCACAAGCAGCCACTCTCCACATATGCCGTGGAGAACATGGACTTTGTCTTGGAATACGGACTCTACAACGTCGGAGACAAGCCAGCGCAGAAGGTCACCATCGACGATCGTCACTCGTTCCCAACCAACTCGTTCGACATCGTCAAGGGACTTCTCTTCGTGCACTTCGAGCAGATCCCAGCCGGAAGCAACGTGACCCACTCAGTTGTCATCCGCCCAAGAGCCTTCGGATTCTTCAACTACACCGCCGCCCAGGTCACTTACTACACCGACAACGAGAACCATCACGTCACCCTTACCAACACTCCTGGAGAAGGATACATCTACCGCCAACGCGAGTACGACAGACGATTTGCTCCAAAGTACACCTACTTCCTCGTCTTCTTCCTCATCGTCGCCCCAACCACTCTCGGATCCTTCCTTCTCTTCCAACAATCAAAGGCTCGCTTCCCAAATGTTATCAAGAAGAAGTCTACTTAAggcggccgcatagtacctaagtactagacaagctt

NM1908:

ATGTTCGAAGATCGTTTATGTCATAAAACATATTTAAATGGTGATCATGTAACCCATCCTGACTTCATGTTGTATGACGCTCTTGATGTTGTTTTATACATGGACCCAATGTGCCTGGATGCGTTCCCAAAATTAGTTTGTTTTAAAAAACGTATTGAAGCTATCCCACAAATTGATAAGTACTTGAAATCCAGCAAGTATATAGCATGGCCTTTGCAGGGCTGGCAAGCCACGTTTGGTGGTGGCGACCATCCTCCAAAATCGGATCTGGTTCCGCGTGGATCCCCGGAATTCCCGATGAAATTCTCACTCTTCGCCCTACTTTTTGTAGTGGTCAGCTGCGTTGATGTAGGAACTCAGACCAGGGATGCCTTCATCCTTGCCCACAAGCAGCCACTCTCCACATATGCCGTGGAGAACATGGACTTTGTCTTGGAATACGGACTCTACAACGTCGGAGACAAGCCAGCGCAGAAGGTCACCATCGACGATCGTCACTCGTTCCCAACCAACTCGTTCGACATCGTCAAGGGACTTCTCTTCGTGCACTTCGAGCAGATCCCAGCCGGAAGCAACGTGACCCACTCAGTTGTCATCCGCCCAAGAGCCTTCGGATTCTTCAACTACACCGCCGCCCAGGTCACTTACTACACCGACAACGAGAACCATCACGTCACCCTTACCAACACTCCTGGAGAAGGATACATCTACCGCCAACGCGAGTACGACAGACGATTTGCTCCAAAGTACACCTACTTCCTCGTCTTCTTCCTCATCGTCGCCCCAACCACTCTCGGATCCTTCCTTCTCTTCCAACAATCAAAGGCTCGCTTCCCAAATGTTATCAAGAAGAAGTCTACTTAAggcggccgc

**CUP-5 Constructs:**

NM1915, CUP-5/SUMOpST, included a N-terminus His6-Myc tagged CUP-5d, amino acids 550-668. Primers, # 3326: 5’-AGGGGATCCGCGATGGATGCATATGAAGTCGTC and #3327: 5’-AAGAGGCGGCCGCCCTATACATTCGATGGCCTAG, were used. The fragment was cloned into the BamHI/NotI site of the vector pSUMOpST.

NM1992, CUP-5/pRSETA, included a N-terminus His6 tagged CUP-5d from NM1915. NM1915 was digested with BamHI/HindIII and cloned into the BamHI/HindIII site of the vector pRSETA.

NM1890, CUP-5/pGEX4T-1, included a N-terminus GST tagged CUP-5d, amino acids 550-668. Primers, #3326: 5’-AGGGGATCCGCGATGGATGCATATGAAGTCGTC and #3327: 5’-AAGAGGCGGCCGCCCTATACATTCGATGGCCTAG, were used. The fragment was cloned into the BamHI/NotI site of the vector pGEX4T-1.

NM1915:

ATGGGCAGCAGCCATCATCATCATCATCACGGCAGCGGCCTGGTGCCGCGCGGCAGCGCTAGCATGTCGGACTCAGAAGTCAATCAAGAAGCTAAGCCAGAGGTCAAGCCAGAAGTCAAGCCTGAGACTCACATCAATTTAAAGGTGTCCGATGGATCTTCAGAGATCTTCTTCAAGATCAAAAAGACCACTCCTTTAAGAAGGCTGATGGAAGCGTTCGCTAAAAGACAGGGTAAGGAAATGGACTCCTTAAGATTCTTGTACGACGGTATTAGAATTCAAGCTGATCAGACCCCTGAAGATTTGGACATGGAGGATAACGATATTATTGAGGCTCACAGAGAACAGATTGGTGGTAGCGACCAAGTGGGCGGTCCGGGATCCGCGATGGATGCATATGAAGTCGTCAAGGATCGCTACTCAGACGGTCTTCGAGCCATCGAAAAACGTGGATGTCTTCGCGATTTCGTGGAATCGAATCCACCGCCGTCGGAACTCGGCTCACCGACGACTCGATCCGCCTACGCACCGTCTAATCTGCTCAATCTGGCGGCTGGCAACGACTCGAGTAGAGCTCTTCGTGCTCTTCATGCCATTGACAATGCTCGCGATTGGCTGAGCAGCTTAAGAGATGGAACTCGTTTTCAATCATTCTCCAACCCGCTGAACGACTCAAATGAAGATGTATTAGCCGACCAAAATGCCAATGGAAACGATCCTCACAATTCTAGGCCATCGAATGTATAGggcggccg

NM1992:

ATGCGGGGTTCTCATCATCATCATCATCATGGTATGGCTAGCATGACTGGTGGACAGCAAATGGGTCGGGATCTGTACGACGATGACGATAAGGATCGATGGGGATCCGCGATGGATGCATATGAAGTCGTCAAGGATCGCTACTCAGACGGTCTTCGAGCCATCGAAAAACGTGGATGTCTTCGCGATTTCGTGGAATCGAATCCACCGCCGTCGGAACTCGGCTCACCGACGACTCGATCCGCCTACGCACCGTCTAATCTGCTCAATCTGGCGGCTGGCAACGACTCGAGTAGAGCTCTTCGTGCTCTTCATGCCATTGACAATGCTCGCGATTGGCTGAGCAGCTTAAGAGATGGAACTCGTTTTCAATCATTCTCCAACCCGCTGAACGACTCAAATGAAGATGTATTAGCCGACCAAAATGCCAATGGAAACGATCCTCACAATTCTAGGCCATCGAATGTATAGggcggccgcatagtacctaagtactagacaagctt

NM1890:

ATGTTCGAAGATCGTTTATGTCATAAAACATATTTAAATGGTGATCATGTAACCCATCCTGACTTCATGTTGTATGACGCTCTTGATGTTGTTTTATACATGGACCCAATGTGCCTGGATGCGTTCCCAAAATTAGTTTGTTTTAAAAAACGTATTGAAGCTATCCCACAAATTGATAAGTACTTGAAATCCAGCAAGTATATAGCATGGCCTTTGCAGGGCTGGCAAGCCACGTTTGGTGGTGGCGACCATCCTCCAAAATCGGATCTGGTTCCGCGTGGATCCGCGATGGATGCATATGAAGTCGTCAAGGATCGCTACTCAGACGGTCTTCGAGCCATCGAAAAACGTGGATGTCTTCGCGATTTCGTGGAATCGAATCCACCGCCGTCGGAACTCGGCTCACCGACGACTCGATCCGCCTACGCACCGTCTAATCTGCTCAATCTGGCGGCTGGCAACGACTCGAGTAGAGCTCTTCGTGCTCTTCATGCCATTGACAATGCTCGCGATTGGCTGAGCAGCTTAAGAGATGGAACTCGTTTTCAATCATTCTCCAACCCGCTGAACGACTCAAATGAAGATGTATTAGCCGACCAAAATGCCAATGGAAACGATCCTCACAATTCTAGGCCATCGAATGTATAGggcggccgc

**ELKS-1 Constructs:**

NM1195, CAST/pRSTB, included a N-terminus His6 tagged ELKS-1, amino acids 711-836. Primers, #1379: 5’-CGGGATCCCGGGATCAAATAATCAGAG and #1380: 5’-CCGGAATTCCTCAGGCCCAAATTCCGTCA, were used. The fragment was digested with SmaI/EcoRI and cloned into the PvuII/EcoRI site of the vector pRSETB.

NM1955, CAST/pGEX4T, included a N-terminus GST tagged ELKS-1, amino acids 711-836. Primers #3390: 5’-CCGGGATCCATGCGGGGCGATCAAATAATCAGAG and #3389: 5’-GGCCGCTCGAGCGTCAGGCCCAAATTCCGTCAGC, were used. The fragment was cloned into the BamHI/XhoI site of the vector pGEX4T-1.

NM1966, ELKS-1/pRSETA, included a N-terminus His6 tagged ELKS-1 from NM1955. NM1955 was cloned into the BamHI/XhoI site of the vector pRSETA.

NM1195:

ATGCGGGGTTCTCATCATCATCATCATCATGGTATGGCTAGCATGACTGGTGGACAGCAAATGGGTCGGGATCTGTACGACGATGACGATAAGGATCCGAGCTCGAGATCTGCAGGGGATCAAATAATCAGAGCCATCGAAACTGAGCGTCGCCAGCATTTGGAGCAACTTTTCCAGCTAAAACAAGAAGCCTTGCTAGCTGCAATTTCTGAAAAAGATACCCACCTGGCGCTTCTCGAAAAATCACGTGGGCCTCGTGACGAAATTGAGACGATCCGGAGGCATAAGGACGCGTTGATTCGGAAGTTGAAGCAGGAAAATGAGCGACGCGTTCTGGTCTCCCACCCGGACCCTGTAATATCGATGAACGCGATGGCCAGTGTGATTCCAGGAGCCCCGTTACCGGCTCCAGTGATTCCCGGTACCATTGGCATTCCACAACACTCTCAGCACCCGCCACAGGTAGATCACGACGATGCTGACGGAATTTGGGCCTGAggaattc

NM1955:

ATGTTCGAAGATCGTTTATGTCATAAAACATATTTAAATGGTGATCATGTAACCCATCCTGACTTCATGTTGTATGACGCTCTTGATGTTGTTTTATACATGGACCCAATGTGCCTGGATGCGTTCCCAAAATTAGTTTGTTTTAAAAAACGTATTGAAGCTATCCCACAAATTGATAAGTACTTGAAATCCAGCAAGTATATAGCATGGCCTTTGCAGGGCTGGCAAGCCACGTTTGGTGGTGGCGACCATCCTCCAAAATCGGATCTGGTTCCGCGTGGATCCATGCGGGGCGATCAAATAATCAGAGCCATCGAAACTGAGCGTCGCCAGCATTTGGAGCAACTTTTCCAGCTAAAACAAGAAGCCTTGCTAGCTGCAATTTCTGAAAAAGATACCCACCTGGCGCTTCTCGAAAAATCACGTGGGCCTCGTGACGAAATTGAGACGATCCGGAGGCATAAGGACGCGTTGATTCGGAAGTTGAAGCAGGAAAATGAGCGACGCGTTCTGGTCTCCCACCCGGACCCTGTAATATCGATGAACGCGATGGCCAGTGTGATTCCAGGAGCCCCGTTACCGGCTCCAGTGATTCCCGGTACCATTGGCATTCCACAACACTCTCAGCACCCGCCACAGGTAGATCACGACGATGCTGACGGAATTTGGGCCTGAcgctcgag

NM1966:

ATGCGGGGTTCTCATCATCATCATCATCATGGTATGGCTAGCATGACTGGTGGACAGCAAATGGGTCGGGATCTGTACGACGATGACGATAAGGATCGATGGGGATCCATGCGGGGCGATCAAATAATCAGAGCCATCGAAACTGAGCGTCGCCAGCATTTGGAGCAACTTTTCCAGCTAAAACAAGAAGCCTTGCTAGCTGCAATTTCTGAAAAAGATACCCACCTGGCGCTTCTCGAAAAATCACGTGGGCCTCGTGACGAAATTGAGACGATCCGGAGGCATAAGGACGCGTTGATTCGGAAGTTGAAGCAGGAAAATGAGCGACGCGTTCTGGTCTCCCACCCGGACCCTGTAATATCGATGAACGCGATGGCCAGTGTGATTCCAGGAGCCCCGTTACCGGCTCCAGTGATTCCCGGTACCATTGGCATTCCACAACACTCTCAGCACCCGCCACAGGTAGATCACGACGATGCTGACGGAATTTGGGCCTGAcgctcgag

**TAC-1 Constructs:**

GST-TAC-1 fusion protein (gift from P. Gönczy).

REFERENCES

1. Zorio DA, Lea K, Blumenthal T (1997) Cloning of Caenorhabditis U2AF65: an alternatively spliced RNA containing a novel exon. Mol Cell Biol 17: 946-953.

2. MacMorris M, Brocker C, Blumenthal T (2003) UAP56 levels affect viability and mRNA export in Caenorhabditis elegans. Rna 9: 847-857.

3. Gama-Carvalho M, Krauss RD, Chiang L, Valcarcel J, Green MR, et al. (1997) Targeting of U2AF65 to sites of active splicing in the nucleus. J Cell Biol 137: 975-987.

4. Jeziorski MC, Greenberg RM, Anderson PA (2000) The molecular biology of invertebrate voltage-gated Ca(2+) channels. J Exp Biol 203: 841-856.

5. Lee JH, Cribbs LL, Perez-Reyes E (1999) Cloning of a novel four repeat protein related to voltage-gated sodium and calcium channels. FEBS Lett 445: 231-236.

6. Yeh E, Ng S, Zhang M, Bouhours M, Wang Y, et al. (2008) A putative cation channel, NCA-1, and a novel protein, UNC-80, transmit neuronal activity in C. elegans. PLoS Biol 6: e55.

7. Zetka MC, Kawasaki I, Strome S, Muller F (1999) Synapsis and chiasma formation in Caenorhabditis elegans require HIM-3, a meiotic chromosome core component that functions in chromosome segregation. Genes Dev 13: 2258-2270.

8. Hollingsworth NM, Goetsch L, Byers B (1990) The HOP1 gene encodes a meiosis-specific component of yeast chromosomes. Cell 61: 73-84.

9. Hwang HY, Olson SK, Brown JR, Esko JD, Horvitz HR (2003) The Caenorhabditis elegans genes sqv-2 and sqv-6, which are required for vulval morphogenesis, encode glycosaminoglycan galactosyltransferase II and xylosyltransferase. J Biol Chem 278: 11735-11738.

10. Wang H, Spang A, Sullivan MA, Hryhorenko J, Hagen FK (2005) The terminal phase of cytokinesis in the Caenorhabditis elegans early embryo requires protein glycosylation. Mol Biol Cell 16: 4202-4213.

11. Mahoney TR, Liu Q, Itoh T, Luo S, Hadwiger G, et al. (2006) Regulation of synaptic transmission by RAB-3 and RAB-27 in Caenorhabditis elegans. Mol Biol Cell 17: 2617-2625.

12. Tsuboi T (2009) Molecular mechanism of attachment process of dense-core vesicles to the plasma membrane in neuroendocrine cells. Neurosci Res 63: 83-88.

13. Nonet ML, Staunton JE, Kilgard MP, Fergestad T, Hartwieg E, et al. (1997) Caenorhabditis elegans rab-3 mutant synapses exhibit impaired function and are partially depleted of vesicles. J Neurosci 17: 8061-8073.

14. Saifee O, Wei L, Nonet ML (1998) The Caenorhabditis elegans unc-64 locus encodes a syntaxin that interacts genetically with synaptobrevin. Mol Biol Cell 9: 1235-1252.

15. Fleming JT, Squire MD, Barnes TM, Tornoe C, Matsuda K, et al. (1997) Caenorhabditis elegans levamisole resistance genes lev-1, unc-29, and unc-38 encode functional nicotinic acetylcholine receptor subunits. J Neurosci 17: 5843-5857.

16. Gally C, Eimer S, Richmond JE, Bessereau JL (2004) A transmembrane protein required for acetylcholine receptor clustering in Caenorhabditis elegans. Nature 431: 578-582.

17. Delattre M, Leidel S, Wani K, Baumer K, Bamat J, et al. (2004) Centriolar SAS-5 is required for centrosome duplication in C. elegans. Nat Cell Biol 6: 656-664.

18. Delattre M, Canard C, Gonczy P (2006) Sequential protein recruitment in C. elegans centriole formation. Curr Biol 16: 1844-1849.

19. Mu FT, Callaghan JM, Steele-Mortimer O, Stenmark H, Parton RG, et al. (1995) EEA1, an early endosome-associated protein. EEA1 is a conserved alpha-helical peripheral membrane protein flanked by cysteine "fingers" and contains a calmodulin-binding IQ motif. J Biol Chem 270: 13503-13511.

20. Sato M, Sato K, Fonarev P, Huang CJ, Liou W, et al. (2005) Caenorhabditis elegans RME-6 is a novel regulator of RAB-5 at the clathrin-coated pit. Nat Cell Biol 7: 559-569.

21. Andrews R, Ahringer J (2007) Asymmetry of early endosome distribution in C. elegans embryos. PLoS One 2: e493.

22. Roggo L, Bernard V, Kovacs AL, Rose AM, Savoy F, et al. (2002) Membrane transport in Caenorhabditis elegans: an essential role for VPS34 at the nuclear membrane. Embo J 21: 1673-1683.

23. Grant B, Hirsh D (1999) Receptor-mediated endocytosis in the Caenorhabditis elegans oocyte. Mol Biol Cell 10: 4311-4326.

24. Paupard MC, Miller A, Grant B, Hirsh D, Hall DH (2001) Immuno-EM localization of GFP-tagged yolk proteins in C. elegans using microwave fixation. J Histochem Cytochem 49: 949-956.

25. Zhang Y, Grant B, Hirsh D (2001) RME-8, a conserved J-domain protein, is required for endocytosis in Caenorhabditis elegans. Mol Biol Cell 12: 2011-2021.

26. Schroeder LK, Kremer S, Kramer MJ, Currie E, Kwan E, et al. (2007) Function of the Caenorhabditis elegans ABC transporter PGP-2 in the biogenesis of a lysosome-related fat storage organelle. Mol Biol Cell 18: 995-1008.

27. Hurd DD, Kemphues KJ (2003) PAR-1 is required for morphogenesis of the Caenorhabditis elegans vulva. Dev Biol 253: 54-65.

28. Guo S, Kemphues KJ (1995) par-1, a gene required for establishing polarity in C. elegans embryos, encodes a putative Ser/Thr kinase that is asymmetrically distributed. Cell 81: 611-620.

29. Cheeks RJ, Canman JC, Gabriel WN, Meyer N, Strome S, et al. (2004) C. elegans PAR proteins function by mobilizing and stabilizing asymmetrically localized protein complexes. Curr Biol 14: 851-862.

30. Hung TJ, Kemphues KJ (1999) PAR-6 is a conserved PDZ domain-containing protein that colocalizes with PAR-3 in Caenorhabditis elegans embryos. Development 126: 127-135.

31. Totong R, Achilleos A, Nance J (2007) PAR-6 is required for junction formation but not apicobasal polarization in C. elegans embryonic epithelial cells. Development 134: 1259-1268.

32. Munro E, Nance J, Priess JR (2004) Cortical flows powered by asymmetrical contraction transport PAR proteins to establish and maintain anterior-posterior polarity in the early C. elegans embryo. Dev Cell 7: 413-424.

33. Hart AC, Sims S, Kaplan JM (1995) Synaptic code for sensory modalities revealed by *C. elegans* GLR-1 glutamate receptor. Nature 378: 82-85.

34. Maricq AV, Peckol E, Driscoll M, Bargmann CI (1995) Mechanosensory signalling in *C. elegans* mediated by the GLR-1 glutamate receptor. Nature: 78-81.

35. Kass J, Jacob TC, Kim P, Kaplan JM (2001) The EGL-3 proprotein convertase regulates mechanosensory responses of Caenorhabditis elegans. J Neurosci 21: 9265-9272.

36. Rongo C, Whitfield CW, Rodal A, Kim SK, Kaplan JM (1998) LIN-10 is a shared component of the polarized protein localization pathways in neurons and epithelia. Cell 94: 751-759.

37. Zheng Y, Brockie P, Mellem JE, Madsen DM, Maricq AV (1999) Neuronal control of Locomotion in C. elegans is Modified by a Dominant Mutation in the GLR-1 ionotropic Glutamate receptor. Neuron 24: 347-361.

38. Brockie PJ, Madsen DM, Zheng Y, Mellem J, Maricq AV (2001) Differential expression of glutamate receptor subunits in the nervous system of Caenorhabditis elegans and their regulation by the homeodomain protein UNC-42. J Neurosci 21: 1510-1522.

39. Chang HC, Rongo C (2005) Cytosolic tail sequences and subunit interactions are critical for synaptic localization of glutamate receptors. J Cell Sci 118: 1945-1956.

40. Bellocchio EE, Reimer RJ, Fremeau RT, Jr., Edwards RH (2000) Uptake of glutamate into synaptic vesicles by an inorganic phosphate transporter. Science 289: 957-960.

41. Lee RYN, Sawin ER, Chalfie M, Horvitz HR, Avery L (1999) EAT-4, a homolog of a mammalian sodium-dependent inorganic phosphate cotransporter, is necessary for glutamatergic neurotransmission in *Caenorhabditis elegans.* J Neurosci 19: 159-167.

42. Bellocchio EE, Hu H, Pohorille A, Chan J, Pickel VM, et al. (1998) The localization of the brain-specific inorganic phosphate transporter suggests a specific presynaptic role in glutamatergic transmission. J Neurosci 18: 8648-8659.

43. Lee D, Jung S, Ryu J, Ahnn J, Ha I (2008) Human vesicular glutamate transporters functionally complement EAT-4 in C. elegans. Mol Cells 25: 50-54.

44. Rolls MM, Hall DH, Victor M, Stelzer EH, Rapoport TA (2002) Targeting of rough endoplasmic reticulum membrane proteins and ribosomes in invertebrate neurons. Mol Biol Cell 13: 1778-1791.

45. Fares H, Greenwald I (2001) Regulation of endocytosis by CUP-5, the Caenorhabditis elegans mucolipin-1 homolog. Nat Genet 28: 64-68.

46. Hersh BM, Hartwieg E, Horvitz HR (2002) The Caenorhabditis elegans mucolipin-like gene cup-5 is essential for viability and regulates lysosomes in multiple cell types. Proc Natl Acad Sci U S A 99: 4355-4360.

47. Zeevi DA, Frumkin A, Bach G (2007) TRPML and lysosomal function. Biochim Biophys Acta 1772: 851-858.

48. Pryor PR, Reimann F, Gribble FM, Luzio JP (2006) Mucolipin-1 is a lysosomal membrane protein required for intracellular lactosylceramide traffic. Traffic 7: 1388-1398.

49. Treusch S, Knuth S, Slaugenhaupt SA, Goldin E, Grant BD, et al. (2004) Caenorhabditis elegans functional orthologue of human protein h-mucolipin-1 is required for lysosome biogenesis. Proc Natl Acad Sci U S A 101: 4483-4488.

50. Deken SL, Vincent R, Hadwiger G, Liu Q, Wang ZW, et al. (2005) Redundant localization mechanisms of RIM and ELKS in Caenorhabditis elegans. J Neurosci 25: 5975-5983.

51. Bellanger JM, Gonczy P (2003) TAC-1 and ZYG-9 form a complex that promotes microtubule assembly in C. elegans embryos. Curr Biol 13: 1488-1498.

52. Margittai M, Otto H, Jahn R (1999) A stable interaction between syntaxin 1a and synaptobrevin 2 mediated by their transmembrane domains. FEBS Lett 446: 40-44.

53. Fasshauer D, Otto H, Eliason WK, Jahn R, Brunger AT (1997) Structural changes are associated with soluble N-ethylmaleimide-sensitive fusion protein attachment protein receptor complex formation. J Biol Chem 272: 28036-28041.

54. Bartel P, Chien CT, Sternglanz R, Fields S (1993) Elimination of false positives that arise in using the two-hybrid system. Biotechniques 14: 920-924.

55. Gracheva EO, Hadwiger G, Nonet ML, Richmond JE (2008) Direct interactions between C. elegans RAB-3 and Rim provide a mechanism to target vesicles to the presynaptic density. Neurosci Lett 444: 137-142.
