# Supplementary material for: A Monoclonal Antibody Toolkit for C. elegans
Source: PLoS One. 2010 Apr 13;5(4):e10161. doi: 10.1371/journal.pone.0010161 (PMC2854156; doi:10.1371/journal.pone.0010161)
Supplement: File S2 — Supplemental methods detailing construction of immunogen expressing clones. (0.13 MB DOC) [file pone.0010161.s002.doc]

**A Monoclonal Antibody Toolkit for *C. elegans***

G. Hadwiger, S. Dour, S. Arur, P. Fox, and M.L. Nonet

**supplemental methods**

**Vectors used for Protein Expression:**

pRSETA His6-tag vector (Invitrogen, Carlsbad, CA)

pHO4d His6-Myc-tag vector[1 ; gift from P. Hanson]

pET24b His6-tag vector (Invitrogen, Carlsbad, CA)

NM1986, pVM, is a modification of the vector, pRSETA, with a linker: 5’- CCATGGAATTCGGCGGCCGCATAGTACCTAAGTACTAGACAAGCTT, cloned into the NcoI/HindIII site of pRSETA.

pET SUMO-His6-tag vector (Invitrogen; Carlsbad, CA)

NM1840, pSUMOpST, is a modification of the vector, pET SUMO (Invitrogen) with a linker: 5’-AGCGACCAAGTGGGCGGTCCGGGATCCGGAGCTCGAGATCTGCAGCTGGTACCATGGAATTCGGCGGCCGCATAGTACCTAAGTACTA, cloned into the vector, pET SUMO by TOPO TA cloning (Invitrogen).

pGEX2T, pGEX4T-1and pGEX6P-1 GST-tag vectors GE Healthcare, Piscataway, NJ)

**Fusion Protein Expression Constructs:**

**SNB-1 Constructs:**

NM645, pHO4SB(61-62), included a C-terminus His6-myc tagged domain of SNB-1, amino acids 1-86. The primers, #316: 5’-GTTGCCATGGACGCTCAAGGAGATG and #317: 5’-GGCGAATTCTTGATGTTCTTCCACCAATACTTG, were used. The fragment was cloned into the NcoI/EcoRI site of the vector pHO4d.

NM1809, SNB-1/pGEX4T-1, included a N-terminus GST tagged version of SNB-1, amino acids 1-86. The primers, #316: 5’-AGGGGATCCGCGATGGACGCTCAAGGAGATGCC and #317: 5’-GGCGAATTCTTGATGTTCTTC CACCAATACTTG, were used. The fragment was cloned into the BamHI/EcoRI site of the vector pGEX4T-1.

NM645 insert sequence:

ccATGGACGCTCAAGGAGATGCCGGCGCACAGGGAGGAAGTCAGGGCGGACCACGACCATCCAACAAACGTCTCCAGCAGACACAAGCTCAGGTTGATGAAGTCGTCGGAATCATGAAGGTGAACGTGGAAAAAGTTTTGGAGCGTGATCAGAAGTTGTCTCAACTGGACGACCGAGCTGACGCTCTCCAGGAAGGTGCTTCACAATTTGAGAAATCTGCGGCCACCCTGAAGCGCAAGTATTGGTGGAAGAACATCAAGAATTCGGGCCACCATCACCACCATCACGGCGAACAGAAACTGATCAGCGAAGAAGATCTGAACTAG

NM1809 insert sequence:

ATGTTCGAAGATCGTTTATGTCATAAAACATATTTAAATGGTGATCATGTAACCCATCCTGACTTCATGTTGTATGACGCTCTTGATGTTGTTTTATACATGGACCCAATGTGCCTGGATGCGTTCCCAAAATTAGTTTGTTTTAAAAAACGTATTGAAGCTATCCCACAAATTGATAAGTACTTGAAATCCAGCAAGTATATAGCATGGCCTTTGCAGGGCTGGCAAGCCACGTTTGGTGGTGGCGACCATCCTCCAAAATCGGATCTGGTTCCGCGTGGATCCGCGATGGACGCTCAAGGAGATGCCGGCGCACAGGGAGGAAGTCAGGGCGGACCACGACCATCCAACAAACGTCTCCAGCAGACACAAGCTCAGGTTGATGAAGTCGTCGGAATCATGAAGGTGAACGTGGAAAAAGTTTTGGAGCGTGATCAGAAGTTGTCTCAACTGGACGACCGAGCTGACGCTCTCCAGGAAGGTGCTTCACAATTTGAGAAATCTGCGGCCACCCTGAAGCGCAAGTATTGGTGGAAGAACATCAAGAATTCATCGTGA

**UNC-10 Constructs:**

NM822, pRSETA/rimz, included a N-terminus His6-tagged zinc finger domain of UNC-10, amino acids 1-144. Primers, #513: 5’-CAGGGATCCATGGACGATCCGTCGATGATG and #514: 5’-CTGGAATTCGAGCCGCTAATTTTCGGC, were used. The fragment was cloned into the BamHI/EcoRI site of the vector pRSETA.

NM1761, RMZ/pGEX2T, was a N-terminus GST-tagged version of NM822. NM822 was digested with BamHI/EcoRI, and the fragment was cloned into the vector pGEX2T.

NM822 insert sequence:

ATGCGGGGTTCTCATCATCATCATCATCATGGTATGGCTAGCATGACTGGTGGACAGCAAATGGGTCGGGATCTGTACGACGATGACGATAAGGATCGATGGGGATCCATGGACGATCCGTCGATGATGCCGGATTTATCCCATTTATCTGCAGAAGAACGTGAAATAATAGAAAATGTCTTCAAACGACAAAAAGATGAGGAGGCAAAAGAAACACAAATATCACAAAAAGCATCTGAAGAATTGTCAGAGTTAGACAAACAGATCACGGAGCGGAAAGAAACATCGAAAAAGCTGGTTGGCACACAAGATGACGCGATCTGTCAAATCTGTCAGAAGACCAAATTTGCGGACGGAATTGGCCACAAGTGCTTTTATTGTCAATTGCGTAGCTGCGCCAGATGTGGAGGTAGAGCACAGAGCAAAAATAAGGCAATCTGGGCTTGTTCGTTGTGCCAGAAACGTCAACAAATTCTTGCCAAAACAGGCAAATGGTTCCAACCCGAAGAGCAGCCGCAGCCGAAAATTAGCGGCTCGGAATTCGAAGCTTGA

NM1761insert sequence:

ATGTTCGAAGATCGTTTATGTCATAAAACATATTTAAATGGTGATCATGTAACCCATCCTGACTTCATGTTGTATGACGCTCTTGATGTTGTTTTATACATGGACCCAATGTGCCTGGATGCGTTCCCAAAATTAGTTTGTTTTAAAAAACGTATTGAAGCTATCCCACAAATTGATAAGTACTTGAAATCCAGCAAGTATATAGCATGGCCTTTGCAGGGCTGGCAAGCCACGTTTGGTGGTGGCGACCATCCTCCAAAATCGGATCTGGTTCCGCGTGGATCCATGGACGATCCGTCGATGATGCCGGATTTATCCCATTTATCTGCAGAAGAACGTGAAATAATAGAAAATGTCTTCAAACGACAAAAAGATGAGGAGGCAAAAGAAACACAAATATCACAAAAAGCATCTGAAGAATTGTCAGAGTTAGACAAACAGATCACGGAGCGGAAAGAAACATCGAAAAAGCTGGTTGGCACACAAGATGACGCGATCTGTCAAATCTGTCAGAAGACCAAATTTGCGGACGGAATTGGCCACAAGTGCTTTTATTGTCAATTGCGTAGCTGCGCCAGATGTGGAGGTAGAGCACAGAGCAAAAATAAGGCAATCTGGGCTTGTTCGTTGTGCCAGAAACGTCAACAAATTCTTGCCAAAACAGGCAAATGGTTCCAACCCGAAGAGCAGCCGCAGCCGAAAATTAGCGGCTCGGAATTCATCGTGACTGACTGA

**DLG-1 Constructs:**

NM1642, DLG-1/pRSETA, included a N-terminus His6 tagged domain of DLG-1, amino acids: 204-593. The primers, #2814: 5’-AGGGATCCGTCTTGGAGAAAGGTCAC and #2815: 5’- ATGGTACCCTCTTGTGGTCTGTACTG, were used. The fragment was cloned into the BamHI/NcoI site of the vector pRSETA.

NM1763, DLG-1/pGEX2T, included a N-terminus GST tagged domain of DLG-1 from NM1642. NM1642 was digested with BamHI/EcoRI and the fragment was cloned into the BamHI/EcoRI site of the vector pGEX-2T.

NM1642 insert sequence:

ATGCGGGGTTCTCATCATCATCATCATCATGGTATGGCTAGCATGACTGGTGGACAGCAAATGGGTCGGGATCTGTACGACGATGACGATAAGGATCGATGGGGATCCGTCTTGGAGAAAGGTCACACCGGCCTTGGATTCTCGATTACTGGAGGTATGGACCAACCAACAGAAGACGGAGATACTTCTATCTATGTCACCAATATTATTGAAGGAGGTGCCGCACTTGCTGATGGGCGTATGAGAAAGAATGATATTATCACTGCAGTCAACAATACAAACTGTGAAAATGTGAAGCATGAGGTTGCAGTCAACGCATTGAAAAGCTCCGGAAACGTTGTTTCGTTGAGTTTGAAACGGCGTAAAGATGAAGCCTTTCTTCCAATTGGAGGAAACTTTGGCGGGTCGACTTCTTACCTCAGATCAGGAGTCACTCCATCAGTGAGCGCCGGCAACTTGCAACACGCAATTCATTCTCCATCGGCTCCGATTCATCCACCACCGCCACCACCAGTTCATCACGGATCATTGAGCCAATTATCTGTTGGCCAATATCGTTCCACACGACCGAATACATCCGTCATTGATCTGGTTAAGGGAGCACGTGGACTTGGTTTCTCTATTGCTGGTGGTCAAGGAAACGAGCACGTTAAAGGAGATACCGATATCTATGTGACGAAAATCATTGAGGAGGGAGCAGCTGAATTAGACGGAAGATTAAGAGTCGGAGATAAGATTCTTGAAGTTGATCATCACTCTTTGATTAACACGACTCATGAAAATGCGGTCAATGTTCTCAAAAACACTGGAAATCGTGTTCGACTTCTTATTCAGCAAGGAACTGGGGCAATTTTCAATGACTCGGCGAGCCAACAATTCATGCCAACTACCCCAATTCTCAGACCTAGCTCCGTTCAAGACTACAACCGCTCTCAAATGGGATCTCAAAGTCATTTGTCTTACGGAGGACCACTGAACACCAGCTACAGTTCACAAGCCCCCATCGCAATTCCACTCGAACCCCGTCCAGTACAACTTGTAAAGGGCCAAAATGGACTTGGATTCAACATTGTTGGCGGAGAAGACAATGAGCCCATCTACATCAGTTTTGTTCTCCCAGGAGGTGTTGCTGATCTTAGTGGAAACGTGAAGACTGGAGACGTTCTTCTTGAAGTGAACGGAGTTGTTTTGAGAAATGCAACCCATAAGGAAGCAGCTGAAGCATTGAGAAACGCTGGAAATCCCGTTTATCTCACTCTTCAGTACAGACCACAAGAGGGTACCATGGAATTCGAAGCTTGA

NM1763 insert sequence:

ATGTTCGAAGATCGTTTATGTCATAAAACATATTTAAATGGTGATCATGTAACCCATCCTGACTTCATGTTGTATGACGCTCTTGATGTTGTTTTATACATGGACCCAATGTGCCTGGATGCGTTCCCAAAATTAGTTTGTTTTAAAAAACGTATTGAAGCTATCCCACAAATTGATAAGTACTTGAAATCCAGCAAGTATATAGCATGGCCTTTGCAGGGCTGGCAAGCCACGTTTGGTGGTGGCGACCATCCTCCAAAATCGGATCTGGTTCCGCGTGGATCCGTCTTGGAGAAAGGTCACACCGGCCTTGGATTCTCGATTACTGGAGGTATGGACCAACCAACAGAAGACGGAGATACTTCTATCTATGTCACCAATATTATTGAAGGAGGTGCCGCACTTGCTGATGGGCGTATGAGAAAGAATGATATTATCACTGCAGTCAACAATACAAACTGTGAAAATGTGAAGCATGAGGTTGCAGTCAACGCATTGAAAAGCTCCGGAAACGTTGTTTCGTTGAGTTTGAAACGGCGTAAAGATGAAGCCTTTCTTCCAATTGGAGGAAACTTTGGCGGGTCGACTTCTTACCTCAGATCAGGAGTCACTCCATCAGTGAGCGCCGGCAACTTGCAACACGCAATTCATTCTCCATCGGCTCCGATTCATCCACCACCGCCACCACCAGTTCATCACGGATCATTGAGCCAATTATCTGTTGGCCAATATCGTTCCACACGACCGAATACATCCGTCATTGATCTGGTTAAGGGAGCACGTGGACTTGGTTTCTCTATTGCTGGTGGTCAAGGAAACGAGCACGTTAAAGGAGATACCGATATCTATGTGACGAAAATCATTGAGGAGGGAGCAGCTGAATTAGACGGAAGATTAAGAGTCGGAGATAAGATTCTTGAAGTTGATCATCACTCTTTGATTAACACGACTCATGAAAATGCGGTCAATGTTCTCAAAAACACTGGAAATCGTGTTCGACTTCTTATTCAGCAAGGAACTGGGGCAATTTTCAATGACTCGGCGAGCCAACAATTCATGCCAACTACCCCAATTCTCAGACCTAGCTCCGTTCAAGACTACAACCGCTCTCAAATGGGATCTCAAAGTCATTTGTCTTACGGAGGACCACTGAACACCAGCTACAGTTCACAAGCCCCCATCGCAATTCCACTCGAACCCCGTCCAGTACAACTTGTAAAGGGCCAAAATGGACTTGGATTCAACATTGTTGGCGGAGAAGACAATGAGCCCATCTACATCAGTTTTGTTCTCCCAGGAGGTGTTGCTGATCTTAGTGGAAACGTGAAGACTGGAGACGTTCTTCTTGAAGTGAACGGAGTTGTTTTGAGAAATGCAACCCATAAGGAAGCAGCTGAAGCATTGAGAAACGCTGGAAATCCCGTTTATCTCACTCTTCAGTACAGACCACAAGAGGGTACCATGGAATTCATCGTGACTGACTGA

**HMR-1 Constructs:**

NM1605, HMR-1/pRSETA, included a N-terminus His6 tagged domain of HMR-1, amino acids 1099-1223. Primers, #2812: 5’- AGGGATCCGCGATGATGATGGTTGTGTATAC and #2813: 5’- CCGGAATTCCGTTATTGGGCACTTTCGATAC, were used. The fragment was cloned into the BamHI/EcoRI site of the vector pRSETA.

NM1757, HMR-1/pGEX2T, included a N-terminus GST tagged domain of HMR-1 from NM1605. NM1605 was digested with BamHI/EcoRI and the fragment was cloned into the BamHI/EcoRI site of the vector pGEX-2T.

NM1605 insert sequence:

ATGCGGGGTTCTCATCATCATCATCATCATGGTATGGCTAGCATGACTGGTGGACAGCAAATGGGTCGGGATCTGTACGACGATGACGATAAGGATCGATGGGGATCCGCGATGATGATGGTTGTGTATACGAGACGATCGCCTGGTGCATTTGAAAATGTTCGGCCGGAGGAGATGAATCGTGATAATTTGCGGCAGTATGGTGTGGAAGGAGGTGGCGAGGCGGATAACGATCAGTACTCAATGGCCGGCCTACGTAAACCAGTAATGCCACTCGACACAGGAATGGGACCAGCAATCGGAGGACACCCACCACACTACCCACCACGTGGAATGGCGCCACCAAAAGATGATCATGAGCTGAACTCGAAGATCAAGGATCTTGAGACTGATCAGAATGCGGCACCGTACGATGAACTTCGGATCTACGACGATGAGCGGGACAATATTTCTGTCGTCACGTTGGAGAGTATCGAAAGTGCCCAATAAcggaattc

NM1757 insert sequence:

ATGTTCGAAGATCGTTTATGTCATAAAACATATTTAAATGGTGATCATGTAACCCATCCTGACTTCATGTTGTATGACGCTCTTGATGTTGTTTTATACATGGACCCAATGTGCCTGGATGCGTTCCCAAAATTAGTTTGTTTTAAAAAACGTATTGAAGCTATCCCACAAATTGATAAGTACTTGAAATCCAGCAAGTATATAGCATGGCCTTTGCAGGGCTGGCAAGCCACGTTTGGTGGTGGCGACCATCCTCCAAAATCGGATCTGGTTCCGCGTGGATCCGCGATGATGATGGTTGTGTATACGAGACGATCGCCTGGTGCATTTGAAAATGTTCGGCCGGAGGAGATGAATCGTGATAATTTGCGGCAGTATGGTGTGGAAGGAGGTGGCGAGGCGGATAACGATCAGTACTCAATGGCCGGCCTACGTAAACCAGTAATGCCACTCGACACAGGAATGGGACCAGCAATCGGAGGACACCCACCACACTACCCACCACGTGGAATGGCGCCACCAAAAGATGATCATGAGCTGAACTCGAAGATCAAGGATCTTGAGACTGATCAGAATGCGGCACCGTACGATGAACTTCGGATCTACGACGATGAGCGGGACAATATTTCTGTCGTCACGTTGGAGAGTATCGAAAGTGCCCAATAAcggaattc

**ERM-1 Constructs:**

NM1902, ERM-1/SUMOpST, included a N-terminus His6-SUMO tagged ERM-1, amino acids 209-563. Primers, #3317: 5’-AGGGGATCCGCGATGATCCGCAACAAAAAGGGAAC and #3318: 5’-ATAGTTTAGCGGCCGCTTACATATTTTCGTATTGATC, were used. The fragment was cloned into the BamHI/NotI site of the vector pSUMOpST.

NM1995, ERM-1/pRSETA, included a N-terminus His6 tagged ERM-1 from NM1902. NM1902 was digested with BamHI/HindIII and cloned into the BamHI/HindIII site of the vector pRSETA .

NM1902 insert sequence:

ATGGGCAGCAGCCATCATCATCATCATCACGGCAGCGGCCTGGTGCCGCGCGGCAGCGCTAGCATGTCGGACTCAGAAGTCAATCAAGAAGCTAAGCCAGAGGTCAAGCCAGAAGTCAAGCCTGAGACTCACATCAATTTAAAGGTGTCCGATGGATCTTCAGAGATCTTCTTCAAGATCAAAAAGACCACTCCTTTAAGAAGGCTGATGGAAGCGTTCGCTAAAAGACAGGGTAAGGAAATGGACTCCTTAAGATTCTTGTACGACGGTATTAGAATTCAAGCTGATCAGACCCCTGAAGATTTGGACATGGAGGATAACGATATTATTGAGGCTCACAGAGAACAGATTGGTGGTAGCGACCAAGTGGGCGGTCCGGGATCCGCGATGATCCGCAACAAAAAGGGAACTGATCTCTATCTTGGTGTCGATGCTCTTGGATTGAATATTTACGATAAAGCTGATCGTCTTTCGCCGAAAGTCGGATTCCCATGGTCGGAGATTCGTAATATATCATTCAACGACAAGAAATTTGTCATCAAACCAATTGATAAGAAGGCTCATGACTTTGTCTTCTACGCTCCACGACTCCGTATCAACAAACGTATTCTTGCTTTGTGTATGGGAAATCACGAGCTTTACATGCGTAGAAGAAAGCCAGATACCATTGAAGTTCAACAAATGAAGCAACAAGCCAGAGAGGATCGTGCTCTTAAGATTGCCGAGCAGGAGAAGCTCACCAGAGAGATGTCTGCTCGCGAAGAAGCCGAACAGCGTCAACGTGACGCCGAAAAGCGTATGGCTCAAATGCAGGAAGATATGGAGAGAGCTCGTCTTGAATTGGCTGAAGCACACAATACAATTCACTCATTGGAGGCTCAACTCAAGCAATTGCAATTGGCTAAACAAGCATTGGAACAGAAGGAATACGAACTTCGTGAGCTCACTGCTCAACTTCAATCCGAAAAAGCAATGAGTGATGGTGAGAGACGTCATTTGAGAGACCAAGTTGATGCTCGTGAACGTGAAGTTTTTTCGATGAGAGAAGAAGTCGAGAGACAGACTACTGTTACAAGACAACTTCAGACACAGATTCACTCGCAACAACACACTCAACACTACTCAAACAGTCATCACGTTTCCAATGGACACGCTCACGATGAGACTGCCACTGATGATGAAGATAATGGAGCAACTGAACTCACAAATGACGCTGATCAGAATGTGCCACAACACGAGTTGGAGCGTGTCACAGCAGCCGAGAAGAACATTCAGATCAAGAATAAGCTGGATATGTTGACTCGCGAGCTTGACAGTGTTAAAGATCAGAACGCTGTCACTGACTACGACGTTCTGCATATGGAGAACAAAAAGGCCGGACGCGACAAGTACAAGACTCTCCGTCAAATCCGTGGAGGAAACACAAAACGAAGAATCGATCAATACGAAAATATGTAAgcggccgc

NM1995 insert sequence:

ATGCGGGGTTCTCATCATCATCATCATCATGGTATGGCTAGCATGACTGGTGGACAGCAAATGGGTCGGGATCTGTACGACGATGACGATAAGGATCGATGGGGATCCGCGATGATCCGCAACAAAAAGGGAACTGATCTCTATCTTGGTGTCGATGCTCTTGGATTGAATATTTACGATAAAGCTGATCGTCTTTCGCCGAAAGTCGGATTCCCATGGTCGGAGATTCGTAATATATCATTCAACGACAAGAAATTTGTCATCAAACCAATTGATAAGAAGGCTCATGACTTTGTCTTCTACGCTCCACGACTCCGTATCAACAAACGTATTCTTGCTTTGTGTATGGGAAATCACGAGCTTTACATGCGTAGAAGAAAGCCAGATACCATTGAAGTTCAACAAATGAAGCAACAAGCCAGAGAGGATCGTGCTCTTAAGATTGCCGAGCAGGAGAAGCTCACCAGAGAGATGTCTGCTCGCGAAGAAGCCGAACAGCGTCAACGTGACGCCGAAAAGCGTATGGCTCAAATGCAGGAAGATATGGAGAGAGCTCGTCTTGAATTGGCTGAAGCACACAATACAATTCACTCATTGGAGGCTCAACTCAAGCAATTGCAATTGGCTAAACAAGCATTGGAACAGAAGGAATACGAACTTCGTGAGCTCACTGCTCAACTTCAATCCGAAAAAGCAATGAGTGATGGTGAGAGACGTCATTTGAGAGACCAAGTTGATGCTCGTGAACGTGAAGTTTTTTCGATGAGAGAAGAAGTCGAGAGACAGACTACTGTTACAAGACAACTTCAGACACAGATTCACTCGCAACAACACACTCAACACTACTCAAACAGTCATCACGTTTCCAATGGACACGCTCACGATGAGACTGCCACTGATGATGAAGATAATGGAGCAACTGAACTCACAAATGACGCTGATCAGAATGTGCCACAACACGAGTTGGAGCGTGTCACAGCAGCCGAGAAGAACATTCAGATCAAGAATAAGCTGGATATGTTGACTCGCGAGCTTGACAGTGTTAAAGATCAGAACGCTGTCACTGACTACGACGTTCTGCATATGGAGAACAAAAAGGCCGGACGCGACAAGTACAAGACTCTCCGTCAAATCCGTGGAGGAAACACAAAACGAAGAATCGATCAATACGAAAATATGTAAgcggccgcatagtacctaagtactagacaagctt

**LET-413 Constructs:**

NM1617, LET-413/pRSETA, included a N-terminus His6 tagged domain of LET-413, amino acids 460-606. Primers, #2810: 5’- AGGGATCCGCGATGAACAAAGAAGCTGAAATTC and #2811: 5’- CCGGAATTCCGTCATGAATCTCCGTTACTGTTC, were used. The fragment was cloned into the BamHI/EcoRI site of the vector pRSETA.

NM1767, LET-413/pGEX2T, included a N-terminus GST tagged domain of LET-413 from NM1617. NM1617 was digested with BamHI/EcoRI and the fragment was cloned into the BamHI/EcoRI site of the vector pGEX-2T.

NM1617 insert sequence:

ATGCGGGGTTCTCATCATCATCATCATCATGGTATGGCTAGCATGACTGGTGGACAGCAAATGGGTCGGGATCTGTACGACGATGACGATAAGGATCGATGGGGATCCGCGATGAACAAAGAAGCTGAAATTCATCTCGGAAACTTTGAACGGCATAATACACCACATCCAAAGACACCAAAGCATAAGAAAGGATCAATTGATGGTCATATGCTTCCTCATGAAATTGATCAACCACGGCAATTATCTTTGGTTTCAAATCACAGAACATCAACTTCATCATTCGGAGAATCCAGCAATTCAATTAATAGAGATTTAGCTGATATAAGGTTCATTGACGCTCCTGCTTCAGCTCAAAACGGTGTCCGTGAAGCAACATTGTCACCAGAACGAGAAGAACGAATGGCAACCTCCTTGTCAAGTTTGTCAAATCTTGCCGCAGGCACTCAAAACATGCACACAATCAGAATCCAAAAAGACGACACTGGAAAATTGGGACTTTCCTTCGCTGGCGGAACCTCGAACGATCCGGCGCCGAACAGTAACGGAGATTCATGAcggaattc

NM1767 insert sequence:

ATGTTCGAAGATCGTTTATGTCATAAAACATATTTAAATGGTGATCATGTAACCCATCCTGACTTCATGTTGTATGACGCTCTTGATGTTGTTTTATACATGGACCCAATGTGCCTGGATGCGTTCCCAAAATTAGTTTGTTTTAAAAAACGTATTGAAGCTATCCCACAAATTGATAAGTACTTGAAATCCAGCAAGTATATAGCATGGCCTTTGCAGGGCTGGCAAGCCACGTTTGGTGGTGGCGACCATCCTCCAAAATCGGATCTGGTTCCGCGTGGATCCGCGATGAACAAAGAAGCTGAAATTCATCTCGGAAACTTTGAACGGCATAATACACCACATCCAAAGACACCAAAGCATAAGAAAGGATCAATTGATGGTCATATGCTTCCTCATGAAATTGATCAACCACGGCAATTATCTTTGGTTTCAAATCACAGAACATCAACTTCATCATTCGGAGAATCCAGCAATTCAATTAATAGAGATTTAGCTGATATAAGGTTCATTGACGCTCCTGCTTCAGCTCAAAACGGTGTCCGTGAAGCAACATTGTCACCAGAACGAGAAGAACGAATGGCAACCTCCTTGTCAAGTTTGTCAAATCTTGCCGCAGGCACTCAAAACATGCACACAATCAGAATCCAAAAAGACGACACTGGAAAATTGGGACTTTCCTTCGCTGGCGGAACCTCGAACGATCCGGCGCCGAACAGTAACGGAGATTCATGAcggaattc

**SAX-7 Constructs:**

NM1919, SAX-7/SUMOpST, included a N-terminus His6-SUMO tagged SAX-7, amino acids: 1051-1144. Primers, #3319: 5’-AGGGGATCCGCGATGGTTGTCTGCCGTCAACGTGGAC and NMOLIGO #3320: 5’-ATAGTTTAGCGGCCGCCTAGACAAACGTCGACGTTGA, were used. The fragment was cloned into the BamHI/NotI site of the vector pSUMOpST.

NM1998, SAX-7/pRSETA, included a N-terminus His6 tagged SAX-7 from NM1919. NM1919 was digested with BamHI/HindIII and cloned into the BamHI/HindIII site of the vector pRSETA.

NM1893, SAX-7/pGEX4T-1, included a N-terminus GST tagged SAX-7, amino acids 1051-1144. Primers, #3319: 5’-AGGGGATCCGCGATGGTTGTCTGCCGTCAACGTGGAC and NMOLIGO #3320: 5’-ATAGTTTAGCGGCCGCCTAGACAAACGTCGACGTTGA, were used. The fragment was cloned into the BamHI/NotI site of the vector pGEX4T-1.

NM1919 insert sequence:

ATGGGCAGCAGCCATCATCATCATCATCACGGCAGCGGCCTGGTGCCGCGCGGCAGCGCTAGCATGTCGGACTCAGAAGTCAATCAAGAAGCTAAGCCAGAGGTCAAGCCAGAAGTCAAGCCTGAGACTCACATCAATTTAAAGGTGTCCGATGGATCTTCAGAGATCTTCTTCAAGATCAAAAAGACCACTCCTTTAAGAAGGCTGATGGAAGCGTTCGCTAAAAGACAGGGTAAGGAAATGGACTCCTTAAGATTCTTGTACGACGGTATTAGAATTCAAGCTGATCAGACCCCTGAAGATTTGGACATGGAGGATAACGATATTATTGAGGCTCACAGAGAACAGATTGGTGGTAGCGACCAAGTGGGCGGTCCGGGATCCGCGATGGTTGTCTGCCGTCAACGTGGACAAAACTATCCAGTATCACAGCGTGAGCGTGAGCAAGGAAGAGAACCGATTCTTGGAAAACCAGACTACAAAACCGATGATGATGAAAAGCGATCATTGACGGGCTCCAAAGCCGAATCAGAAACTGACAGTATGGCACAATACGGAGACACAGATCCAGGAGTCTTCACTGAAGATGGATCATTTATTGGTCAATACGTTCCACAAAAGAGCTTGATGCCGGCCGAACGGCCCGAGAAAGGATCAACGTCGACGTTTGTCTAGgcggccgc

NM1998 insert sequence:

ATGCGGGGTTCTCATCATCATCATCATCATGGTATGGCTAGCATGACTGGTGGACAGCAAATGGGTCGGGATCTGTACGACGATGACGATAAGGATCGATGGGGATCCGCGATGGTTGTCTGCCGTCAACGTGGACAAAACTATCCAGTATCACAGCGTGAGCGTGAGCAAGGAAGAGAACCGATTCTTGGAAAACCAGACTACAAAACCGATGATGATGAAAAGCGATCATTGACGGGCTCCAAAGCCGAATCAGAAACTGACAGTATGGCACAATACGGAGACACAGATCCAGGAGTCTTCACTGAAGATGGATCATTTATTGGTCAATACGTTCCACAAAAGAGCTTGATGCCGGCCGAACGGCCCGAGAAAGGATCAACGTCGACGTTTGTCTAGgcggccgc

NM1893 insert sequence:

ATGTTCGAAGATCGTTTATGTCATAAAACATATTTAAATGGTGATCATGTAACCCATCCTGACTTCATGTTGTATGACGCTCTTGATGTTGTTTTATACATGGACCCAATGTGCCTGGATGCGTTCCCAAAATTAGTTTGTTTTAAAAAACGTATTGAAGCTATCCCACAAATTGATAAGTACTTGAAATCCAGCAAGTATATAGCATGGCCTTTGCAGGGCTGGCAAGCCACGTTTGGTGGTGGCGACCATCCTCCAAAATCGGATCTGGTTCCGCGTGGATCCGCGATGGTTGTCTGCCGTCAACGTGGACAAAACTATCCAGTATCACAGCGTGAGCGTGAGCAAGGAAGAGAACCGATTCTTGGAAAACCAGACTACAAAACCGATGATGATGAAAAGCGATCATTGACGGGCTCCAAAGCCGAATCAGAAACTGACAGTATGGCACAATACGGAGACACAGATCCAGGAGTCTTCACTGAAGATGGATCATTTATTGGTCAATACGTTCCACAAAAGAGCTTGATGCCGGCCGAACGGCCCGAGAAAGGATCAACGTCGACGTTTGTCTAGgcggccgc

**HCP-4 Constructs:**

NM1554, HCP-4/pGEX6P-1, included a N-terminus GST tagged domain of HCP-4, amino acids 9-210 (gift from A. Audhya).

NM1551, HCP-4/pRSETA, included a N-terminus His6 tagged domain of HCP-4 from NM1554. NM1554 was digested with BamHI/EcoRI and the fragment was cloned into the BamHI/EcoRI site of the vector,pRSETA.

NM1551 insert sequence:

ATGCGGGGTTCTCATCATCATCATCATCATGGTATGGCTAGCATGACTGGTGGACAGCAAATGGGTCGGGATCTGTACGACGATGACGATAAGGATCGATGGGGATCCACGATTGTTCCTGGTCGAAAAATGATCACTGAAATCGTTGCTCTTCGTGAGGCTGGCTTGGACGATACAAGACCGTCGTACTTGGAGGAACCAACAGTTATCGTCGATGAATCGATGATGAATGATAGTGCAAATTTGGAGGAGCGAGAATGGAGAAAACAGGGACTATCTGAGAAGCAGATGTTCGCTATTTTGGAGAAAAGGAAACAAACACAGCTTAACAATGCCAGAAAACGTCTCGAAAAGGAAATGTACGGAGCGAAAACCTTCAAAGAGTTGATTGCTTGTCGTGAATACTCCGAATGTGAGAGCGACAGCGAGAACACTGTGAATCAAAATGTCCGAAGCGCTTCGGTTTTTGCCATTCCGGCGCTTCCGAAACACATCAGTGAAAAGTCGATGATGGGCTCGCCAATTGCTAATTCCAGAGGGAGTGGAAAAGCTGGATTAAGTTGTTCAACTCCCAAGAGCTCGAGTGATACGTCGATGAGGTCCTTGAGATCGCTTGATATTTCACATGTCGTCAATACCGATCGACTAGATGCTGAAAGAGTGACTGTTCACACCAAGTCTGTCGTTATCCCAACCATTCTCGAGGAGAGATGAgaattc

**LMN-1 Constructs:**

NM1475, pJKL410, included a N-terminus His6 tagged full length LMN-1, amino acids 1-566 (gift from JK. Liu).

NM1474, pJKL409, included a N-terminus GST tagged full length LMN-1, amino acids 1-566 (gift from JK. Liu).

**DAO-5 Constructs:**

NM1972, DAO-5sm/pGEX4T-1, included a N-terminus GST tagged DAO-5, amino acids 751-971. Primers, #3383: 5’-AGGGGATCCGCGATGGAGAAGCCAGCTGCCAAG and #2971: 5’-CCGGAATTCCGTCActatcaagttctctgact, were used. The fragment was cloned into the BamHI/EcoRI site of the vector pGEX4T-1.

NM2029, DAO-5s/pVM, included a N-terminus His6 tagged DAO-5 from NM1972. NM1972 was digested with BamHI/NotI and cloned into the BamHI/NotI site of the vector pVM.

NM1972 insert sequence:

ATGTTCGAAGATCGTTTATGTCATAAAACATATTTAAATGGTGATCATGTAACCCATCCTGACTTCATGTTGTATGACGCTCTTGATGTTGTTTTATACATGGACCCAATGTGCCTGGATGCGTTCCCAAAATTAGTTTGTTTTAAAAAACGTATTGAAGCTATCCCACAAATTGATAAGTACTTGAAATCCAGCAAGTATATAGCATGGCCTTTGCAGGGCTGGCAAGCCACGTTTGGTGGTGGCGACCATCCTCCAAAATCGGATCTGGTTCCGCGTGGATCCGCGATGGAGAAGCCAGCTGCCAAGCCAACACCAAAAGCTACTCCTAAGCAATCTGCCAAAAAAGCTGATTCATCCGATGATTCTTCTGATGACGAGGCACCGGCTAAGAAGACACCAGCCAAGTCAACACCTGCAAAAACCGCTGTGAAAAAAGAAGCCTCTTCATCGTCAGACGATTCTTCGGATGATGAGAAAACGAAAAAGAAGTCTGCCACCACACCAGCAAAATCAACTCCAAAGACTGCACTAAAGAAAGCTGAATCTTCTGATTCCTCTGATGACGACGAAGATCTTCCAAAACCATCTAAGGCTGTCACTCCAAGACCACAACGTGCTGACTCTGAAGAATCCGCCGAGACCGAGGAGAGCAGTTCAAGAACACCGGCTTTGAAGGCAAAACCATTGGCTACCTCGACTGAAAAGGCGGTCTATGAAAACCGAAAAAGAAAGTCTTCACCATTCCGACGCGTGCAAATGACAAAGGATAGCGTTTCTGAAAAATTTAGAAACAACCAGCATGATTCGCACTTCGATCAATGGGGACAGCGTGCCAATGAAAGCCTAGGAAAAGTTGTTGGAAAAGCATTCCGTCATGAGAAAACTAAGAAGAAGAAGGGAAGCTACGGAGGTGGTCCAATCAACCAATCTATTAATTCTATCAAGTTCTCTGACTCTGACGATTAGtgacggaattc

NM2029 insert sequence:

ATGCGGGGTTCTCATCATCATCATCATCATGGTATGGCTAGCATGACTGGTGGACAGCAAATGGGTCGGGATCTGTACGACGATGACGATAAGGATCGATGGGGATCCGCGATGGAGAAGCCAGCTGCCAAGCCAACACCAAAAGCTACTCCTAAGCAATCTGCCAAAAAAGCTGATTCATCCGATGATTCTTCTGATGACGAGGCACCGGCTAAGAAGACACCAGCCAAGTCAACACCTGCAAAAACCGCTGTGAAAAAAGAAGCCTCTTCATCGTCAGACGATTCTTCGGATGATGAGAAAACGAAAAAGAAGTCTGCCACCACACCAGCAAAATCAACTCCAAAGACTGCACTAAAGAAAGCTGAATCTTCTGATTCCTCTGATGACGACGAAGATCTTCCAAAACCATCTAAGGCTGTCACTCCAAGACCACAACGTGCTGACTCTGAAGAATCCGCCGAGACCGAGGAGAGCAGTTCAAGAACACCGGCTTTGAAGGCAAAACCATTGGCTACCTCGACTGAAAAGGCGGTCTATGAAAACCGAAAAAGAAAGTCTTCACCATTCCGACGCGTGCAAATGACAAAGGATAGCGTTTCTGAAAAATTTAGAAACAACCAGCATGATTCGCACTTCGATCAATGGGGACAGCGTGCCAATGAAAGCCTAGGAAAAGTTGTTGGAAAAGCATTCCGTCATGAGAAAACTAAGAAGAAGAAGGGAAGCTACGGAGGTGGTCCAATCAACCAATCTATTAATTCTATCAAGTTCTCTGACTCTGACGATTAGtgacggaattcccgggtcgactcgagcggccgc

**ORC-2 Constructs:**

NM1650, ORC-2/pRSETA, included a N-terminus His6 tagged full length ORC-2, amino acids 1-430. Primers, #2816: 5’- GGCCGCTCGAGCGGATGCCACGGCCAAAAAT and #2817: 5’- ATGGTACCCCGGAATTCCGCTACTCGTCTTTC, were used. The fragment was cloned into the XhoI/KpnI site of the vector pRSETA . Sequencing showed two changes in the sequence.

NM1831, ORC-2/pGEX4T-1, included a N-terminus GST tagged full length ORC-2. Primers, #3161: 5’- TCCCCCGGGGATGCCACGGCCAAAAATTTTGAAACGAG and #3162: 5’-GACTGAGGCGGCCGCCTCTCTACTCGTCTTTCTTTTCG, were used. The fragment was cloned into the SmaI/NotI site of the vector pGEX4T-1.

NM1650 insert sequence:

ATGCGGGGTTCTCATCATCATCATCATCATGGTATGGCTAGCATGACTGGTGGACAGCAAATGGGTCGGGATCTGTACGACGATGACGATAAGGATCGATGGGGATCCGAGCTCGAGCGGATGCCACGGCCAAAAATTTTGAAACGAGCAACTGTCCAGCCCAGTGCCGCCGTTCCTGTGAAAAAATCGACTCCAGAAAAAGAAGGATCCAGACAGAAAAAGACGAATGGAAAAGAGAATGCTTCTAGAAATTTGCAATCAAATTTAGAAGAAGATTTGGAACAACTGGGCTTCGAGGATGAAACTGTATCAATGGCTCAATCAGCAATCGAAAATTACTTTATGCAAGGAAAATCGGCGTCAGAACGAATGAATAATGCGAAATCCCGTCGTGGAAGACGTGCTGGAAATGGAAATACTGAAGAAATTGAGGAAGACGATGAGATCAGTAATGCTATCACTGATTTCACAAAATGTGATCTCCCTGGACTTCGAAATTATATTACCAAAAAAGATAACACGGAATTCGAAAAACGATTGGAGCATCTCGCGGATAATGATTTCGGAAAATGGAAGCTTTACCTAGCGGCTGGATTTAATATTCTTTTGCACGGTGTCGGTTCGAAGCGTGATGTTCTCACAGAATTTGAGAATGAGCTATCCGATTATACATATATGAGAGTGGATGCACGGAAAGATGGGCTCAACGTAAAAGTTCTTCTTGGAGCTATCAATGAGAATATGAAGCTGAATTGTAATGTGAAGAGAGGCCAATCTACGATTAGTTGGGCTCGATCTATTCGCAGAAAAATGAATAGCCAACAGTTGATTCTTATCATTGATAATATTG

AAGCTCCTGATTGGAGAAGTGATCAAGAAGCATTTTGCGAACTTCTTGAGAATCGGGATTCGGTGAAATTGATTGCTACAGTTGATCACATTTACTCGACGTTCATCTGGAATTCGCGTCAACTATCATCACTCTCATTCGTTCACATCACAATCAACACCTTCGAAATTCCACTTCAAGAATTAATGACTGGAGATTCTCGTCTTCTTGGTCTTGATGCTCGTTCGAATCAATCCTCTCATACAATGTCATCGCTTGATGTGTTCTGGAAATCTCTTGCCGTCAATTCACAAAAATTATTCCGTCTCTTTTTCCAAATGTACTTTGACACCAAGAAGCCTGTCAAATTCTGGGATTTGTTCAATGCGGCAAAAGATGATTTCATTGCTTCAACTGACGCTGCTCTTCGAACCCAACTTGTCGAATTCAAGGATCATCGGGTTTTGAAGTGGACCCGTGGTGATGACGGAAACGATCAGCTGTCGGGCATTGTCGAATTACGATTAGTGACCGAATTTCTCGAATCGAAGAACATGCCGTTAGACGAAAAGAAAGACGAGTAGcggaattccggggtacc

NM1831 insert sequence:

ATGTTCGAAGATCGTTTATGTCATAAAACATATTTAAATGGTGATCATGTAACCCATCCTGACTTCATGTTGTATGACGCTCTTGATGTTGTTTTATACATGGACCCAATGTGCCTGGATGCGTTCCCAAAATTAGTTTGTTTTAAAAAACGTATTGAAGCTATCCCACAAATTGATAAGTACTTGAAATCCAGCAAGTATATAGCATGGCCTTTGCAGGGCTGGCAAGCCACGTTTGGTGGTGGCGACCATCCTCCAAAATCGGATCTGGTTCCGCGTGGATCCCCGGAATTCCCGGGGATGCCACGGCCAAAAATTTTGAAACGAGCAACTGTCCAGCCCAGTGCCGCCGTTCCTGTGAAAAAATCGACTCCAGAAAAAGAAGGATCCAGACAGAAAAAGACGAATGGAAAAGAGAATGCTTCTAGAAATTTGCAATCAAATTTAGAAGAAGATTTGGAACAACTGGGCTTCGAGGATGAAACTGTATCAATGGCTCAATCAGCAATCGAAAATTACTTTATGCAAGGAAAATCGGCGTCAGAACGAATGAATAATGCGAAATCCCGTCGTGGAAGACGTGCTGGAAATGGAAATACTGAAGAAATTGAGGAAGACGATGAGATCAGTAATGCTATCACTGATTTCACAAAATGTGATCTCCCTGGACTTCGAAATTATATTACCAAAAAAGATAACACGGAATTCGAAAAACGATTGGAGCATCTCGCGGATAATGATTTCGGAAAATGGAAGCTTTACCTAGCAGCTGGATTTAATATTCTTTTGCACGGTGTCGGTTCGAAGCGTGATGTTCTCACAGAATTTGAGAATGAGCTATCCGATTATACATATATGAGAGTGGATGCACGGAAAGATGGGCTCAATGTAAAAGTTCTTCTTGGAGCTATCAATGAGAATATGAAGCTGAATTGTAATGTGAAGAGAGGCCAATCTACGATTAGTTGGGCTCGATCTATTCGCAGAAAAATGAATAGCCAACAGTTGATTCTTATCATTGATAATATTGAAGCTCCTGATTGGAGAAGTGATCAAGAAGCATTTTGCGAACTTCTTGAGAATCGGGATTCGGTGAAATTGATTGCTACAGTTGATCACATTTACTCGACGTTCATCTGGAATTCGCGTCAACTATCATCACTCTCATTCGTTCACATCACAATCAACACCTTCGAAATTCCACTTCAAGAATTAATGACTGGAGATTCTCGTCTTCTTGGTCTTGATGCTCGTTCGAATCAATCCTCTCATACAATGTCATCGCTTGATGTGTTCTGGAAATCTCTTGCCGTCAATTCACAAAAATTATTCCGTCTCTTTTTCCAAATGTACTTTGACACCAAGAAGCCTGTCAAATTCTGGGATTTGTTCAATGCGGCAAAAGATGATTTCATTGCTTCAACTGACGCTGCTCTTCGAACCCAACTTGTCGAATTCAAGGATCATCGGGTTTTGAAGTGGACCCGTGGTGATGACGGAAACGATCAGCTGTCGGGCATTGTCGAATTACGATTAGTGACCGAATTTCTCGAATCGAAGAACATGCCGTTAGACGAAAAGAAAGACGAGTAGagaggcggccgc

**PAS-7 Constructs:**

NM1603, PAS-7/pRSETA, included a N-terminus His6 tagged full length PAS-7, amino acids 1-250. Primers, #2694: 5’- CGGGGATCCGCGATGAGTTCAATCGGTACCG and #2695: 5’- CCGGAATTCCGTCAATCGTCCATGTCGTCCT, were used. The fragment was cloned into the BamHI/EcoRI site of the vector pRSETA .

NM1648, PAS-7/pGEX4T-1, included a N-terminus His6 tagged full length PAS-7. Primers, #2694: 5’—CGGGGATCCGCGATGAGTTCAATCGGTACCG and #2696: 5’- GGCCGCTCGAGTTATCAATCGTCCATGTCGTC, were used. The fragment was cloned into the BamHI/XhoI site of the vector pRSETA .

NM1603 insert sequence:

ATGCGGGGTTCTCATCATCATCATCATCATGGTATGGCTAGCATGACTGGTGGACAGCAAATGGGTCGGGATCTGTACGACGATGACGATAAGGATCGATGGGGATCCGCGATGAGTTCAATCGGTACCGGCTACGATCTCGCCGCCTCTACTTTTTCTCCTGATGGCAGAATTTTCCAAGTCGAGTACGCTCAAAAGGCTGTCGACAATGCAGGAACAATGATTGCAATTCGTGGCAAGAACGGAGTTGTGGTCGTTGCTGACAAGCTGATCTCTTCCAAACTTTACACTGATAACGCTAATCCAAGAATGTTTAACGTGAACGACAATGTTGGTGTCGCAGTTGCTGGAAACTATCCAGATGGTTTCGCTCTGAAGAACTATGCTTACGGAGAAGCAATGAAGTGGCTCAAAGACTACCGTGAGCCAATGCCGATTCAGAATATTGCTAACTCAGTTGCTGAGTACATTCACATTCACACTCTCGGCATTAGTCGTCCGTTTGGAGCAGGAGCCTTCTTCATGTCATGGAACAAACAAACTGGAGGACGTCTTTTCCTTGTGGAGCCATCAGGTCTAAACTATGAATACAAGGCATGGGCTGTCGGAAAACATCGCCAAGCCGCAAAAGCTGAGATTGAGAAGCTGAAGATCGAGGAGCTGGATGTGAATCAACTCGTGAAGGAAGCTGCTCGAATCATTATGGTGGTGCGTGACGAGAACAAGGATAAAAACGTTCAAATCGAAATGGGATGGGTCGGTGAGCAGACGAACGGAAAGTACGAAGAAGTTCCAAGCGAGGTTGTCACTGCTGCCGAGGAATGGGCTATTGCCAAGCTTGACGAGGACGACATGGACGATTGAcggaattc

NM1648 insert sequence:

ATGTTCGAAGATCGTTTATGTCATAAAACATATTTAAATGGTGATCATGTAACCCATCCTGACTTCATGTTGTATGACGCTCTTGATGTTGTTTTATACATGGACCCAATGTGCCTGGATGCGTTCCCAAAATTAGTTTGTTTTAAAAAACGTATTGAAGCTATCCCACAAATTGATAAGTACTTGAAATCCAGCAAGTATATAGCATGGCCTTTGCAGGGCTGGCAAGCCACGTTTGGTGGTGGCGACCATCCTCCAAAATCGGATCTGGTTCCGCGTGGATCCGCGATGAGTTCAATCGGTACCGGCTACGATCTCGCCGCCTCTACTTTTTCTCCTGATGGCAGAATTTTCCAAGTCGAGTACGCTCAAAAGGCTGTCGACAATGCAGGAACAATGATTGCAATTCGTGGCAAGAACGGAGTTGTGGTCGTTGCTGACAAGCTGATCTCTTCCAAACTTTACACTGATAACGCTAATCCAAGAATGTTTAACGTGAACGACAATGTTGGTGTCGCAGTTGCTGGAAACTATCCAGATGGTTTCGCTCTGAAGAACTATGCTTACGGAGAAGCAATGAAGTGGCTCAAAGACTACCGTGAGCCAATGCCGATTCAGAATATTGCTAACTCAGTTGCTGAGTACATTCACATTCACACTCTCGGCATTAGTCGTCCGTTTGGAGCAGGAGCCTTCTTCATGTCATGGAACAAACAAACTGGAGGACGTCTTTTCCTTGTGGAGCCATCAGGTCTAAACTATGAATACAAGGCATGGGCTGTCGGAAAACACCGCCAAGCCGCAAAAGCTGAGATTGAGAAGCTGAAGATCGAGGAGCTGGATGTGAATCAACTCGTGAAGGAAGCTGCTCGAATCATTATGGTGGTGCGTGACGAGAACAAGGATAAAAACGTTCAAATCGAAATGGGATGGGTCGGTGAGCAGACGAACGGAAAGTACGAAGAAGTTCCAAGCGAGGTTGTCACTGCTGCCGAGGAATGGGCTATTGCCAAGCTTGACGAGGACGACATGGACGATTGAtaactcgag

**TAC-1 Constructs:**

NM2068, TAC-1N/pVM, included a N-terminus His6 tagged full length version of TAC-1, amino acids 1-260. Primers, #3459: 5’- CCGGGATCCATGTCGCTCAACACAACCTTC and #3338: 5’-AAGAGGCGGCCGCCTTATGCATCCGTCGAAATAACG, were used. The fragment was cloned into the BamHI/NotI site of the vector pVM.

NM1979, TAC-1/pGEX-4T, included a N-terminus GST tagged full length version of TAC-1. Primers, #3337: 5’-CCGGGATCCGCGATGTCGCTCAACACAACCTTCACCA and #3338: 5’-AAGAGGCGGCCGCCTTATGCATCCGTCGAAATAACG, were used. The fragment was cloned into the BamHI/NotI site of the vector pGEX4T-1. Sequencing showed two changes in the sequence. A conservative change (GCC to GCT (A to A)) at amino acid #146 (nucleotide #1088)), and a semi-conservative change (AAA to CAA (K to Q)) at amino acid #105 (nucleotide #963)).

NM2068 insert sequence:

ATGCGGGGTTCTCATCATCATCATCATCATGGTATGGCTAGCATGACTGGTGGACAGCAAATGGGTCGGGATCTGTACGACGATGACGATAAGGATCGATGGGGATCCGCGATGTCGCTCAACACAACCTTCACCAAAGAGGACGGAACCGAGGTCGTCATCCCATTCAACGGGTCGCAAAATGGGCATCCGGAGAACGAGGAGCCAGAGGTTGAAGAAGCCGCGGAGCCCAGTTCTTCAGTGGAGACACTGTGCGGTGCCACGAGAGGCGACATCATTGTGATGAAGCACACGACGAAGGCGCTCACAGAGTTGATCGAGCGGCTCTTGCACTCGGATGAATTCGAAGTCCGTCGCTGCTCAAACGGCCAAATCATCTCACAAGGCCGCTGCAACGGAACAACGCCAGGAAACGGCATCGGAGGCGGCGGGGCATCAAGCGAGGAGCTCGAAAAAGCGCTGAAAGATCGAGATGCCGCCCGTGCCGAAGCCGACAAGCTCCACGCCAACTACGCGACACTGTTCGCCTCGTTCAACACGGTTCGCGAAGCCGCCAACGACATCCGCGGCGAGTACGAGGACGCGAGGGACAAGCTGAAGCTCGCCGCCGCCGAAGTTGACGAGTGGCAGGCAAAGTTCCTCGCCGTCAAGGATAATGCGAATTCTGAGCTTGAGAGAGCATCCGTGGAGTACGACGATCTTCTCCGATCCCACGATGAGAACACAAAGGGACTCCGTCTGCGTGTAAAACGCCAGGAAATCGAGCTCTCCAGCAAGAACGACGAGATCAAGGTGCTCACGAACCGCGTGTCGGAGCTCTCACAGATTTGCGATCAACTTCTGAATGATGTCGACGTTTCCGATGGGATGTCCGTTATTTCGACGGATGCATAAggcggccgc

NM1979 insert sequence:

ATGTTCGAAGATCGTTTATGTCATAAAACATATTTAAATGGTGATCATGTAACCCATCCTGACTTCATGTTGTATGACGCTCTTGATGTTGTTTTATACATGGACCCAATGTGCCTGGATGCGTTCCCAAAATTAGTTTGTTTTAAAAAACGTATTGAAGCTATCCCACAAATTGATAAGTACTTGAAATCCAGCAAGTATATAGCATGGCCTTTGCAGGGCTGGCAAGCCACGTTTGGTGGTGGCGACCATCCTCCAAAATCGGATCTGGTTCCGCGTGGATCCGCGATGTCGCTCAACACAACCTTCACCCAAGAGGACGGAACCGAGGTCGTCATCCCATTCAACGGGTCGCAAAATGGGCATCCGGAGAACGAGGAGCCAGAGGTTGAAGAAGCCGCGGAGCCCAGTTCTTCAGTGGAGACACTGTGCGGTGCTACGAGAGGCGACATCATTGTGATGAAGCACACGACGAAGGCGCTCACAGAGTTGATCGAGCGGCTCTTGCACTCGGATGAATTCGAAGTCCGTCGCTGCTCAAACGGCCAAATCATCTCACAAGGCCGCTGCAACGGAACAACGCCAGGAAACGGCATCGGAGGCGGCGGGGCATCAAGCGAGGAGCTCGAAAAAGCGCTGAAAGATCGAGATGCCGCCCGTGCCGAAGCCGACAAGCTCCACGCCAACTACGCGACACTGTTCGCCTCGTTCAACACGGTTCGCGAAGCCGCCAACGACATCCGCGGCGAGTACGAGGACGCGAGGGACAAGCTGAAGCTCGCCGCCGCCGAAGTTGACGAGTGGCAGGCAAAGTTCCTCGCCGTCAAGGATAATGCGAATTCTGAGCTTGAGAGAGCATCCGTGGAGTACGACGATCTTCTCCGATCCCACGATGAGAACACAAAGGGACTCCGTCTGCGTGTAAAACGCCAGGAAATCGAGCTCTCCAGCAAGAACGACGAGATCAAGGTGCTCACGAACCGCGTGTCGGAGCTCTCACAGATTTGCGATCAACTTCTGAATGATGTCGACGTTTCCGATGGGATGTCCGTTATTTCGACGGATGCATAAggcggccgc

**HSP-60 Constructs:**

NM1496, pRSETA-HSP60, included a N-terminus His6 tagged HSP-60, amino acids: 1-547. Primers, #2451: 5’- CGCGGATCCGCGATGCTTCGCCTCGCCAGA and #2452: 5’- CCGGAATTCCGTTAGAATCCCATTCCTCC, were used. The fragment was cloned into the BamHI/EcoRI site of the vector pRSETA.

NM1759, HSP-60/pGEX2T, included a N-terminus GST tagged HSP-60 from NM1496. NM1496 was digested with BamHI/EcoRI, and the fragment was cloned into the BamHI/EcoRI site of the vector pGEX-2T.

NM1496 insert sequence:

ATGCGGGGTTCTCATCATCATCATCATCATGGTATGGCTAGCATGACTGGTGGACAGCAAATGGGTCGGGATCTGTACGACGATGACGATAAGGATCGATGGGGATCCGCGATGCTTCGCCTCGCCAGAAAGGGACTTCAGACCGCCGTCGTCAGATCTTACGCCAAGGACGTCAAGTTCGGAGCCGAAGGACGACAGGCCATGCTCGTCGGAGTCAACCTGCTCGCCGACGCCGTCTCTGTCACTATGGGCCCAAAAGGAAGAAACGTGATCATCGAGCAGTCGTGGGGAAGCCCAAAGATCACAAAGGACGGAGTCACCGTCGCGAAATCCATCGACCTCAAGGACAAATACCAGAACCTGGGAGCCAAGCTCATTCAAGATGTCGCCAACAAGGCTAATGAGGAGGCTGGAGACGGAACCACCTGCGCCACAGTTCTCGCCAGAGCCATCGCCAAAGAGGGATTCGAGAGCATTCGTCAAGGCGGAAACGCTGTCGAGATCCGTCGTGGAGTCATGAACGCCGTCGAAGTCGTCGTTGCAGAGCTCAAGAAGATCTCCAAGAAGGTCACCACCCCCGAAGAGATCGCTCAAGTCGCCACAATTTCCGCCAATGGAGACACCGTCGTCGGAAATCTCATCAGTGACGCCATGAAGAAGGTCGGAACCACTGGAGTCATCACTGTTAAGGACGGAAAAACGCTGAACGATGAGCTCGAGCTTATCGAGGGAATGAAGTTCGACAGAGGATACATCTCGCCGTACTTTATCACCTCTGCGAAAGGAGCTAAAGTCGAGTACGAGAAGGCTCTTGTCTTGTTGAGCGAGAAGAAGATCAGCCAAGTTCAGGATATTGTGCCGGCTCTTGAGCTCGCCAACAAGCTCCGCCGTCCATTGGTTATCATTGCCGAGGATGTTGATGGAGAGGCTCTTACCACTCTTGTTCTTAATAGACTTAAGGTCGGACTTCAAGTCGTCGCAATCAAGGCTCCAGGATTCGGAGATAACCGCAAGAACACGCTCAAGGATATGGGAATTGCGACGGGAGCAACGATCTTTGGAGATGACTCGAATCTCATTAAGATCGAGGATATCACAGCCAACGATCTCGGAGAAGTCGATGAGGTCACCATCACCAAGGACGATACTCTTCTTCTTCGCGGGCGTGGAGATCAAACAGAGATCGAGAAGCGAATCGAGCACATCACCGATGAAATTGAGCAATCGACGAGCGATTACGAGAAGGAGAAGCTTAATGAGCGTCTCGCCAAGTTGAGCAAGGGAGTCGCTGTGCTCAAGATTGGAGGAGGATCTGAAGTTGAAGTTGGAGAGAAGAAGGACCGTGTCACCGATGCTCTGTGTGCCACGCGTGCCGCTGTCGAAGAAGGAATCGTTCCAGGAGGAGGTGTTGCTCTTCTCAGATCTCTGACAGCACTCAAAAACTACAAAGCAGCCAACGAGGATCAACAGATCGGTGTGAATATCGTCAAGAAGGCTCTCACTCAGCCAATCGCCACAATCGTCAAAAATGCCGGTCTTGAGCCATCGTCGATTATTGATGAAGTCACCGGAAACAGCAATACATCATATGGTTATGATGCGCTCAACGGAAAGTTCGTTGACATGTTCGAGGCTGGAATTATCGATCCAACTAAGGTGGTTCGCACAGCTCTCCAAGATGCTTCAGGAGTCGCCTCACTTCTCGCCACAACCGAATGCGTAGTCACCGAAATTCCAAAGGAAGAAGCAGTTGGTGGACCAGCCGGTGGAATGGGTGGAATGGGTGGAATGGGCGGTATGGGAGGAATGGGATTCTAAcggaattc

NM1759 insert sequence:

ATGTTCGAAGATCGTTTATGTCATAAAACATATTTAAATGGTGATCATGTAACCCATCCTGACTTCATGTTGTATGACGCTCTTGATGTTGTTTTATACATGGACCCAATGTGCCTGGATGCGTTCCCAAAATTAGTTTGTTTTAAAAAACGTATTGAAGCTATCCCACAAATTGATAAGTACTTGAAATCCAGCAAGTATATAGCATGGCCTTTGCAGGGCTGGCAAGCCACGTTTGGTGGTGGCGACCATCCTCCAAAATCGGATCTGGTTCCGCGTGGATCCGCGATGCTTCGCCTCGCCAGAAAGGGACTTCAGACCGCCGTCGTCAGATCTTACGCCAAGGACGTCAAGTTCGGAGCCGAAGGACGACAGGCCATGCTCGTCGGAGTCAACCTGCTCGCCGACGCCGTCTCTGTCACTATGGGCCCAAAAGGAAGAAACGTGATCATCGAGCAGTCGTGGGGAAGCCCAAAGATCACAAAGGACGGAGTCACCGTCGCGAAATCCATCGACCTCAAGGACAAATACCAGAACCTGGGAGCCAAGCTCATTCAAGATGTCGCCAACAAGGCTAATGAGGAGGCTGGAGACGGAACCACCTGCGCCACAGTTCTCGCCAGAGCCATCGCCAAAGAGGGATTCGAGAGCATTCGTCAAGGCGGAAACGCTGTCGAGATCCGTCGTGGAGTCATGAACGCCGTCGAAGTCGTCGTTGCAGAGCTCAAGAAGATCTCCAAGAAGGTCACCACCCCCGAAGAGATCGCTCAAGTCGCCACAATTTCCGCCAATGGAGACACCGTCGTCGGAAATCTCATCAGTGACGCCATGAAGAAGGTCGGAACCACTGGAGTCATCACTGTTAAGGACGGAAAAACGCTGAACGATGAGCTCGAGCTTATCGAGGGAATGAAGTTCGACAGAGGATACATCTCGCCGTACTTTATCACCTCTGCGAAAGGAGCTAAAGTCGAGTACGAGAAGGCTCTTGTCTTGTTGAGCGAGAAGAAGATCAGCCAAGTTCAGGATATTGTGCCGGCTCTTGAGCTCGCCAACAAGCTCCGCCGTCCATTGGTTATCATTGCCGAGGATGTTGATGGAGAGGCTCTTACCACTCTTGTTCTTAATAGACTTAAGGTCGGACTTCAAGTCGTCGCAATCAAGGCTCCAGGATTCGGAGATAACCGCAAGAACACGCTCAAGGATATGGGAATTGCGACGGGAGCAACGATCTTTGGAGATGACTCGAATCTCATTAAGATCGAGGATATCACAGCCAACGATCTCGGAGAAGTCGATGAGGTCACCATCACCAAGGACGATACTCTTCTTCTTCGCGGGCGTGGAGATCAAACAGAGATCGAGAAGCGAATCGAGCACATCACCGATGAAATTGAGCAATCGACGAGCGATTACGAGAAGGAGAAGCTTAATGAGCGTCTCGCCAAGTTGAGCAAGGGAGTCGCTGTGCTCAAGATTGGAGGAGGATCTGAAGTTGAAGTTGGAGAGAAGAAGGACCGTGTCACCGATGCTCTGTGTGCCACGCGTGCCGCTGTCGAAGAAGGAATCGTTCCAGGAGGAGGTGTTGCTCTTCTCAGATCTCTGACAGCACTCAAAAACTACAAAGCAGCCAACGAGGATCAACAGATCGGTGTGAATATCGTCAAGAAGGCTCTCACTCAGCCAATCGCCACAATCGTCAAAAATGCCGGTCTTGAGCCATCGTCGATTATTGATGAAGTCACCGGAAACAGCAATACATCATATGGTTATGATGCGCTCAACGGAAAGTTCGTTGACATGTTCGAGGCTGGAATTATCGATCCAACTAAGGTGGTTCGCACAGCTCTCCAAGATGCTTCAGGAGTCGCCTCACTTCTCGCCACAACCGAATGCGTAGTCACCGAAATTCCAAAGGAAGAAGCAGTTGGTGGACCAGCCGGTGGAATGGGTGGAATGGGTGGAATGGGCGGTATGGGAGGAATGGGATTCTAAcggaattc

**CAV-1 Constructs:**

NM1489, CAV-1/pRSETA, included a N-terminus His6 tagged full length CAV-1, amino acids 1-235. Primers, #2549: 5’- CGGGGATCCGCGATGTCCACCGAGCAAGAT and NMOLIGO #2550: 5’- CCGGAATTCCGTTAGACGCATGGAGCAGTAG, were used. The fragment was cloned into the BamHI/EcoRI site of the vector pRSETA.

NM1765, CAV-1/pGEX2T, included a N-terminus GST tagged CAV-1 from NM1504. NM1489 was digested with BamHI/EcoRI, and the fragment was cloned into the BamHI/EcoRI site of the vector pGEX-2T.

NM1489 insert sequence:

ATGCGGGGTTCTCATCATCATCATCATCATGGTATGGCTAGCATGACTGGTGGACAGCAAATGGGTCGGGATCTGTACGACGATGACGATAAGGATCGATGGGGATCCGCGATGTCCACCGAGCAAGATATCAAGACAGAGGAACAAATTCCACTGACATACGCTGCAGTCGCCGCACCAACAGTCCAGACAGAAGGAGAAGCTGTTGTTGCTCCAGAAGAGCCAAAGCCAAAGAAGAACTGGTTCACATTCGGAAAGAAGAAGGCTGCTCCAACTGATGAGACCAATATTGAAGAAGGTGGAGCACCGGGAGATGAGCCAGTGAAGGAGAAGAAAGAGAAGAAGTGCTGGTGGAGTAGATGCCAAAAAGGAGAAGGCGAGCAAAAGGAAGAGAACATCGCTATCGGAGTCGATCTCGTCAATCGTGACGCCAACTCGATGAACAACCATGTCCAACTCAACTTTGAAGACATTTTCGGAGAAGCCGACTCACAGCACTCATGGGATTGCGTGTGGCGTCTGAATCATACCGTATTCACTGCCGTCCGGCTCTTCATCTATCGTCTTGTCTCACTCTTGGCTCTTCCATTCACAATCATCTTTGCCATCTTCTTCGGACTTCTCGCATCGATCAACGTCTTCATCATTGTTCCACTTGGAAAACTTCTCTCGATTCCAGGAACTCTTCTCGCCAAGCTCTGGAACTGGTTGATTCATGCCATCTTTGATCCAATTGCCAGTGCTGTTGGACTAATCTTCTCCAATTTCAACATCAGAAAATACGGAATCAATCAAGAAACTACTGCTCCATGCGTCTAA cggaattc

NM1765 insert sequence:

ATGTTCGAAGATCGTTTATGTCATAAAACATATTTAAATGGTGATCATGTAACCCATCCTGACTTCATGTTGTATGACGCTCTTGATGTTGTTTTATACATGGACCCAATGTGCCTGGATGCGTTCCCAAAATTAGTTTGTTTTAAAAAACGTATTGAAGCTATCCCACAAATTGATAAGTACTTGAAATCCAGCAAGTATATAGCATGGCCTTTGCAGGGCTGGCAAGCCACGTTTGGTGGTGGCGACCATCCTCCAAAATCGGATCTGGTTCCGCGTGGATCCGCGATGTCCACCGAGCAAGATATCAAGACAGAGGAACAAATTCCACTGACATACGCTGCAGTCGCCGCACCAACAGTCCAGACAGAAGGAGAAGCTGTTGTTGCTCCAGAAGAGCCAAAGCCAAAGAAGAACTGGTTCACATTCGGAAAGAAGAAGGCTGCTCCAACTGATGAGACCAATATTGAAGAAGGTGGAGCACCGGGAGATGAGCCAGTGAAGGAGAAGAAAGAGAAGAAGTGCTGGTGGAGTAGATGCCAAAAAGGAGAAGGCGAGCAAAAGGAAGAGAACATCGCTATCGGAGTCGATCTCGTCAATCGTGACGCCAACTCGATGAACAACCATGTCCAACTCAACTTTGAAGACATTTTCGGAGAAGCCGACTCACAGCACTCATGGGATTGCGTGTGGCGTCTGAATCATACCGTATTCACTGCCGTCCGGCTCTTCATCTATCGTCTTGTCTCACTCTTGGCTCTTCCATTCACAATCATCTTTGCCATCTTCTTCGGACTTCTCGCATCGATCAACGTCTTCATCATTGTTCCACTTGGAAAACTTCTCTCGATTCCAGGAACTCTTCTCGCCAAGCTCTGGAACTGGTTGATTCATGCCATCTTTGATCCAATTGCCAGTGCTGTTGGACTAATCTTCTCCAATTTCAACATCAGAAAATACGGAATCAATCAAGAAACTACTGCTCCATGCGTCTAAcggaattc

**CYP-33E1 Constructs:**

NM1545, CYC33E1/pRSETA, included a full length N-terminus His6 tagged domain of CYP-33E1, amino acids 1-494. Primers, #2551: 5’- GGCCGCTCGAGGCGATGATTTTACTTATTCTC and #2552: 5’- CGCGAAGCTTGCTCAATATCTTTCCTTCATTG, were used. The fragment was cloned into the XhoI/HindIII site of the vector pRSETA .

NM1873, CYC33E1/pGEX4T-1, included a full length N-terminus GST tagged domain of CYP-33E1. Primers, #3164: 5’- TCCCCCGGGGATGATTTTACTTATTCTCACTTCGATATT and #3165: 5’- GACTGAGGCGGCCGCCTCTTCAATATCTTTCCTTCATTG, were used. The fragment was cloned into the SmaI/NotI site of the vector pGEX4T-1.

NM1545 insert sequence:

ATGCGGGGTTCTCATCATCATCATCATCATGGTATGGCTAGCATGACTGGTGGACAGCAAATGGGTCGGGATCTGTACGACGATGACGATAAGGATCGATGGGGATCCGAGCTCGAGGCGATGATTTTACTTATTCTCACTTCGATATTAATTATCTACCTCTTTAATCATTTTTACTGGAAGAGAAGAAAATTACCACCAGGTCCAATTCCACTCCCTATTATTGGAAATTTATATCTTATGACGGAAGATGTTAAGCCAGGATACAAGATGTACGAGAAATTGAAAGACAAATATGGTCCAGTGTTCACATTTTGGTTGGCAAATCTTCCAATGGTTACTGTGACTGATTGGAAACTCATCAAGCAGCATTTCATAAAAGATGGAGCCAACTTTGTAGGAAGACCAGAGTTTCCAATTTCAATGGAAATGAGACAAGGTCCATATGGAATTATTGAATCTCATGGTGATAGATGGATTCAGCAAAGAAGATTTGCATTGCATATTCTTCGAGATTTTGGATTGGGAAAGAATTTGATGGAAGAAAAGGTTCTTGGTGAAGTTACTGCAATGATTGACAGTATTCGAAAAAGCATGGAAGATGTTGATATGCAAAATATATTTGATGCATCCGTGGGATCCGTAATCAATAATATGCTTTTTGGATATCGTTATGATGAGACAAACATTGAAGAATTTCTGGAACTCAAAAATCGAATGAATAAGCATTTCAAGTTAGCTGCAGAACCAATGGGAGGACTTATTGGAATGAATCCATGGCTTGGACATCTCCCGTTTTTCAAGGGATATAAAAATGTAATAATGCACAACTGGATGGGTCTCATGGAAATGTTCCGTAAGCAGGCTACAGATAGACTTGCATCAATTGACTATGATTCTGATGAATATTCGGATTACGTAGAAGCATTTTTGAAAGAAAGAAAGAAACATGAAAATGAGCAAGATTTCGGAGGATTTGAAATGGAACAACTTGATAGTGTATGTTTTGATCTATGGGTTGCTGGAATGGAAACAACTTCAAATACTCTCAATTGGGCACTTCTCTATGTCCTTTTGAATCCAGAAGTACGTCAAAAAGTCTATGAAGAATTGGAAAGAGAAATTGGAAGTGATAGGATTATTACAACTACCGACCGACCTAAACTCAACTATATCAATGCAACTGTTAATGAATCTCAACGTCTTGCTAATCTTCTTCCAATGAATCTATCTAGATCTACAAATGCTGATGTTGAAATTGCTGGCTACCGTATTCCAAAAGATACTGTAATCACTCCACAAATCAGTTCAGTAATGTATGATCCAGAGATATTCCCTGAACCATACGAATTCAAACCTGAAAGATTCCTAGAATCGGATGGAAGTTTGAAAAAAGTAGAAGAACTTGTGCCATTTTCAATTGGAAAACGGCAATGTCTTGGAGAAGGACTGGCCAAAATGGAACTATTCTTGTATTTTGCAAATTTGTTCAACAAATTTGATATTAAATTTCACGAATCGAATCCAAATCCAAGTATTAAAAAGGAAGTCGGAGTTACTATGAAGGCAAAGAATTACAGAGTTTCAATGAAGGAAAGATATTGAgcaagctt

NM1873 insert sequence:

ATGTTCGAAGATCGTTTATGTCATAAAACATATTTAAATGGTGATCATGTAACCCATCCTGACTTCATGTTGTATGACGCTCTTGATGTTGTTTTATACATGGACCCAATGTGCCTGGATGCGTTCCCAAAATTAGTTTGTTTTAAAAAACGTATTGAAGCTATCCCACAAATTGATAAGTACTTGAAATCCAGCAAGTATATAGCATGGCCTTTGCAGGGCTGGCAAGCCACGTTTGGTGGTGGCGACCATCCTCCAAAATCGGATCTGGTTCCGCGTGGATCCCCGGAATTCCCGGGGATGATTTTACTTATTCTCACTTCGATATTAATTATCTACCTCTTTAATCATTTTTACTGGAAGAGAAGAAAATTACCACCAGGTCCAATTCCACTCCCTATTATTGGAAATTTATATCTTATGACGGAAGATGTTAAGCCAGGATACAAGATGTACGAGAAATTGAAAGACAAATATGGTCCAGTGTTCACATTTTGGTTGGCAAATCTTCCAATGGTTACTGTGACTGATTGGAAACTCATCAAGCAGCATTTCATAAAAGATGGAGCCAACTTTGTAGGAAGACCAGAGTTTCCAATTTCAATGGAAATGAGACAAGGTCCATATGGAATTATTGAATCTCATGGTGATAGATGGATTCAGCAAAGAAGATTTGCATTGCATATTCTTCGAGATTTTGGATTGGGAAAGAATTTGATGGAAGAAAAGGTTCTTGGTGAAGTTACTGCAATGATTGACAGTATTCGAAAAAGCATGGAAGATGTTGATATGCAAAATATATTTGATGCATCCGTGGGATCCGTAATCAATAATATGCTTTTTGGATATCGTTATGATGAGACAAACATTGAAGAATTTCTGGAACTCAAAAATCGAATGAATAAGCATTTCAAGTTAGCTGCAGAACCAATGGGAGGACTTATTGGAATGAATCCATGGCTTGGACATCTCCCGTTTTTCAAGGGATATAAAAATGTAATAATGCACAACTGGATGGGTCTCATGGAAATGTTCCGTAAGCAGGCTACAGATAGACTTGCATCAATTGACTATGATTCTGATGAATATTCGGATTACGTAGAAGCATTTTTGAAAGAAAGAAAGAAACATGAAAATGAGCAAGATTTCGGAGGATTTGAAATGGAACAACTTGATAGTGTATGTTTTGATCTATGGGTTGCTGGAATGGAAACAACTTCAAATACTCTCAATTGGGCACTTCTCTATGTCCTTTTGAATCCAGAAGTACGTCAAAAAGTCTATGAAGAATTGGAAAGAGAAATTGGAAGTGATAGGATTATTACAACTACCGACCGACCTAAACTCAACTATATCAATGCAACTGTTAATGAATCTCAACGTCTTGCTAATCTTCTTCCAATGAATCTATCTAGATCTACAAATGCTGATGTTGAAATTGCTGGCTACCGTATTCCAAAAGATACTGTAATCACTCCACAAATCAGTTCAGTAATGTATGATCCAGAGATATTCCCTGAACCATACGAATTCAAACCTGAAAGATTCCTAGAATCGGATGGAAGTTTGAAAAAAGTAGAAGAACTTGTGCCATTTTCAATTGGAAAACGGCAATGTCTTGGAGAAGGACTGGCCAAAATGGAACTATTCTTGTATTTTGCAAATTTGTTCAACAAATTTGATATTAAATTTCACGAATCGAATCCAAATCCAAGTATTAAAAAGGAAGTCGGAGTTACTATGAAGGCAAAGAATTACAGAGTTTCAATGAAGGAAAGATATTGAagaggcggccgc

**LMP-1 Constructs;**

NM1553, LMP-1/pGEX4T-1, included a N-terminus GST tagged domain of LMP-1, amino acids 18-160 (gift from A. Audhya).

NM1552, LMP-1/pRSETA, included a N-terminus His6 tagged domain of LMP-1 from NM1553. NM1553 was digested with BamHI/XhoI and the fragment was cloned into the BamHI/XhoI site of the vector pRSETA.

NM1552 insert sequence:

ATGCGGGGTTCTCATCATCATCATCATCATGGTATGGCTAGCATGACTGGTGGACAGCAAATGGGTCGGGATCTGTACGACGATGACGATAAGGATCGATGGGGATCCACCGCTTCGCATTACTATGTAACCAACAACAACACCGGACTCACGTGCATCATCCTCGACGGAGACTTCCAGTTCAATTTGGTCTTCAACGAGAAGAACACCACCGAGAAGTTCACGGTCACATTCAACGAGACAGTCAGTGTCGAAGGAGATTGCAACGGAGTCCGCAACAATCAATCGGTTCAGACGTTGAATATTAAGTTCAACCCTGAAGGACAATCTGCCCGTTACGCCAAAGAGTGGGAGCTCGATATCGTGTTCGGATCATCTAGCAACGTCGCCTTTGAGATCATTGACTACACCCTCACCACTCAGAGAACGGATCTTGTCCCATACTTTGGAAAGTTTGTCAGAGACGCCAATGCTGCCGGAGATGTTACAGCAACTCAGACCAACGCTTACAAGTGCTCCACCGCCAAGCTTGGACTCGAGATCTGCAGCTGGTACCATGGAATTCGAAGCTTGATCCGGCTGCTAACAAAGCCCGAAAGGAAGCTGAGTTGGCTGCTGCCACCGCTGAGCAATAACTAG

**RME-1 Constructs:**

NM1450, pET24b:RME-1, included a C-terminus His6 tagged RME-1d, amino acids 333-575 (gift from B. Grant).

NM1791, RME-1/pGEX4T-1, included a N-terminus GST tagged RME-1d from NM1450. Primers, #3178: 5’-AGGGGATCCGCGATGCAAATGCCATCGATGATC and #3179: 5’-GGCGAATTCTTGTCAATCGTTGTCGTTGAGCGA, were used. The fragment was cloned into the BamHI/EcoRI site of the vector pGEX4T-1

NM1791 insert sequence:

ATGTTCGAAGATCGTTTATGTCATAAAACATATTTAAATGGTGATCATGTAACCCATCCTGACTTCATGTTGTATGACGCTCTTGATGTTGTTTTATACATGGACCCAATGTGCCTGGATGCGTTCCCAAAATTAGTTTGTTTTAAAAAACGTATTGAAGCTATCCCACAAATTGATAAGTACTTGAAATCCAGCAAGTATATAGCATGGCCTTTGCAGGGCTGGCAAGCCACGTTTGGTGGTGGCGACCATCCTCCAAAATCGGATCTGGTTCCGCGTGGATCCGCGATGCAAATGCCATCGATGATCGGAAAGGATAAGAAGAAGAAGGATTTGATCCAAAATCTTGACAAGATCTACGAGCAACTCCAGAGAGAACACAATATATCTCCAGGAGACTTCCCAGATGTGAACAAGATGAGAGAGAAGCTTCAAACACAGGACTTTAGCAAATTCAATCCACTCAAGCCAAAGCTTTTGGAGGTTGTCGATGGAATGCTTGCCACTGATATAGCACGATTGATGGCTCAAATTCCAAAGGAAGAAGCCGCAGCCCCAGCAGGATCAAACGGAAGCGCCGATCCAACTGTCAGAGGAGGAGCCTTCAGCCAAACAACAGAAGCAGAAACTCCGTTCGGTTTCGGACGTGGAGAAGGATTCGACAAGGGAGCCGACGAAGCCGAATGGGTTGTTAGTCGGGAACGCACAACTGCCGACTCGACATTCGAAAGTCTCGGACCAGTTAACGGATATCTCAGCGGAAGAGCTGCCAAGGAGCACATGGTTAAGAGCAAATTACCAAACTCAGTGCTCGGAAAAGTATGGAAATTGGCTGATATTGACAAGGATGGACAGTTGGATGCTGACGAGTTTGCACTTGCCAACTATTTGATCAATTTGAAATTGGAAGGACACGAGATTCCAAGTGAGCTGCCAAAACATCTCATCCCACCATCAAAACGTGGAGAGCAAGATCCAGTCTACCCATCGCTCAACGACAACGATTGAcaagaattc

**SQV-8 Constructs:**

NM1740, SQV-8/pGEX6P-1, included a N-terminus GST tagged domain of SQV-8, amino acids 150-349 (gift from A. Audhya).

NM1592, SQV-8/pRSETA, included a N-terminus His6 tagged domain of SQV-8 from NM1740. NM1740 was digested with BamHI/XhoI and the fragment was cloned into the BamHI/XhoI site of the vector\ pRSETA.

NM1592 insert sequence:

ATGCGGGGTTCTCATCATCATCATCATCATGGTATGGCTAGCATGACTGGTGGACAGCAAATGGGTCGGGATCTGTACGACGATGACGATAAGGATCGATGGGGATCCTCAAAAATTCCAAATACACATCTAAATGCTCGAACTCCGTCTGATCAAAAAATGAGATACGATGACCCCAATTGGACGTTGCCACGTGGCGTTGAGCAACGAAATCGAGCACTTTTATGGATTCAAAATCAATTGAGTGGTGTGAAAGAGGGCGTTGTGTACTTTGGAGATGATGATAACACATATGATCTGAAAATTTTCGGAGAAATGCGGAAAGTGAAAAATGCAGGAGTTTGGCCAGTTGGAATAGTTGGTGGAATGTTTGTAGAAACGCCAATTTTAGAGAAAAATGGATCGATTTCCCATTTCAACGCTGTCTGGAAACCGGAGCGTCCATTTCCGATCGATATGGCTGCATTTGCAGTCAATATTTCTCTGGTTCTCTCCAACGCGAACGCTCTTTTCTCATTCGACGTGCCTCGTGGATATCAAGAATCTACTTTTCTTGAAAATCTTGGAATTCATCGTTATAATATGGAACCACTTGCGGAAATGTGCACGAAAGTGTATGTGTGGCATACGAGAACTGAAAAACCGAAATTGTCGAAAGAATCGATTGATCGATTGACTAAAAAGACAGGATTCAACTCGTTAGAAGCATAActcgag

**DYN-1 Construct:**

Protein (gift from Z. Zhou and N. Lu)

**APA-2 Constructs:**

NM1460, pET24b:APT-4, included a C-terminus His6 tagged APA-2, amino acids 618-925 (gift from B. Grant).

NM1853, APT-4/pGEX4T-1, included a N-terminus GST tagged APA-2 from NM1460. Primers, #3159: 5’-CCGGAATTCCCGATGTCTAAGCCACAACTGGAAGAAAT and #3160: 5’-GGCCGCTCGAGCGGTTAAAATTGGTTGCCCAATAAGTC, were used. The fragment was cloned into the EcoRI/XhoI site of the vector pGEX-4T-1.

NM1853 insert sequence:

ATGTTCGAAGATCGTTTATGTCATAAAACATATTTAAATGGTGATCATGTAACCCATCCTGACTTCATGTTGTATGACGCTCTTGATGTTGTTTTATACATGGACCCAATGTGCCTGGATGCGTTCCCAAAATTAGTTTGTTTTAAAAAACGTATTGAAGCTATCCCACAAATTGATAAGTACTTGAAATCCAGCAAGTATATAGCATGGCCTTTGCAGGGCTGGCAAGCCACGTTTGGTGGTGGCGACCATCCTCCAAAATCGGATCTGGTTCCGCGTGGATCCCCGGAATTCCCGATGTCTAAGCCACAACTGGAAGAAATTGAGCGTGAGGAGAAGGAGAAACGGTCCAAACCAACTGCAGTAATGAGCGAAGGATCAACATCTCTTGTGGACTTTGATTCTACCAATGACACTACTGCATCGCTAGCCGACGTTTTCGCTAACAACTCTGGAACCGGTCTTGGAGCACAAGGTGATGAAGTCGAGATTGCCAACAAGGATGACTACTTGAAGTTTGTCACGAAAAGTAACGCAATTTTGTGGGAGGACGATTACATTCAAATTGGCTGTAAATTGGAGACGCGCAATAACTTGGGCCGACTGGGAATGTTCTATGGAAATAAGACGTCGCAACCATTTAATAAGTTCACCCCAATCATCACGTGCCCTGGAGCACTAGCAGTTCAACTACAAGCTCAGGCAAAACCAGTGGAGCCGGTGGTTGCAGCTGGAACTCAAGTTCAGCAATTGATTAATTTTGTCTGTGTTCAAGAGTTCCAGAAGATGCCAATTATGAACATAAAGTTTACATTCACAGATCGTGCGGGTGCTGTTCAAAACTTTGATAAAAACTTCTACCTTCCACTCTTCATCAGCAAATTCTTCGAGCCCACAAACATGACATCCGAGCAATTTTTCACTAGATGGAAGTCACTTGGTGCAGCGTCACAGGAAGCACAGAAGATTTTCAACGCACTGTCACCGATTGAGCACGCAACTATTGAATCAAGACTGAAAGGATTTGGAGCAAACTTACTGACTGACGTGGATCCAAATCCCGACAATTATGTGTGCGCTGGAATCATTCATACCCAGACGCAACAGATTGGAACTCTTATTCGTTTGGAGCCAAACAAGCAAGCAAAGATGTACCGCCTTACAATCCGTTCCAGCAAGGACACTGTTGTGCAGACATTGGTAGACTTATTGGGCAACCAATTTTAAccgctcgag

**Immunization:** Three female inbred BALB/c mice (Taconics, Hudson, NY), 5-6 weeks of age, were immunized subcutaneously with a 1:1 emulsified mixture of recombinant fusion protein in phosphate-buffered saline, pH 7.4 (PBS) at 50 μg per mouse with complete Freund’s adjuvant (Sigma-Aldrich, St Louis, MO). Booster immunizations were done subcutaneously with a 1:1 emulsified mixture of the fusion protein in PBS at 50 μg per mouse with incomplete Freund’s adjuvant (Sigma-Aldrich). The Washington University Division of Comparative Medicine (DCM) animal facility housed the animals and administered all injections. To assess immunity and titer levels, both indirect ELISA with the fusion protein as the coating antigen and epi-fluorescent microscopy with fixed worms were performed. A final challenge injection of the fusion protein at 37.5 μg in PBS was done intravenously or intraperitoneally, 3-4 days prior to the fusion of the splenic lymphocytes with the myeloma cells.

SNB-1: A mouse was injected with three boosts of the fusion protein consisting of a 50:50 mix of native:denatured protein, with an IV challenge (Injections spaced at: 2 wk, 2.5 wk, 3 wk, 5 wk (IV fuse)). A total of 237.5 μg of fusion protein was injected.

UNC-10: A mouse was injected with three boosts followed by an IV challenge with a total of 237.5 μg of fusion protein (Injections spaced at: 2 wk, 3 wk, 2.5 wk, 3.5 wk (IV fuse)).

DLG-1: A mouse was injected with four boosts of the fusion protein, followed by an IP injection with the GST-tagged fusion protein (Injections spaced at: 2 wk, 2 wk, 3 wk, 3.5 wk, 8.5 wk (IP fuse)). A total of 287.5 μg of fusion protein was injected.

HMR-1: A mouse injected with the fusion protein had a total of four boosts plus an IP challenge. A total of 287.5 μg of fusion protein was injected (Injections spaced at: 2 wk, 2 wk, 4.5 wk, 4 wk, 5 wk (IP fuse)).

ERM-1: A mouse was injected with the His6-tagged ERM-1 fusion protein for a total of five boosts, followed by an IP injection (Injections spaced at: 2 wk, 2 wk, 3.5 wk, 4 wk, 4.5 wk, 5.5 wk (IP fuse)). A total of 337.5 μg of fusion protein was injected.

LET-413: A mouse was injected with four boosts, plus an IP challenge (Injections spaced at: 2 wk, 2 wk, 4.5 wk, 4 wk, 6 wk (IP fuse)). A total of 287.5 μg of fusion protein was injected.

SAX-7: A mouse was injected with six boosts, plus an IP challenge (Injections spaced at: 2 wk, 2 wk, 3.5 wk, 4 wk, 4.5 wk, 17 wk, 11 wk (IP fuse)). A total of 387.5μg of fusion protein was injected.

HCP-4: A mouse was injected with a total of five boosts, plus an IV challenge. A total of 337.5 μg of fusion protein was injected (Injections spaced at: 2 wk, 2 wk, 3 wk, 3 wk, 3.5 wk, 5.5 wk).

LMN-1: A mouse was injected with five boosts of the fusion protein, followed by an IP challenge with the GST-LMN-1 fusion protein (Injections spaced at: 2 wk, 2 wk, 2.5 wk, 3 wk, 9.5 wk, 27 wk (IP fuse)). A total of 337.5 μg of fusion protein was injected.

DAO-5: A mouse was injected with five boosts followed by an IP injection with the GST-tagged fusion protein (Injections spaced at: 2 wk, 2 wk, 2.5 wk, 4.5 wk, 4 wk, 5.5 wk (IP fuse)). A total of 337.5 μg of fusion protein was injected.

ORC-2: A mouse was injected with eight boosts, followed by an IP injection (Injections spaced at: 2 wk, 2 wk, 3 wk, 3 wk, 4 wk, 2.5 wk, 21.5 wk, 3.5 wk, 4.5 wk (IP fuse)). A total of 487.5 μg of fusion protein was injected.

PAS-7: A mouse used for making these antibodies was injected with four boosts, followed by an IP challenge (Injections spaced at: 2 wk, 2 wk, 3 wk, 3 wk, 4 wk (IP fuse)). A total of 287.5μg of fusion protein was injected.

TAC-1: A mouse was injected with five boosts followed by an IP challenge with 337.5 μg of fusion protein (Injections spaced at: 2 wk, 2 wk, 2 wk, 2.5 wk, 18 wk (IP fuse)).

HSP-60: A mouse was injected with five boosts followed by an IP injection with the GST-tagged fusion protein (Injections spaced at: 2 wk, 2 wk, 2.5 wk, 3 wk, 3 wk, 33 wk (IP fuse)). A total of 337.5 μg of fusion protein was injected.

CAV-1: A mouse was injected with a total of five boosts with the fusion protein followed by an IP challenge with a total of 337.5 μg protein (Injections spaced at: 2 wk, 2 wk, 2.5 wk, 3 wk, 22.5 wk, 28 wk (IP fuse)).

CYP-33E1: A mouse was injected with four boosts followed by an IP injection (Injections spaced at: 2 wk, 2 wk, 3 wk, 3 wk, 5 wk (IP fuse)). A total of 287.5 μg of fusion protein was injected.

LMP-1: A mouse injected with this fusion protein had a total of four boosts, plus an IV challenge. A total of 287.5 μg of fusion protein was injected (Injections spaced at: 2 wk, 2 wk, 3 wk, 3 wk, 4 wk (IV fuse)).

RME-1: A mouse injected with this fusion protein had a total of four boosts, plus an IV challenge. A total of 287.5 μg of fusion protein was injected (Injections spaced at: 2 wk, 2 wk, 2.5 wk, 3 wk, 4 wk (IV fuse)).

SQV-8: A mouse was injected with four boosts followed by an IP injection with the GST-tagged fusion protein (Injections spaced at: 2 wk, 2 wk 3 wk, 16 wk (IP fuse)). A total of 287.5 μg of fusion protein was injected.

DYN-1: A mouse used for making the monoclonal antibodies was injected with four boosts, followed by an IV injection (Injections spaced at: 2 wk, 2 wk, 3 wk, 3 wk, 5 wk (IV fuse)). A total of 287.5 μg of fusion protein was injected.

APA-2: A mouse was injected with five boosts followed by an IP injection (Injections spaced at: 2 wk, 2 wk, 4 wk, 3 wk, 7 wk, 5 wk (IP fuse)). A total of 337.5 μg of fusion protein was injected.

**REFERENCES:**

1. Fasshauer D, Otto H, Eliason WK, Jahn R, Brunger AT (1997) Structural changes are associated with soluble N-ethylmaleimide-sensitive fusion protein attachment protein receptor complex formation. J Biol Chem 272: 28036-28041.
