# Supplementary material for: A Monoclonal Antibody Toolkit for C. elegans
Source: PLoS One. 2010 Apr 13;5(4):e10161. doi: 10.1371/journal.pone.0010161 (PMC2854156; doi:10.1371/journal.pone.0010161)
Supplement: Table S1 — List of the Supplemental mAbs. (0.07 MB DOC) [file pone.0010161.s003.doc]

Supplemental Table 1:

Supplemental Monoclonal Antibodies

|  | Whole mount ISH | | Westerns | | Isolate |  |
| --- | --- | --- | --- | --- | --- | --- |
| Antigen | Expected | Observed | Expected | Observed | name | comments |
| UAF-1 | Spliceosomes; likely speckles in nuclei | Speckled nuclei | 16 kd, 53 kd, and 55 kd | 120 kD, 70 kD, 68 kD, 64 kD, 38 kD, and 16 kD;  250 kd from fixed extracts of fixed animals. | 3C5 | Published polyclonal sera behave as expected |
| NCA-1 | Neuronal plasma membrane | ER of many cell types | 222 kd | 220 kd | 3G12 | Published polyclonal sera behave as expected |
| HIM-3 | Meiotic germ cells | Germline nuclei, Neuronal processes | 33 kd | 92 kd | 11.12.2 | Published polyclonal sera behave as expected |
| SQV-2 | Golgi | Vulva and around the nucleus of cells | 38 kd | 47 kd | 10D11 | Reacts strongly with protein markers  published polyclonal sera behave as expected |
| AEX-6 | Dense core vesicles | Body wall muscle, sphincter and anal depressor muscle, faint labeling of head neurons | 24 kd | ------- | 12H3 | Published polyclonal sera behave as expected |
| RAB-3 | Synaptic vesicles | Muscle; pharynx; unknown cells | 25 kd | 250 kd | 13G8 | Published polyclonal sera behave as expected |
| UNC-64 | Neuronal plasma membrane; apical domain in intestine | Neuronal staining; intestinal nuclei, muscle | 33 kd | 20 kd, 36 kd, 56 kd, and 68 kd | 5H1 | Published polyclonal sera behave as expected. 5E3 could detect specifically a syntaxin complex. |
| UNC-29 | Nervous system; cholinergic; postsynaptic | Nervous system and muscle | 56 kd | 110 kd | 5H1 | Published polyclonal sera behave as expected |
| SAS-5 | centriole | nucleus | 46 kd | 1B1: < 30 kd and >300 kd  7B2: 37 kd and 88 kd | 1B1 and 7B2 | Published polyclonal sera behave as expected |
| EEA-1 | Coritical puncta; early endosomes | Unknown cells | 140 kd | 10 kd, 15 kd, 25 kd, 100 kd, 150 kd, and 250 kd | 2C2 | Published polyclonal sera behave as expected |
| RME-2 | Developing oocytes and in the proximal region of germ line; recycling endosomes | Muscle, pharynx, oocytes uterus, and unknown cell types | 103 kd | -------- | 4C3 | Published polyclonal sera behave as expected |
| RME-8 | Hypodermis, muscle, gonad, spermatheca, and intestine; late endosomes | Hypodermis, muscle, gonad, spermatheca, and intestine | 258 kd and 259 kd | ------- | 9B4 | Published polyclonal sera behave as expected |
| PAR-1 | Posterior domain of asymmetrically dividing embryonic cells | Diffuse in embryo | 114 kd, 117 kd, and 126 kd | ------- | 3F10 | Published polyclonal sera behave as expected |
| PAR-6 | Cytoplasm, periphery of embryos, apical regions of intestine, pharynx, and spermatheca; Anterior domain of asymmetrically dividing embryonic cells | Cytoplasm, periphery of embryos, apical regions of intestine, pharynx | 20 kd and 334 kd | 33 kd, 38 kd, and 125 kd | 8H8 | Published polyclonal sera behave as expected |
| GLR-1 | Nervous system; glutamatergic; postsynaptic | Muscle, nervous system, and unknown cells | 108 kd | 76 kd | 4G11 |  |
| EAT-4 | Nervous system-glutamatergic neurons; presynaptic | Muscle and unknown cells | 63 kd | 75 kd and 115 kd | 4B10 |  |
| TRAP-2 | Endoplasmic rough reticulum (RER) | Diffuse | 22 kd | ------ | 7D6 |  |
| CUP-5 | Coelomocytes, cytoplasmic vesicles; lysosomes | Spermatocytes, gonad | 70-76 kd | 75 kd | 5G10 | Published polyclonal sera behave as expected |
| ELKS-1 | Nervous system; active zones | Gonad, muscle, nervous system, and unknown cells | 96 kd | 73 kd | 2A9 | Published polyclonal sera behave as expected |
| TAC-1 | centrosomes | nuclei | 29 kd | 1E9: 10-15 kd, 100 kd, and 250 kd  2A3: 10-20 kd, and 250 kd | 1E9 | Published polyclonal sera behave as expected |

Expected Mw predicted from EXPASY ([www.expasy.ch/tools/pi_tool.html](http://www.expasy.ch/tools/pi_tool.html))
